# Inhibitory Response to CK II Inhibitor Silmitasertib and CDKs Inhibitor Dinaciclib Is Related to Genetic Differences in Pancreatic Ductal Adenocarcinoma Cell Lines

**DOI:** 10.3390/ijms23084409

**Published:** 2022-04-16

**Authors:** Yixuan Ma, Sina Sender, Anett Sekora, Weibo Kong, Peter Bauer, Najim Ameziane, Susann Krake, Mandy Radefeldt, Ruslan Al-Ali, Frank Ulrich Weiss, Markus M. Lerch, Alisha Parveen, Dietmar Zechner, Christian Junghanss, Hugo Murua Escobar

**Affiliations:** 1 Department of Medicine Clinic III, Hematology, Oncology and Palliative Medicine, Rostock University Medical Center, 18057 Rostock, Germany; yixuan.ma@med.uni-rostock.de (Y.M.); sina.sender@med.uni-rostock.de (S.S.); anett.sekora@med.uni-rostock.de (A.S.); kong@fbn-dummerstorf.de (W.K.); peter.bauer@centogene.com (P.B.); christian.junghanss@med.uni-rostock.de (C.J.); 2 Institute of Muscle Biology and Growth, Research Institute for Farm Animal Biology (FBN), 18196 Dummerstorf, Germany; 3 CENTOGENE GmbH, 18057 Rostock, Germany; najim.ameziane@arcensus-diagnostics.com (N.A.); susann.krake@centogene.com (S.K.); mandy.radefeldt@centogene.com (M.R.); ruslan.al-ali@centogene.com (R.A.-A.); 4 Arcensus GmbH, 18055 Rostock, Germany; 5 Department of Medicine A, University Medicine, University of Greifswald, 17475 Greifswald, Germany; ulrich.weiss@med.uni-greifswald.de (F.U.W.); markus.lerch@med.uni-muenchen.de (M.M.L.); 6 LMU Munich University Hospital, 81377 Munich, Germany; 7 Institute for Experimental Surgery, University of Rostock, 18057 Rostock, Germany; alisha.parveen@med.uni-rostock.de (A.P.); dietmar.zechner@uni-rostock.de (D.Z.)

**Keywords:** casein kinase II, cyclin dependent kinase, pancreatic ductal adenocarcinoma, *KRAS*, *TP53*

## Abstract

Casein kinase II (CK2) and cyclin-dependent kinases (CDKs) frequently interact within multiple pathways in pancreatic ductal adenocarcinoma (PDAC). Application of CK2- and CDK-inhibitors have been considered as a therapeutic option, but are currently not part of routine chemotherapy regimens. We investigated ten PDAC cell lines exposed to increasing concentrations of silmitasertib and dinaciclib. Cell proliferation, metabolic activity, biomass, and apoptosis/necrosis were evaluated, and bioinformatic clustering was used to classify cell lines into sensitive groups based on their response to inhibitors. Furthermore, whole exome sequencing (WES) and RNA sequencing (RNA-Seq) was conducted to assess recurrent mutations and the expression profile of inhibitor targets and genes frequently mutated in PDAC, respectively. Dinaciclib and silmitasertib demonstrated pronounced and limited cell line specific effects in cell death induction, respectively. WES revealed no genomic variants causing changes in the primary structure of the corresponding inhibitor target proteins. RNA-Seq demonstrated that the expression of all inhibitor target genes was higher in the PDAC cell lines compared to non-neoplastic pancreatic tissue. The observed differences in PDAC cell line sensitivity to silmitasertib or dinaciclib did not depend on target gene expression or the identified gene variants. For the PDAC hotspot genes kirsten rat sarcoma virus (*KRAS*) and tumor protein p53 (*TP53*), three and eight variants were identified, respectively. In conclusion, both inhibitors demonstrated in vitro efficacy on the PDAC cell lines. However, aberrations and expression of inhibitor target genes did not appear to affect the efficacy of the corresponding inhibitors. In addition, specific aberrations in *TP53* and *KRAS* affected the efficacy of both inhibitors.

## 1. Introduction

Pancreatic ductal adenocarcinoma (PDAC) is one of the most common malignancies and ranks fourth among all cancer-related deaths in both men and women [1]. Due to the lack of effective therapy, tumor metastasis, and chemo resistance, the prognosis of PDAC is poor [2,3,4,5]. Furthermore, the “cure rate” for PDAC is only 9%, and without treatment, the median survival of patients with metastatic disease is only three months [6]. Although extensive research has been carried out in recent years, there were only slight improvements to disease prognosis, median survival is still less than 12 months, and recently, the overall 5-year survival rate only increased to 10% [1].

Casein kinase II (CK2) is a highly conserved serine/threonine kinase, which is constitutively active and ubiquitously expressed in mammalian cells. CK2 has a wide range of candidate physiological targets and is involved in a series of complex cellular functions [7]. For example, CK2 activates protein kinase B (AKT) by direct phosphorylation or indirect regulation [8]. The activated phosphoinositide 3-kinase (PI3K)/AKT pathway influences proliferation and survival [9]. In addition, CK2 upregulates the JAK/STAT and RAS/MEK/ERK signaling pathways and provides survival advantage and proliferative capacity to cancer cells [10,11]. Furthermore, CK2 is able to cooperate with the MKK4/JNK pathway and promotes the survival of PDAC cells [12]. The downregulation of CK2 via RNA interference enhances chemosensitivity to gemcitabine in PDAC cell lines [12,13,14]. Both cell assays and animal models revealed the anti-tumor activity of silmitasertib, a CK2 inhibitor, in BxPc-3 cells [15]. Other CK2 specific-inhibitors also induce the apoptosis of the MIA Paca-2 and Dan-G cell lines [14]. However, in these experiments, different cell lines showed different responses to CK2 inhibitors. Therefore, studying the influence of genetic background on the efficacy of silmitasertib is of considerable interest. In addition, although silmitasertib has entered multiple clinical trials, clinical trials related to pancreatic cancer have not been reported [16].

CK2 is not the only protein kinase that plays a critical role in PDAC. Cyclin-dependent protein kinases (CDKs) are critical regulators of cell cycle progression. This dysregulation of the cell cycle is the fundamental process of cancer growth and spread [17]. Within the CDK family, CDK1 and CDK2 regulate cell cycle progression by contributing to the phosphorylation and inactivation of the retinoblastoma (Rb) tumor suppressor gene product throughout late G1, S, and G2-M phases [18]. Another family member, CDK9, is involved in the regulation of RNA polymerase II and the control of cellular transcription [19]. CDK5 has been well characterized for its role in the central nervous system rather than the cell cycle [20]. Several CDK family members are highly expressed in different cancer types including PDACs [21]. Moreover, some studies have indicated that CDKs play critical roles in cancer proliferation, migration, invasion, and metastasis [22,23]. In addition, inhibition or knockdown of CDKs demonstrated satisfactory inhibition of cancer cells. Inhibition of CDK1, CDK2, and CDK9 caused cell cycle arrest [24,25,26]. Activated CDKs induce resistance to cisplatin in cervical cancer and are involved in radiation resistance in lung cancer [27,28]. In PDAC cell lines, inhibition of CDKs’ kinase activity significantly decreased the migration and invasion of cancer cells in vitro [22]. In addition, CDK5 inhibition promotes the chemosensitivity of PDAC cell lines to gemcitabine in vivo [22]. A combination of CDKs and AKT inhibitors has been shown to dramatically block PDAC tumor growth and metastasis in vivo [23]. Although the inhibition of CDKs by dinaciclib has been shown to inhibit the viability of PDAC in both cellular and animal models, the observed effect is highly variable, depending on different cell lines and CDK inhibitors, respectively. So far, the reasons for these differences are still not fully understood [23,29,30]. However, based on the significant effects of CDK inhibitors, several inhibitors alcociclib (flavopiridol), dinaciclib, ibociclib, and AT7519 have entered several clinical trials against PDAC [21,31,32].

Different genetic aberrations affecting direct drug target genes, downstream pathways, or key oncogenic regulators also have an impact on drug efficacy. *KRAS* and *TP53* are two of the hotspot genes frequently mutated in PDAC. It has been reported that *KRAS* and *TP53* mutations can be found in approximately 92% and 70% of PDAC patients, respectively [33,34]. Moreover, patients with *KRAS* mutations showed a bad response to first-line gemcitabine-based therapy and represented a poor prognosis [35]. PDAC patients with regular *TP53* expression were reported to show a significant improvement in progression-free survival when compared to complete loss. Interestingly, cases showing as many as two *TP53* somatic variants are reported to have a better prognosis than when compared to cases exceeding accumulation of more than three somatic variants [36,37]. Therefore, due to the impact of *KRAS* and *TP53* on the prognosis and drug efficacy of PDAC, we explored the influence of the somatic variants of these two genes on the response of PDAC cell lines to CK2 and CDK inhibitors.

Akin to the above-mentioned studies, several other publications have demonstrated the influence of CK2 and CDKs on the pathophysiology of PDAC. However, for these drug target genes, it is still poorly understood if and which somatic variants affect sensitivity to the respective inhibitors [14,23]. In general, CK2 and CDK gene expression does not vary significantly in most mammalian tissues and species [38]. We therefore investigated the effects of the CK2 inhibitor (silmitasertib) and CDK1/2/5/9 inhibitor (dinaciclib) in ten PDAC cell lines (AsPc-1, BxPc-3, Capan-1, Panc-1, PaTu8902, PaTu8988T, PaTu8988S, SU.86.86, T3M4, and Colo357). In order to evaluate gene expression and gene variants in these cell lines, whole transcriptome and whole exome sequencing (WES) were performed with the aim to explore the relationship between the sensitivity of these inhibitors and the gene expression of inhibitor targets and mutations in *KRAS* oncogene and *TP53* tumor suppressor genes.

## 2. Results

### 2.1. Effects of Silmitasertib and Dinaciclib on Cell Proliferation, Biomass, and Metabolic Activity

**Figure 1:**
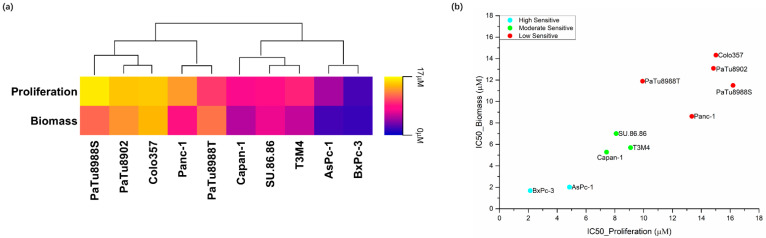
IC_50_ values when assessing proliferation and cell biomass after 72 h to silmitasertib exposure in ten PDAC cell lines (**a**) as well as the classification of these cell lines by k-means++ (unsupervised machine learning algorithm) to a low (red), moderate (green), and high (blue) sensitivity group (**b**).

The CK2 inhibitor silmitasertib significantly inhibited cell proliferation of PDAC cell lines starting from 1 μM for the most sensitive cell line BxPc-3. Meanwhile, significant inhibition of biomass was observed with silmitasertib in all cell lines tested, with AsPc-1 and BxPc-3 significantly inhibited from 1 μM. Moreover, silmitasertib significantly reduced the metabolic activity of cells, with the majority of PDAC cell lines (eight out of ten) initiated significant reductions at 5 μM (Supplementary Figure S1 and Table S1). The IC_50_ values for proliferation and biomass showed a range from 2.131 μM to 16.20 μM for proliferation and a matching range from 1.691 μM to 14.32 μM for biomass (Figure 1a, Supplementary Figure S2 and Table S2).

IC_50_ values of cell proliferation and biomass were applied in the following bioinformatic clustering (k-means++ clustering method, Materials and Methods 4.10) for sensitivity classification of cell lines. Ten PDAC cell lines were separated into three groups with low (PaTu8988S, Panc-1, PaTu8988T, PaTu8902, and Colo357), moderate (Capan-1, T3M4, and SU.86.86), and high sensitivity (AsPc-1 and BxPc-3) (Figure 1b and Supplementary Figure S2).

**Figure 2:**
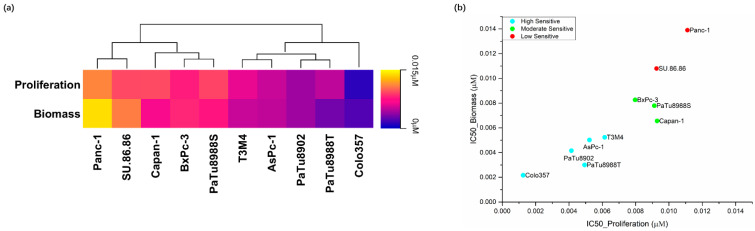
IC_50_ values when assessing proliferation and cell biomass after 72 h dinaciclib exposure in ten PDAC cell lines (**a**) as well as the classification of these cell lines by k-means++ (unsupervised machine learning algorithm) to a low (red), moderate (green), and high (blue) sensitivity group (**b**).

The CDK1/2/5/9 inhibitor dinaciclib significantly inhibits the cell proliferation, metabolic activities, and biomass of all PDAC cell lines starting from the lowest tested concentration (0.001 μM), but responses varied between cell lines (Supplementary Figure S3 and Table S3). At the lowest tested concentration, Colo357, PaTu8988T, and T3M4 observed significant inhibition in cell proliferation assays; Colo357, PaTu8988S, and T3M4 observed significant inhibition in metabolic activity assays; and Capan-1, Colo357, and PaTu8988T observed significant inhibition in biomass assays. The IC_50_ values ranged from 0.001253 μM to 0.01111 μM (proliferation) and 0.002146 μM to 0.01390 μM (biomass) (Figure 2a, Supplementary Figure S4 and Table S4).

IC_50_ values of cell proliferation and biomass were applied in bioinformatics clustering (k-means++ clustering method, Materials and Methods 4.10) for sensitivity classification of the cell lines. Ten PDAC cell lines were separated into three groups with low (Panc-1 and SU.86.86), moderate (BxPc-3, Capan-1 and PaTu8988S), and high (AsPc-1, Colo357, PaTu8902, PaTu8988T, and T3M4) sensitivity (Figure 2b and Supplementary Figure S4). Both herein used compounds were reported to be well tolerated in vivo [15,39]. The ranges of inhibitor concentrations were below the maximum plasma concentration.

### 2.2. Silmitasertib and Dinaciclib Induced Cell Deaths in PDAC Cell Lines

Silmitasertib only increased the percentage of cell deaths in two out of ten PDAC cell lines after 72 h. Significant increases in apoptotic/necrotic cells were only observed in AsPc-1 and T3M4 at a concentration of 10 μM (Supplementary Figures S5 and S7 and Table S5). The percentages of apoptotic/necrotic cells in AsPc-1 and T3M4 at 10 μM were 23.13% and 29.33%, respectively. However, significant increases in apoptotic/necrotic cells were not observed in other PDAC cell lines. At the same time, we observed that silmitasertib even significantly reduced cell death in Colo357 when compared to the DMSO control, but due to the low percentages of cell death; this reduction is more like a mathematical artifact.

Dinaciclib strongly induced apoptosis/necrosis in nine of ten PDAC cell lines in a dose-dependent manner. Only the apoptotic/necrotic induction of PaTu8988T was not significant at the tested concentrations (0.003 μM, 0.005 μM, 0.006 μM, and 0.01 μM). Significant increases in apoptotic/necrotic cells were observed starting at a concentration of 0.0075 μM (Supplementary Figures S6 and S8 and Table S6). Interestingly, in comparison to the DMSO control, decreasing percentages of apoptotic/necrotic cells were observed in PaTu8988S at all tested concentrations (0.005 μM, 0.0075 μM, 0.01 μM, and 0.05 μM).

### 2.3. Expression and Genetic Variants of Silmitasertib or Dinaciclib Target Genes

**Figure 3:**
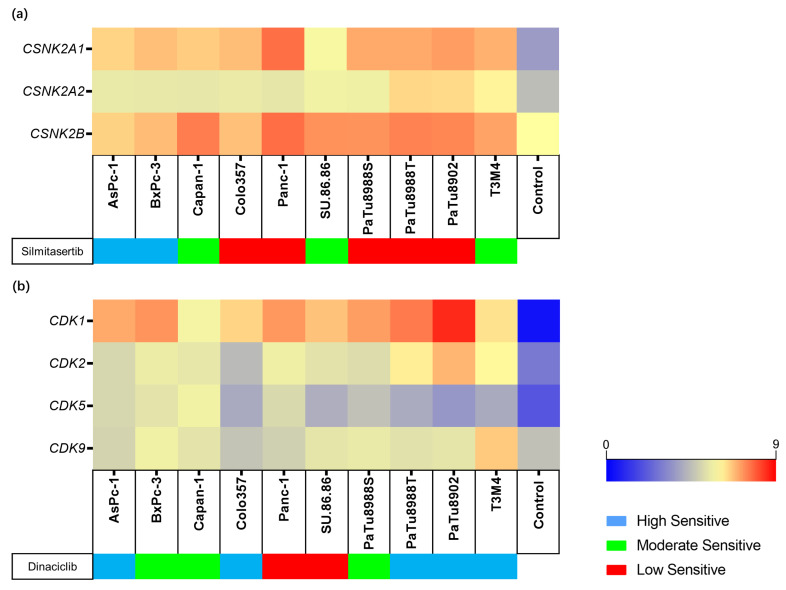
Gene expression levels of inhibitor target genes in the cell lines and control. The different sensitivity to silmitasertib (**a**) and dinaciclib (**b**) is indicated for each cell line. Gene expression levels are displayed as Log2 (TPM+1).

The expression of target genes for each inhibitor (for silmitasertib: *CSNK2A1*, *CSNK2A2*, and *CSNK2B*; for dinaciclib: *CDK1*, *CDK2*, *CDK5*, and *CDK9*) was evaluated in all cell lines by RNA-Seq. The expression level was estimated as Log_2_ (transcripts per kilobase million (TPM) + 1) and compared to the expression data of non-neoplastic pancreatic tissue, which was chosen as a control. All target genes were expressed higher in the PDAC cell lines than in normal pancreatic tissue. The inhibitor target gene expression in PDAC compared with the control are as follows (PDAC Minimum–Maximum vs. control): *CSNK2A1* (5.83–7.69 vs. 3.63), *CSNK2A2* (5.41–6.47 vs. 4.43), *CSNK2B* (6.53–7.52 vs. 6.00), *CDK1* (5.73–8.51 vs. 0.41), *CDK2* (4.37–6.95 vs. 2.83), *CDK5* (3.51–5.32 vs. 1.98), and *CDK9* (4.57–6.63 vs. 4.50) (Figure 3a,b and Supplementary Table S7).

The target genes for silmitasertib (*CSNK2A1*, *CSNK2A2*, *CSNK2B*) and dinaciclib (*CDK1*, *CDK2*, *CDK5*, *CDK9*) were selected to analyze transcript variants by WES.

Focusing on silmitasertib target genes, initially, a total of twenty-four variants including fourteen *CSNK2A1* variants, eight *CSNK2A2* variants, and two *CSNK2B* variants were identified in ten PDAC cell lines in all types of variants (Supplementary Table S8). The initial twenty-four candidate variants were identified in eight cell lines: no variants were identified in Colo357 and SU.86.86; one variant was identified in AsPc-1 and PaTu8902; two variants were identified in Capan-1 and PaTu8988S; three variants were identified in Panc-1; and five variants were identified in BxPc-3, PaTu8988T, and T3M4. Variant filtering according to the Method 4.8 classified none of the identified variants as potentially affecting the protein coding sequence, and as such, presumably leading to aberrant protein function.

When focusing on dinaciclib target genes, a total of fifteen variants including nine *CDK1* variants, four *CDK2* variants, one *CDK5*, and one *CDK9* variant were identified in ten PDAC cell lines (Supplementary Table S9). The initial fifteen candidate variants were identified in eight cell lines: no variants were identified in AsPc-1 and Colo357; one variant was identified in Capan-1, PaTu8902, PaTu8988T, PaTu8988S, and SU.86.86; two variants were identified in BxPc-3 and Panc-1; and six variants were identified in Colo357. Variant filtering according to Method 4.8 classified none of the identified variants as potentially affecting the protein coding sequence in such, presumably leading to aberrant protein function.

### 2.4. KRAS and TP53 Gene Variants Were Observed in PDAC Cell Lines

#### 2.4.1. KRAS Variants and Expression in PDAC Cell Lines

**Figure 4:**
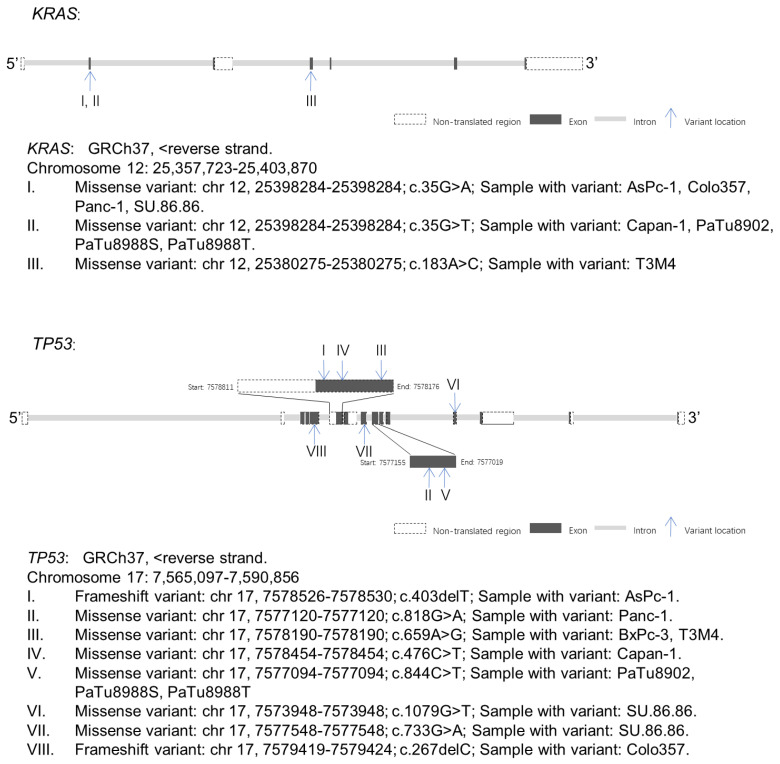
Gene maps indicating the variant sites of *KRAS* and *TP53* in different PDAC cell lines. GRCh37: Genome Reference Consortium Human Build 37, Chr: chromosome.

WES identified *KRAS* variants in nine of the ten tested PDAC cell lines (Figure 4 and Supplementary Table S10). Three different *KRAS* variants were identified, *KRAS* c.35G>A (p.Gly12Asp), *KRAS* c.35G>T (p.Gly12Val), and *KRAS* c.183A>C (p.Gln61His), all of them were missense variants. *KRAS* c.35G>A were observed in AsPc-1 (variant allele frequency (VAF): 100), Colo357 (VAF: 23.8), Panc-1 (VAF: 62.1), and SU.86.86 (VAF: 83.7). *KRAS* c.35G>T were identified in Capan-1 (VAF: 97.1), PaTu8902 (VAF: 100), PaTu8988S (VAF: 96.9), and PaTu8988T (VAF: 98). *KRAS* c.183A>C was identified in T3M4 (VAF: 32.6).

**Figure 5:**
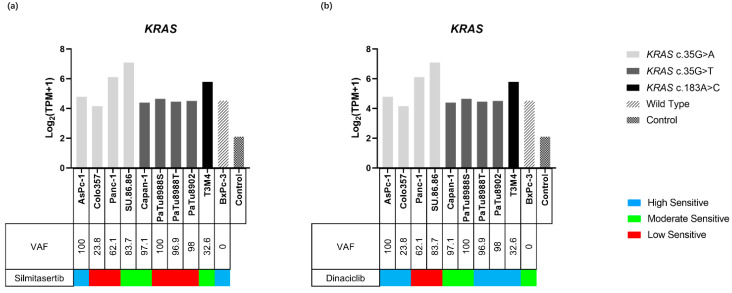
Gene expression of *KRAS* in ten PDAC cell lines and the control. The sensitivity to silmitasertib (**a**) and dinaciclib (**b**) as well as the variants of *KRAS* are indicated for each cell lines. Gene expression levels are displayed as Log_2_ (TPM+1).

The expressions of *KRAS* in all PDAC cell lines were higher than those compared to non-neoplastic pancreas tissue (4.16–7.09 vs. 2.14) (Figure 5 and Supplementary Table S12). Both the lowest and highest *KRAS* expressions were observed in the *KRAS* c.35G>A variant, which were identified in Colo357 (4.61) and SU.86.86 (7.09), respectively. The expressions of all *KRAS* c.35G>T variants, which were identified in Capan-1, PaTu8988S, PaTu8988T, and PaTu8902 were similar to wild type BxPc-3 (4.40, 4.65, 4.46, 4.51 vs. 4.53, respectively), the expression of *KRAS* c.183A>C in T3M4 and *KRAS* c.35G>A in AsPc-1, and Panc-1 and SU.86.86 were higher than wild type BxPc-3 (4.79–7.09 vs. 4.53).

#### 2.4.2. KRAS and Inhibitor Response

A comprehensive analysis of the cell viability assays and *KRAS* status revealed that PDAC cell lines carrying the *KRAS* variant appeared to be less sensitive to silmitasertib and the high sensitive group contained only the wild-type and one *KRAS* mutant cell line, while the rest of the *KRAS* mutant carrying cell lines were all classified into the moderate or low sensitivity groups (Figure 5a). In addition, *KRAS* c.35G single nucleotide variants had no major influence on the inhibitory effect of dinaciclib, since cell lines containing the same *KRAS* c.35G position variant (*KRAS* c.35G>A, *KRAS* c.35G>T) were classified into each of the three sensitivity groups, while wild-type (BxPc-3) was in the moderate sensitivity group. Interestingly, the sensitivity of the *KRAS* c.183A>C mutant cell line (T3M4) was higher than BxPc-3 (Figure 5b). *KRAS* gene expression and VAF did not affect the efficacy of the two inhibitors (Figure 5).

#### 2.4.3. TP53 Variants and Expression in PDAC Cell Lines

**Figure 6:**
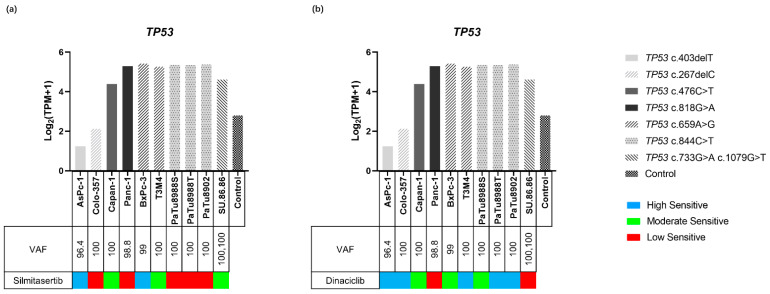
Gene expression of *TP53* in ten PDAC cell lines and the control. The sensitivity to silmitasertib (**a**) and dinaciclib (**b**) as well as the variants of *TP53* are indicated for each cell lines. Gene expression levels are displayed as Log_2_ (TPM+1). Missense variants were associated with gene higher expression while frameshift variants were related to low gene expression.

Two different types of variants including frameshift (fs) variant and missense variant of *TP53* were identified in the PDAC cell lines (Figure 4 and Supplementary Table S11). Fs variants, *TP53* c.403delT (p.Cys135fs) and *TP53* c.267delC (p.Ser90fs), were identified in AsPc-1 (variant allele frequency (VAF): 96.4) and Colo357 (VAF: 100), respectively. Missense variants, *TP53* c.476C>T (p.Ala159Val) and *TP53* c.818G>A (p.Arg273His), were identified in Capan-1 (VAF: 100) and Panc-1 (VAF: 98.8), respectively. Double missense mutation including *TP53* c.733G>A (p.Gly245Ser) and *TP53* c.1079G>T (p.Gly360Val) were identified in SU.86.86 (VAF: 100, 100, respectively). *TP53* c.659A>G (p.Tyr220Cys) was identified in BxPC-3 (VAF: 99) and T3M4 (VAF: 100). *TP53* c.844C>T (p.Arg282Trp) was identified in PaTu8902 (VAF: 100), PaTu8988S (VAF: 100), and PaTu8988T (VAF: 100). The expressions of *TP53* with frameshift variants were lower than that of the missense variants (1.24–2.13 vs. 4.39–5.42) and control (2.83) (Figure 6 and Supplementary Table S13).

#### 2.4.4. TP53 and Inhibitor Response

A comprehensive analysis of cell viability assays and *TP53* status demonstrated that the two cell lines carrying fs variants (Colo357 and AsPc-1) were in the dinaciclib high sensitive group, while cell lines carrying point mutations were distributed in the three sensitivity groups (Figure 6b). However, this effect was not observed when treating the cells with silmitasertib (Figure 6a). In addition, cell lines carrying *TP53* c.844C>T, *TP53* c.818G>A, and *TP53* c.267delC variants were in the low sensitivity group. SU.86.86, which carries two *TP53* missense variants, demonstrated no significant difference in sensitivity to silmitasertib and dinaciclib compared with other cell lines only carrying one variant. *TP53* gene expression and VAF did not affect the efficacy of the two inhibitors (Figure 6).

## 3. Discussion

This study demonstrated that the expression levels of silmitasertib target genes (*CSNK2A1, CSNK2A2,* and *CSNK2B)* in all of the tested PDAC cell lines were higher than in non-neoplastic pancreatic tissue. This result suggests that these cell lines could be sensitive to silmitasertib. Indeed, the inhibition of CK2 by silmitasertib significantly affected cell proliferation of all cell lines except Panc-1, and significantly reduced the cell biomass in all PDAC cell lines. However, silmitasertib did not perform well in reducing the metabolic activity of the PDAC cell lines, and the effects of silmitasertib in inducing apoptosis were also very weak, with significant effects only observed in AsPc-1 and T3M4, which indicates that silmitasertib may inhibit the proliferation of PDAC cells by inducing cell-cycle arrest or cell autophagy rather than apoptosis [40]. Moreover, the cell responses to silmitasertib presented an obvious difference among PDAC cell lines. PDAC cell lines including PaTu8988T, Panc-1, PaTu8902, Colo357, and PaTu8988S represented low responses to silmitasertib inhibition. Although twenty-four variants of CK2 genes in PDAC cell lines were identified, after the filtering step, all variants were excluded for further analysis. In addition, no correlation was seen when comparing the expression of CK2 genes in high-, moderate-, and low-sensitive cell lines. These results indicate that the genes directly targeted by silmitasertib are not directly affected by aberrations modulating the observed antitumor effects of silmitasertib on CK2.

Inhibition of CDKs by dinaciclib dramatically reduced cell proliferation, metabolic activities, and biomass in PDAC cell lines and this significant effect could be observed at nanomolar concentrations. These results are similar to previous reports that suggested that dinaciclib could be a candidate for novel treatment options in PDAC [23,41]. Furthermore, compared with the DMSO control group, dinaciclib was able to increase the percentage of apoptotic/necrotic cells in PDAC cell lines except in PaTu8988S. This suggests that in addition to inducing apoptosis, dinaciclib may inhibit cell proliferation by other mechanisms, but further experiments are still needed for this to be proven [42]. RNA-Seq results demonstrated that expressions of *CDK1/2/5/9* were higher than the control in all tested PDAC cell lines, indicating overexpression of *CDK1/2/5/9* in PDAC cell lines. Moreover, as the experimental results suggest that dinaciclib inhibited cell viability at very low concentrations, the overexpression of target genes appeared to not affect the efficacy of dinaciclib in inhibiting the viability of PDAC cells. Therefore, dinaciclib is an excellent candidate for PDACs with high expression of *CDK1/2/5/9*; on the other hand, due to the lack of data on the individuals with low *CDK1/2/5/9* expression, experiments are still needed to verify the feasibility of using dinaciclib as a therapeutic candidate.

CK2 gene aberrations were detected in 2.9% (34/1228: *CSNK2A1*, 1%, *CSNK2A2*, 0.3%, *CDNK2B*, 1.6%) of PDAC patients, and only 0.2% (3/1228) involved protein structural changes, while the majority involved gene amplification [43]. Similar to silmitasertib target genes, dinaciclib target gene aberrations were present in 4.3% (46/1228: *CDK1*, 0.3%, *CDK2*, 1.1%, *CDK5*, 2%, *CDK9*, 1%) of PDAC patients, and only 0.5% (6/1228) involved protein structural changes, while the majority involved gene amplification [43]. Therefore, our study provides some reference value for the strategy of silmitasertib and dinaciclib in the treatment of PDAC.

We identified three different amino acid substitution variants of *KRAS* in nine of ten PDAC cell lines including *KRAS* p.Gly12Asp (c.35G>A), *KRAS* p.Gly12Val (c.35G>T), and *KRAS* p.Gln61His (c.183A>C). It was reported that patients with *KRAS* mutations showed a weak response to first-line gemcitabine-based therapy and had a poor prognosis [35]. In our study, significant differences in sensitivity to dinaciclib could not be observed between cell lines harboring the *KRAS* c.35G point mutation and wild-type cell lines, suggesting the inhibitory effect of dinaciclib is not affected by the *KRAS* c.35G point mutation. Interestingly, T3M4 cells, which carry a *KRAS* c.183A>C variant are more sensitive to dinaciclib than wild-type BxPc-3 cells, suggesting that dinaciclib may improve the efficacy of patients with specific *KRAS* c.183A>C mutation, but due to the limited number of cell lines, further experiments are still needed to verify the relationship between this *KRAS* mutation and the efficacy of dinaciclib. However, a comprehensive analysis of silmitasertib efficacy and *KRAS* mutations suggests that carrying the *KRAS* variants reduced the PDAC sensitivity to silmitasertib. Since AKT is an important effector kinase of CK2, inhibition of CK2 causes a reduced activation of AKT, whereas mutant KRAS directly activates the PI3K/AKT pathway [8,44]. This antagonism results in reduced sensitivity of *KRAS*-mutated cell lines to ssilmitasertib. Overall, blocking CDKs with dinaciclib in monotherapy may be beneficial to patients with the specific *KRAS* c.183A>C mutation, whereas silmitasertib monotherapy in patients with *KRAS* point mutations may not be a good option.

We identified that all tested PDAC cell lines contained at least one *TP53* mutation that causes amino acids to change. Our sequencing data revealed that the expression of *TP53* with fs mutations were lower than those of *TP53* with point mutations. Fs variants resulted in a strong disruption of *TP53* function, and low *TP53* mRNA expression was associated with a poor prognosis in PDAC patients [45,46]. On the other hand, the expressions of all missense variants of *TP53* in PDAC cell lines was higher than in the control, and it has been reported that some specific point mutations inactivate *TP53* (p.Arg175, p.Gly245, p. Arg248, p.Arg249, p.Arg273, and p.Arg282) and confer an advantage in tumor growth [47,48]. The same mechanism possibly also exists in the *TP53* p.Ala159Val, p.Tyr220Cys, and p.Gly360Val variants, which demonstrated similar expression properties. In addition, combined with the results of the PDAC inhibitory assays, cell lines carrying specific *TP53* variants (c.267delC, c.818G>A, and c.844C>T) were less sensitive to silmitasertib. These cell lines were all in the low (PaTu8988S, Panc-1, PaTu8988T, PaTu8902, and Colo357) sensitivity group. Knockdown of CK2 causes the enhanced transactivation of p53, thereby increasing apoptosis [49]. However, due to the inactivation caused by mutations in *TP53*, inhibition of CK2 did not transactivate these proteins. This may be the reason for the reduced efficacy of silmitasertib in cell lines with specific mutations in *TP53*. AsPc-1 and Colo357, which carry the fs variant, are both in the dinaciclib high sensitivity group, suggesting that dinaciclib may be able to improve the poor prognosis of the *TP53* fs mutation. However, the expression level of *TP53* cannot fully explain the observed responses of all cell lines to silmitasertib or dinaciclib. These results indicate that *TP53* variants are an indicator of an inhibitory response, while the expression level of *TP53* is not. Furthermore, the results of the PDAC inhibitory assays indicate that patients with *TP53* mutations may benefit from a potential application of dinaciclib and silmitasertib.

Our study focused on univariate genetic variants and did not evaluate the potential effect of complex variant landscapes. Thus, our conclusions are limited to direct genetic variants observed in the respective target genes of the evaluated inhibitors. Interactions between different gene aberrations, influence on downstream signaling as well as expression deregulations can also have significant influence. Accordingly, a bioinformatical complex analysis allowing drug target, target downstream signaling as well as bioinformatical modeling is needed. Furthermore, the complex validation of predicted mechanistic targets on cell biological level should be performed in the future to further evaluate factors influencing drug response.

## 4. Materials and Methods

### 4.1. Kinase Inhibitors

Kinase inhibitors, silmitasertib (CK2 inhibitor) and dinaciclib (CDK1/2/5/9 inhibitor) were purchased from Selleck Chemicals (Absource Diagnostics GmbH, Munich, Germany). According to the manufacturer’s instructions, silmitasertib and dinaciclib were separately dissolved in dimethyl sulfoxide (DMSO) (Sigma Aldrich Chemie GmbH, Steinheim, Germany) as a stock solution at a final concentration of 10 mM. The stock solutions were stored at −80 °C and diluted into corresponding working concentrations before each experiment.

### 4.2. Cell Lines and Cell Culture

PDAC cell lines AsPc-1, BxPc-3, Capan-1, Colo357, Panc-1, PaTu8902, PaTu8988T, PaTu8988S, SU.86.86, and T3M4 were kindly provided by the University of Greifswald. AsPc-1, BxPc-3, Colo357, Panc-1, SU.86.86, and T3M4 were cultured in RPMI1640 medium (PAN-Biotech, Aidenbach, Germany) supplemented with 10% heat-inactivated fetal calf serum (FCS) (PAN-Biotech) and 1% penicillin-streptomycin solution (10,000 U/mL Penicillin, 10 mg/mL Streptomycin) (PAN-Biotech). PaTu8902, PaTu8988T, PaTu8988S were cultured in DMEM/F12 medium (PAN-Biotech) supplemented with 10% heated-inactivated FCS and 1% penicillin-streptomycin solution. Capan-1 was cultured in RPMI1640 medium supplemented with 15% heat-inactivated FCS and 1% penicillin-streptomycin solution. After verifying that all cell lines were not contaminated by mycoplasma, these PDAC cell lines were maintained in a 5% CO_2_ incubator at 37 °C with a humidified atmosphere.

For all assays, the PDAC cell lines were seeded at the density of 3.3 × 10^4^ cells per milliliter in a 6-well plate (totally 4.5 mL per well), 24-well plate (totally 1.5 mL per well), or 96-well plate (totally 150 μL per well). For viability assays, after 24 h, the supernatant was discarded and media containing increasing concentrations (range from 1–10 μM for silmitasertib and 0.001–1 μM for dinaciclib) of inhibitors or vehicle (DMSO, as the control) were added to the corresponding PDAC cell lines. For the apoptosis/necrosis analysis, the inhibitor concentrations were selected according to the results of the cell viability assays. The inhibitor concentrations were adjusted according to the response observed in the viability assays for further analysis of the induced apoptotic and necrotic events. The treated cells were incubated for 72 h at 37 °C with 5% CO_2_. At the indicated time points, cell proliferation, metabolic activity, cell biomass, or apoptosis/necrosis was evaluated in at least three biologically independent replicates.

### 4.3. Cell Viability Assays

#### 4.3.1. Proliferation

Cell proliferation was evaluated by absolute counting and Trypan blue (Sigma-Aldrich Chemie GmbH, Steinheim, Germany) staining. After drug exposure in 24-well plates, the cells were harvested and washed by 1× PBS (PAN-Biotech). Following the cells being stained with Trypan blue, the number of viable cells was determined by counting with a hemocytometer. Proliferation was expressed as a percentage of viable cells treated with the inhibitor to the vehicle-treated control (control = 100%).

#### 4.3.2. Metabolic Activity

Metabolic activity was tested by using the Water Soluble Tetrazolium—1 (WST-1) assay (TaKaRa Bio Inc., Kusatsu, Japan). After exposure to the corresponding inhibitor, the cells were incubated with 15 μL WST-1 for 2 h in 96-well plates. Absorbances at 450 nm and the reference wavelength of 620 nm were measured by Promega GloMax^®^-Multi Microplate Multimode Reader (Promega, Madison, WI, USA) and the metabolic activity was calculated as recommended by the manufacturer. Metabolic activity was expressed as a percentage of the inhibitor-treated group to the vehicle-treated controls (control = 100%).

#### 4.3.3. Biomass Quantification

Biomass quantification was carried out by Crystal Violet (CV) (Sigma-Aldrich Chemie GmbH) staining. After exposure to the corresponding inhibitors in 96-well plates, the cells were washed once with PBS and stained with 50 μL 0.2% CV solution on a shaker at room temperature for 10 min. Following this, the plates were washed twice with PBS. To elute bound CV, 100 μL 1% sodium dodecyl sulfate (SDS) (SERVA Electrophoresis GmbH, Heidelberg, Germany) was added to each well and incubated on a shaker at room temperature for 10 min. Finally, absorbances at 570 nm and reference wavelength at 620 nm were measured by a Promega GloMax^®^-Multi Microplate Multimode Reader for background normalization. CV cell biomass estimation result was expressed as a percentage of the inhibitor-treated group to vehicle-treated controls (control = 100%).

### 4.4. Identification of IC_50_

IC_50_ values were calculated based on cell proliferation, metabolic activity, and biomass after 72 h of inhibitor exposure. GraphPad Prism Version 8.0.2 (GraphPad Software Inc., San Diego, CA, USA) was used to evaluate IC_50_. Briefly, after transforming concentrations and normalizing the results of the three vitality assays, nonlinear regression model (dose-response-inhibition vs. normalized response–variable slope) was used to evaluate the IC_50_ values. Calculate the IC_50_ corresponding to the three vitality assays, and apply these results to the response-based clustering analysis in order to evaluate the sensitivity of cell lines to inhibitors.

### 4.5. Apoptosis and Necrosis Analyses

Apoptosis and necrosis were evaluated by YO-PRO-1 (Invitrogen, Darmstadt, Germany) and propidium iodide (PI) (Sigma-Aldrich Chemie GmbH) double staining by flow cytometry. After exposure to the corresponding inhibitor, supernatants were collected and cells were harvested and washed twice with cold PBS. Following this, cells were resuspended in 200 μL YO-PRO-1 (final concentration: 0.2 μM) solution. After incubating at room temperature for 20 min in the dark, cells were washed twice in cold PBS and resuspended in 400 μL cold PBS. Then, cells were stained with PI (final concentration: 20 μg/mL) straightway before measurement. Unstained and single-stained cells were used as controls and measured in every single experiment. YO-PRO-1^−^/PI^−^ cells are considered to be viable cells, YO-PRO-1^+^/PI^−^ cells are considered to be apoptotic cells, and PI^+^ cells are considered to be necrotic cells. Flow cytometry measurement was performed on FACSverse (Becton, Dickinson and Company (BD), Heidelberg, Germany) and all data were analyzed by BD FlowJo software (BD).

### 4.6. Nucleic Acid Extraction

Genomic DNA was extracted by the NucleoSpin^®^ Tissue Kit (MACHEREY-NAGEL GmbH, Dueren, Germany) according to the manufacturers’ instructions. In brief, 5 × 10^6^ cells were harvested from each continuous cultural cell line and washed twice with cold sterile PBS. Cell pellets were lysed, then the lysis that contained genomic DNA were extracted and purified by a silica membrane of the NucleoSpin column. Finally, genomic DNA was eluted by 30 μL of nuclease-free water.

Total RNAs were extracted by miRNeasy Mini Kit (QIAGEN GmbH, Hilden, Germany) according to the manufacturers’ instructions. In brief, 5 × 10^6^ cells were harvested from each continuous cultural cell line and washed twice with cold sterile PBS. Cell pellets were resuspended in 700 μL QIAzol Lysis Reagent (QIAGEN GmbH), then the aqueous phase that contained the total RNA of the lysed cells were extracted and purified by a silica membrane of RNeasy Mini spin columns. Finally, total RNA was eluted by 30 μL of nuclease-free water.

After extraction, nucleic acid concentrations as well as OD 260/280 and OD 260/230 ratios were measured with a NanoDrop 1000 Spectrophotometer (Thermo Fisher Scientific Inc., Waltham, MA, USA).

### 4.7. Whole Exome Sequencing

Barcoded sequencing libraries were generated after enrichment with the SureSelect Human All Exon Kit (Agilent, Santa Clara, CA, USA), pooled and sequenced on a HiSeq4000 (Illumina Inc., San Diego, CA, USA) instrument using 150 paired-end protocol to yield at least 20× coverage for >98% of the target region and an overall average depth of coverage above 100×. An in-house bioinformatics pipeline including read alignment to human genome reference hg 19, variant calling (single nucleotide substitutions and small deletions/insertions), and variant annotation with publicly available data based was used.

### 4.8. Variant Calling Filtering Strategy

**Figure 7:**
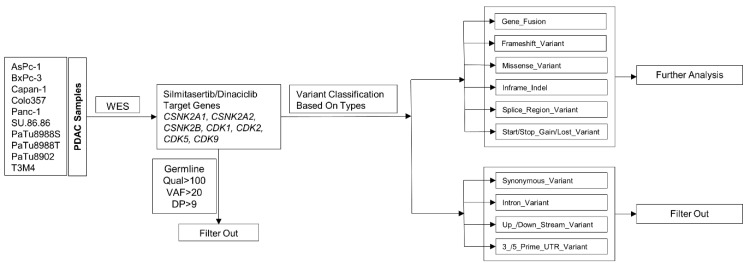
Filtering strategy of inhibitor target genes.

After WES, the sequencing data from ten PDAC cell lines were obtained and filtered in order to select variants with the expected highest impact on gene function. Briefly, variants were filtered based on quality (qual), VAF, depth of coverage (DP), and variant type. In order to exclude false positive variants, only variants with qual > 100, VAF > 20, and DP > 9 were included in our analysis. Germline mutations were excluded through a comparison with the COSMIC and dbSNP databases. Then, variant types were excluded that were not able to cause amino acid substitution, RNA structure change, or base insertions/deletions (indels). These variant types include synonymous variants, intronic variants, upstream or downstream variants, 3 prime or 5 prime UTR variants. After this filtering procedure, missense variants, splice region variants, inframe indels, frameshift variants, gene fusion, start/stop gain, or lost were kept for further analysis (Figure 7).

### 4.9. Gene Expression Analyses

Barcoded sequencing libraries were prepared with the TruSeq Stranded mRNA Kit (Illumina), pooled, and sequenced on a NextSeq 500 System (Illumina) using the 75 bp paired-end protocol. At least 30 million reads were obtained for each sample. The reads were aligned to reference genome GRCh37/Release 38 with STAR V.2.7.6a using the two-pass mode [50]. Transcript abundance estimates were calculated by counting the reads using featureCounts/subread V.2.0.1 [51].

The expression data of non-neoplastic pancreatic tissue from The Genotype-Tissue Expression (GTEx) and The Cancer Genome Atlas Program (TCGA) were chosen as the control. Non-inhibitor target genes were analyzed to exclude the tumor-induced upregulation of all genes.

### 4.10. Response-Based Clustering Strategy

The cell sensitivity grouping was performed by the k-means++ clustering method based on an unsupervised machine learning algorithm. Briefly, after performing viability assays on all ten PDAC cell lines, we obtained the IC_50_ values of cell proliferation and biomass. Then, these IC_50_ results were applied to the Sci-kit learn package using Python programming language to predict optimal clusters. The Silhouette score was used to detect the clustering density and the separation between clusters [52]. Ten cell lines were set to be divided into several clusters, and the cluster grouping was iterated a maximum of 100 times to test for the robustness of the classification. Finally, the ten cell lines were divided into different clusters, and identified as high, moderate, and low sensitivity groups based on their biological characteristics.

### 4.11. Statistical Analyses

Data were replicated with at least three biologically independent experiments. Results of proliferation, metabolic activity, biomass quantification, and apoptosis/necrosis analysis were expressed as mean ±  standard deviation (SD). Statistical significance was determined by one-way ANOVA (after proving the data within each group conformed to the Gaussian distribution) or Kruskal–Wallis test (the data within each group conformed to non-Gaussian distribution) and displayed as * *p* < 0.033, ** *p* < 0.002, *** *p* < 0.001 versus the control group.

## 5. Conclusions

Our present study revealed distinct sensitivities of the PDAC cell lines when treated with dinaciclib or silmitasertib. Neither the expression level of the inhibitor target genes nor gene variants could affect the differences in the observed sensitivity to these drugs. For PDAC hotspot genes, the *KRAS* variants may reduce the sensitivity of PDAC cell lines to silmitasertib. Specific *TP53* variants including c.267delC, c.818G>A, and c.844C>T, reduced the sensitivity of silmitasertib to the PDAC cell lines. Interestingly, cell lines carrying *TP53* frameshift variants are highly sensitive to dinaciclib compared to cell lines carrying *TP53* point mutations. Thus, both inhibitors displayed excellent in vitro efficacy on PDAC cell lines, and further experiments are still needed to verify the in vivo efficacy and the effects of the target genes and hotspot genes on the efficacy of the inhibitors.

## Data Availability

The data supporting the reported results can be found on the website in detail in the article.

## Supplementary Materials

The following supporting information can be downloaded at: https://www.mdpi.com/article/10.3390/ijms23084409/s1.

## Abbreviations

AKT, *AKT*	Protein kinase B
Ala	Alanine
Arg	Arginine
Asp	Aspartate
CDK, *CDK*	Cyclin-dependent kinase
CK2, *CSNK2*	Casein kinase II
CV	Crystal violet
Cys	Cysteine
DMSO	Dimethyl sulfoxide
DP	Depth of coverage
DRB	5,6-Dichloro-1-ß-D-ribofuranosylbenzimidazole
ERK	Extracellular regulated kinase
FCS	Fetal calf serum
Fs	Frameshift
Gln	Glutamine
Gly	Glycine
GTEx	The genotype-tissue expression
His	Histidine
IC50	Half maximal inhibitory concentration
Indel	Insertion/deletion
JAK	Janus kinase
JNK	C-Jun N-terminal kinase
KRAS, *KRAS*	Kirsten’s rat sarcoma viral oncogene homolog
MEK	Mitogen-activated protein kinase kinase
MKK4	Dual-specificity mitogen-activated protein kinase kinase 4
OD	Optical density
PBS	Phosphate buffer saline
PDAC	Pancreatic ductal adenocarcinoma
PI	Propidium iodide
PI3K	Phosphoinositide 3-kinase
PIP3	Phosphatidylinositol 3,4,5-trisphosphate
PTEN	Phosphatase and tensin homolog
qual	Variant confidence
Rb	Retinoblastoma
RNA-seq	RNA sequencing
Ser	Serine
STAT	Signal transducer and activator of transcription
TCGA	The Cancer Genome Atlas Program
Thr	Threonine
P53, *TP53*	Tumor protein p53
TPM	Transcripts per kilobase million
UTR	Untranslated region
VAF	Variant allele frequency
Val	Valine
WES	Whole exome sequencing
WST-1	Water soluble tetrazolium-1
