# Supplementary material for: Inhibitory Response to CK II Inhibitor Silmitasertib and CDKs Inhibitor Dinaciclib Is Related to Genetic Differences in Pancreatic Ductal Adenocarcinoma Cell Lines
Source: Int J Mol Sci. 2022 Apr 16;23(8):4409. doi: 10.3390/ijms23084409 (PMC9031017; doi:10.3390/ijms23084409)
Supplement: Supplementary file 1 [file ijms-23-04409-s001.zip › Supplementary Figures.pdf]

Supplementary Figure S1

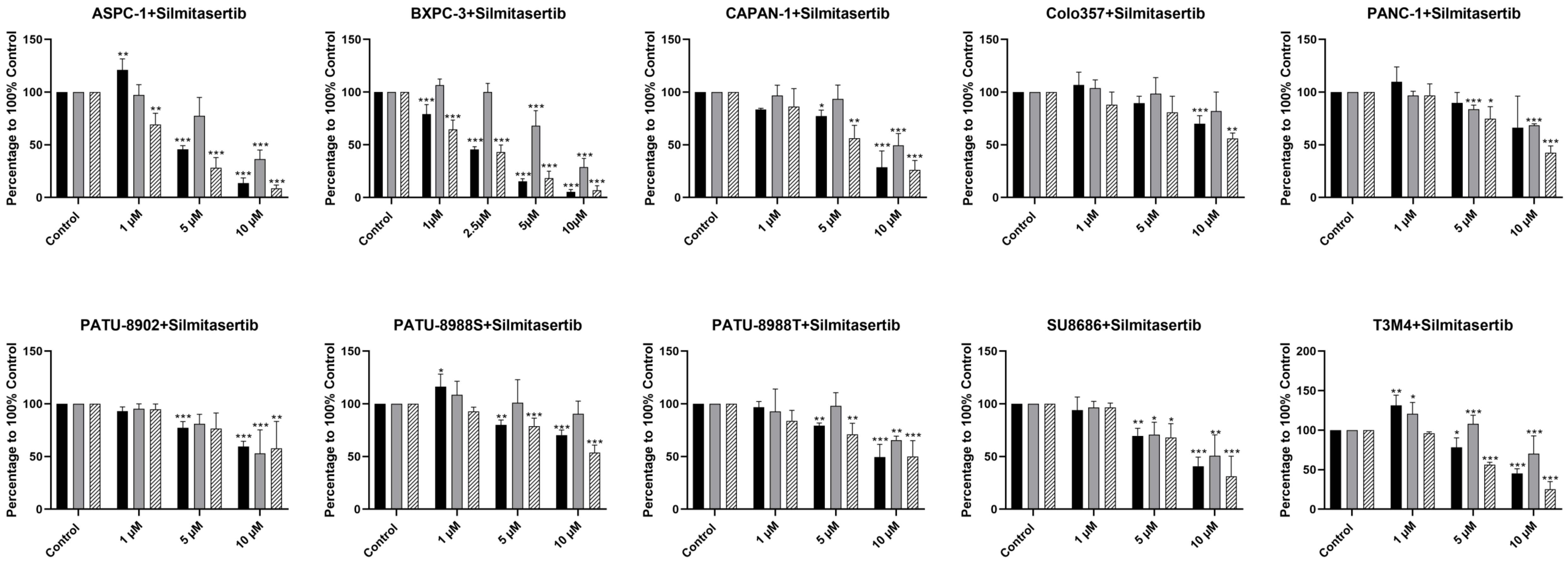

Supplementary Figure S1. Cell viability after 72h Silmitasertib exposure in ten PDAC cell lines. Data are presented as Mean ± SD. Significance of a treatment effect (which showed in bar charts) compared to the DMSO control was determined by one-way ANOVA or Kruskal-Wallis-Test and displayed as *: *P*< 0.033, **: *P*< 0.002, ***: *P*<0.001 (n ≥ 3).

Supplementary Figure S2

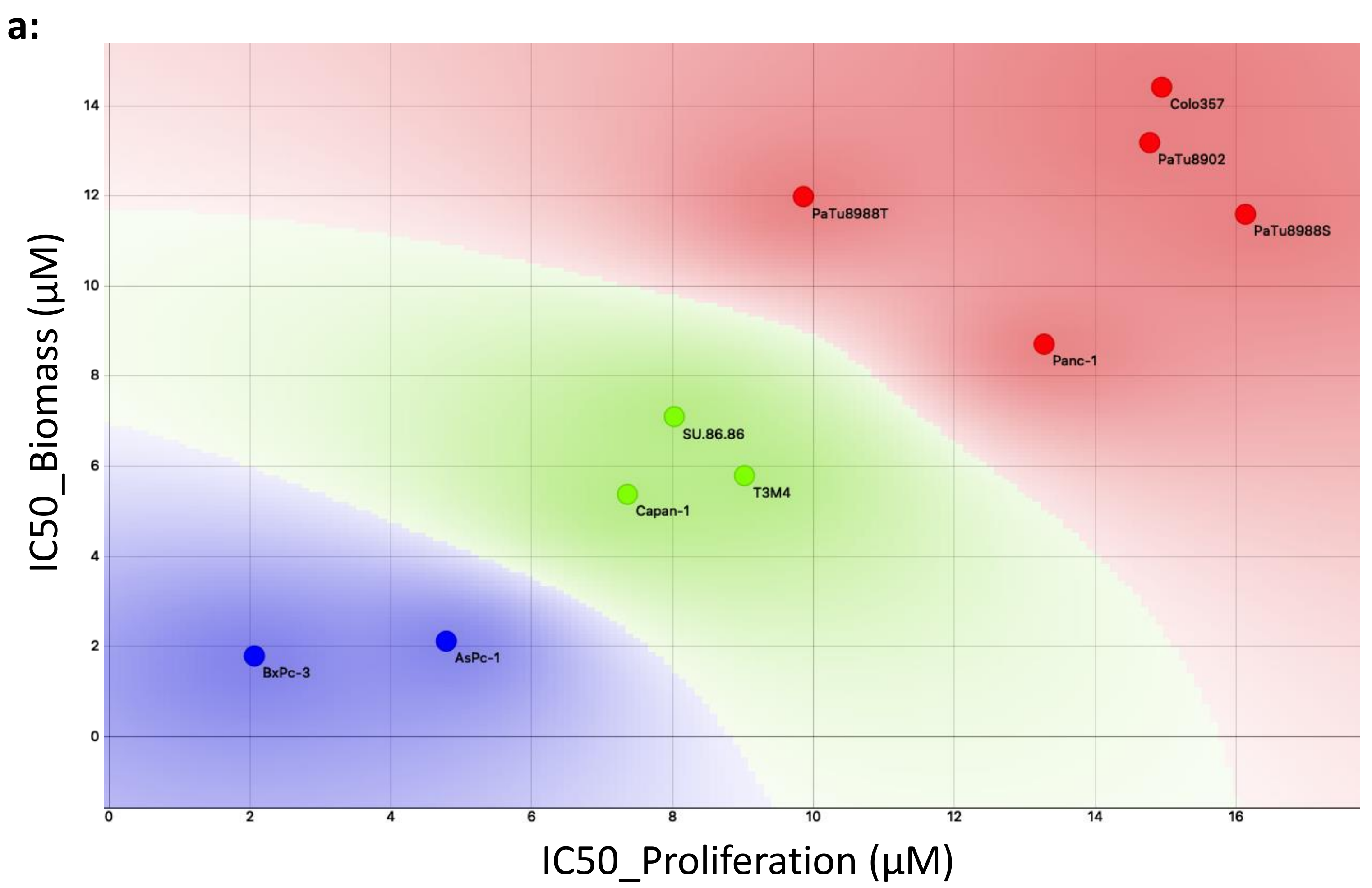

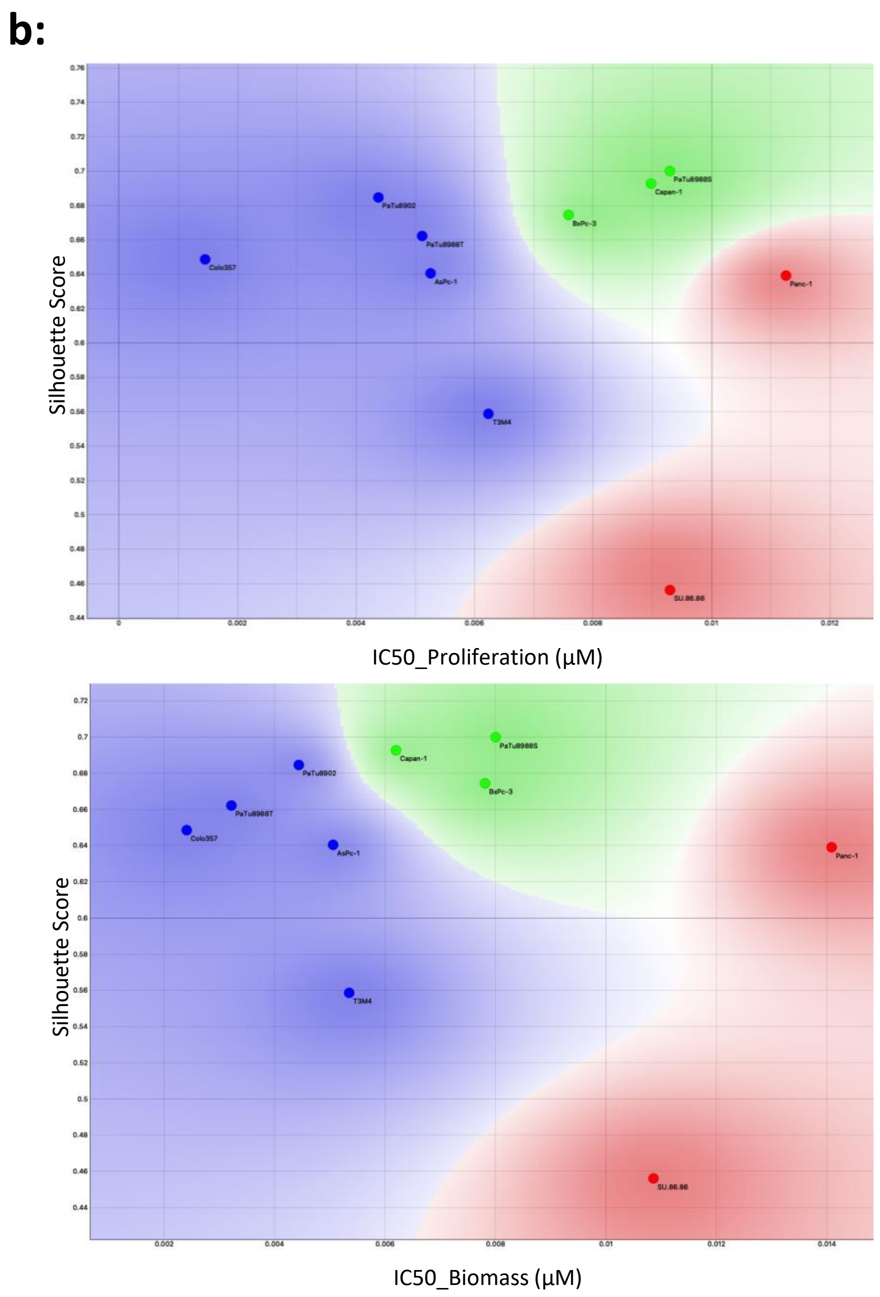

Supplementary Figure S2. Clustering **(a)** and Silhouette score **(b)** results based on IC50 of cell proliferation and biomass after 72 h of Silmitasertib exposure. K-means++ (an unsupervised machine learning algorithm) was used to classify cell line to low sensitive group (red), moderate sensitive group (green), and high sensitive group (blue). In figure (a), the X- and Y- axis represent IC50 values calculated from cell proliferation and biomass. In figure (b), from top to bottom are the results of cell proliferation and biomass, the X-axis represents IC50 calculated according to cell proliferation and biomass, and the Y-axis represents the Silhouette score generated by k-means++.

Supplementary Figure S3

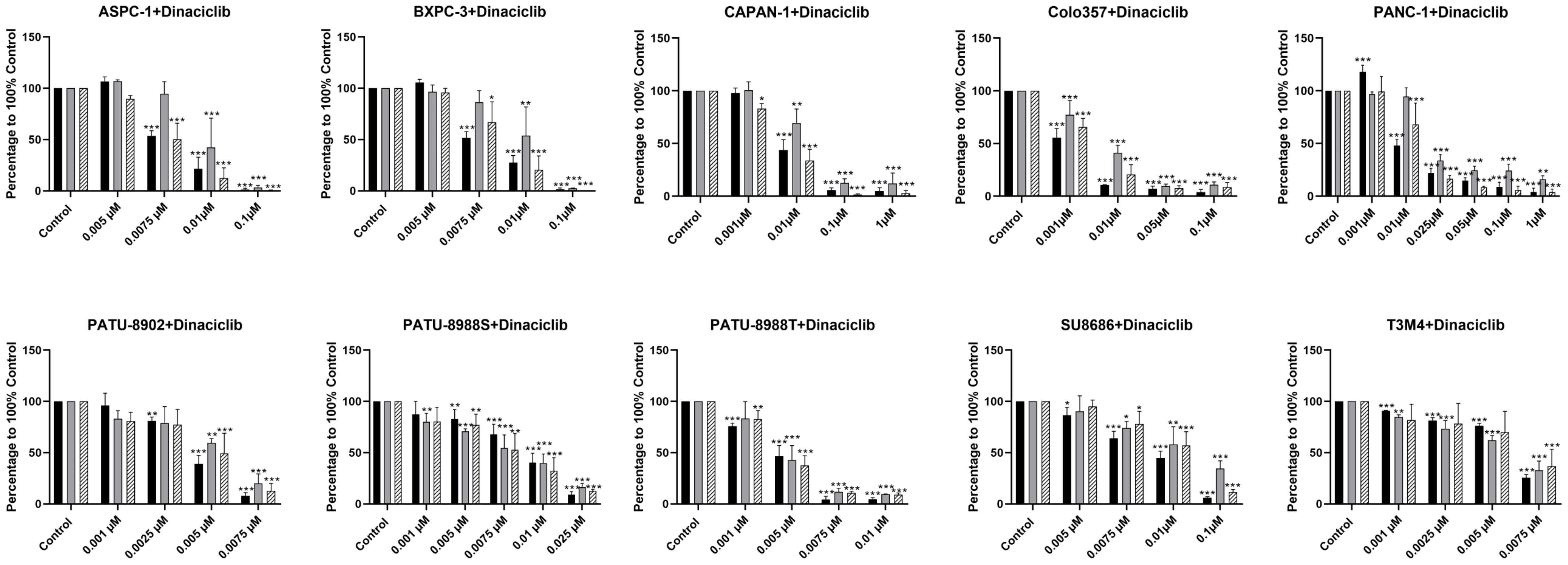

Supplementary Figure S3. Cell viability after 72h Dinaciclib exposure in ten PDAC cell lines. Data are presented as Mean ± SD. Significance of a treatment effect (which showed in bar charts) compared to the DMSO control was determined by one-way ANOVA or Kruskal-Wallis-Test and displayed as *: P< 0.033, **: P< 0.002, ***: P<0.001 (n ≥ 3).

Supplementary Figure S4

a:

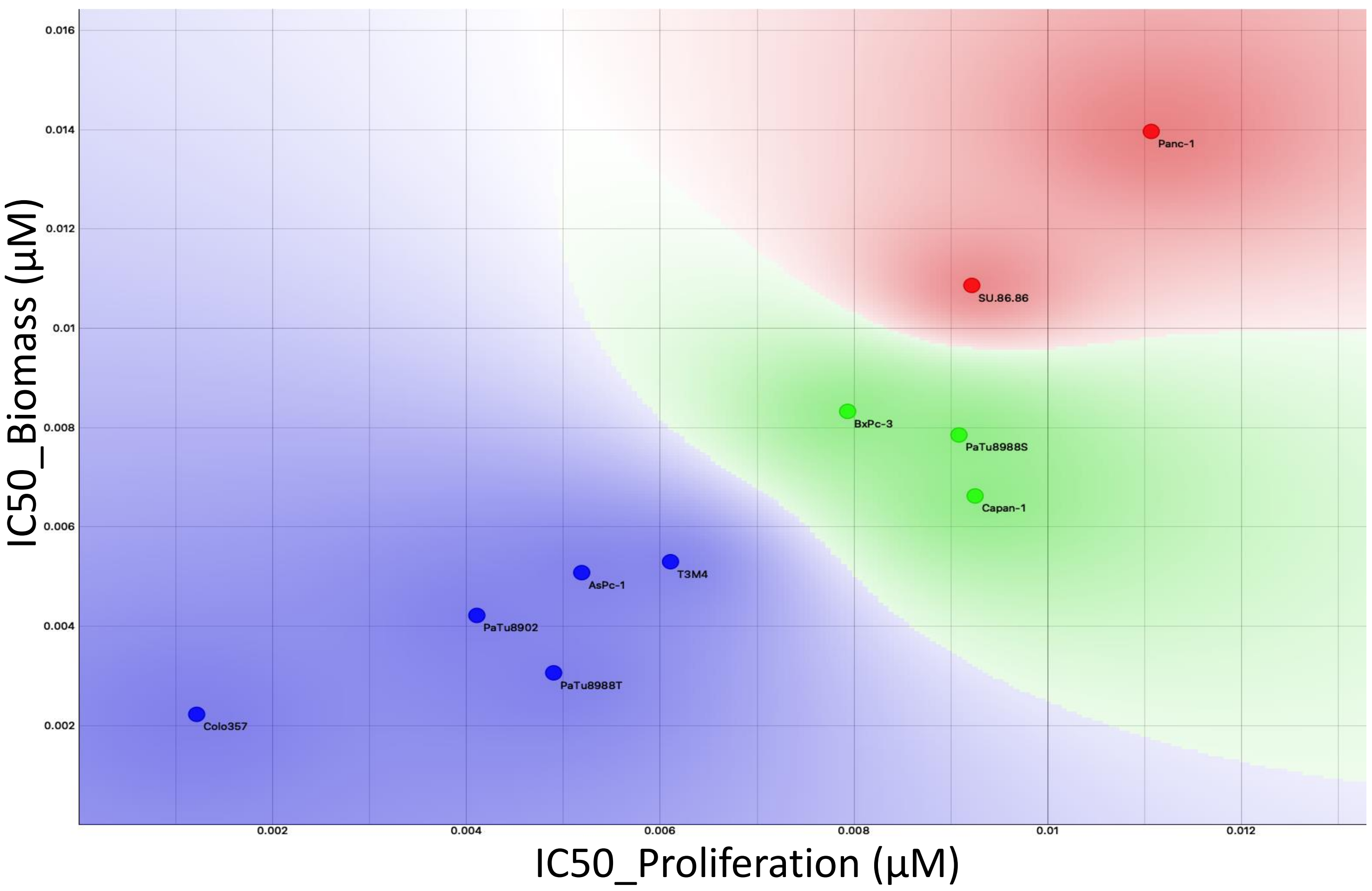

b:

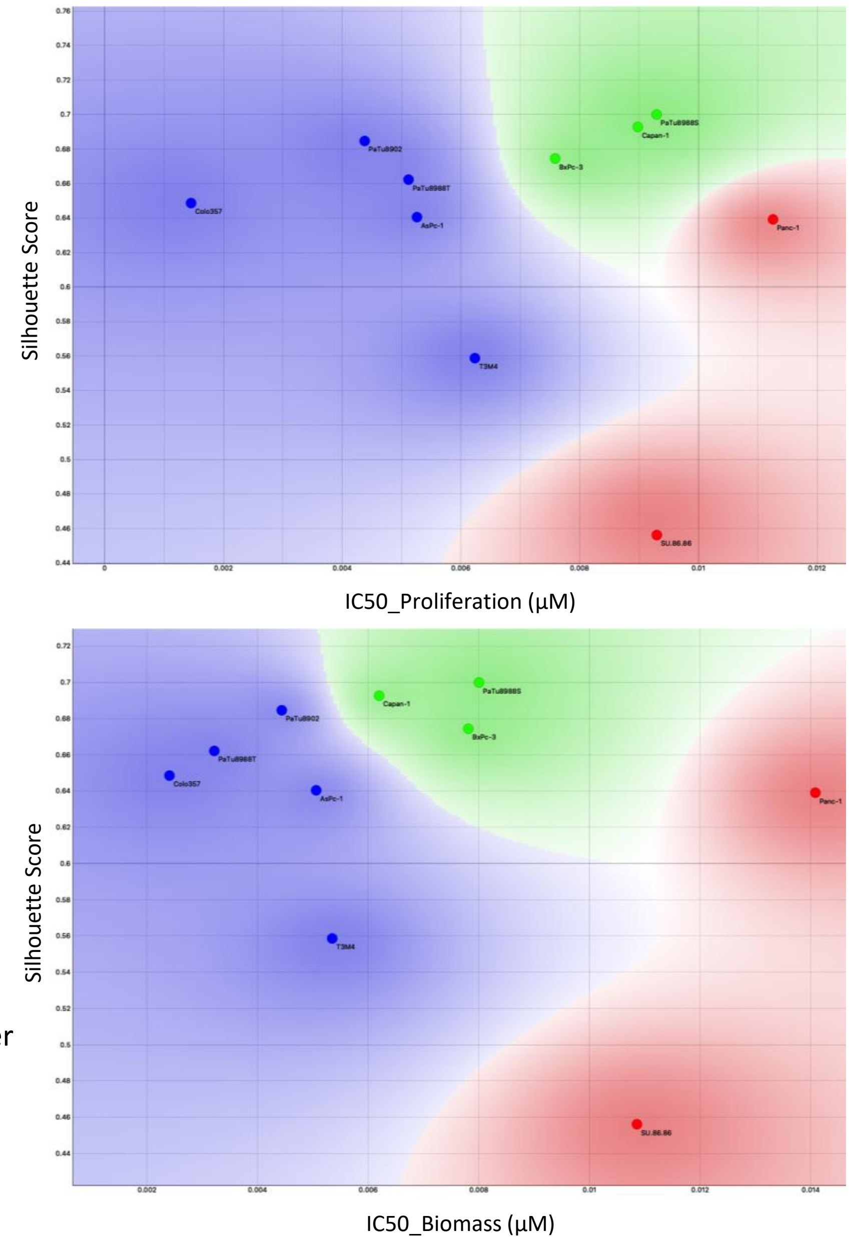

Supplementary Figure S4. Clustering **(a)** and Silhouette score **(b)** results based on IC50 of cell proliferation and biomass after 72 h of Dinaciclib exposure. K-means++ (an unsupervised machine learning algorithm) was used to classify cell line to low sensitive group (red), moderate sensitive group (green), and high sensitive group (blue). In figure (a), the X- and Y- axis represent IC50 values calculated from cell proliferation and biomass. In figure (b), from top to bottom are the results of cell proliferation and biomass, the X-axis represents IC50 calculated according to cell proliferation and biomass, and the Y-axis represents the Silhouette score generated by k-means++.

Supplementary Figure S5

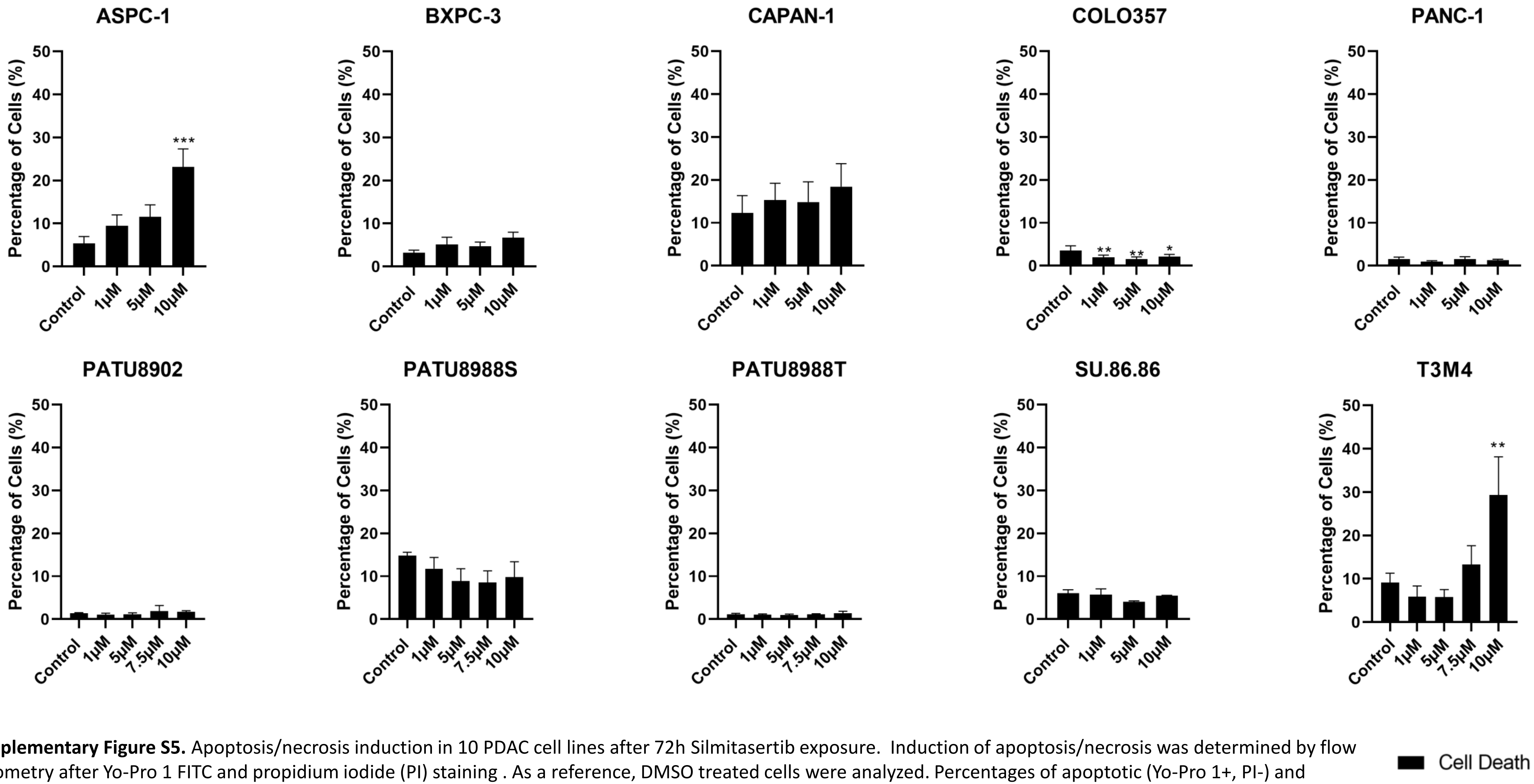

Supplementary Figure S5. Apoptosis/necrosis induction in 10 PDAC cell lines after 72h Silmitasertib exposure. Induction of apoptosis/necrosis was determined by flow cytometry after Yo-Pro 1 FITC and propidium iodide (PI) staining . As a reference, DMSO treated cells were analyzed. Percentages of apoptotic (Yo-Pro 1+, PI-) and necrotic (PI+) cells were determined and displayed as the Mean ± SD of at least three independent measurements. Significance of a treatment effect compared to the DMSO control was determined by one-way ANOVA or Kruskal-Wallis-Test and displayed as *: $P< 0.033$, **: $P< 0.002$, ***: $P<0.001$ ($n \geq 3$).

Supplementary Figure S6

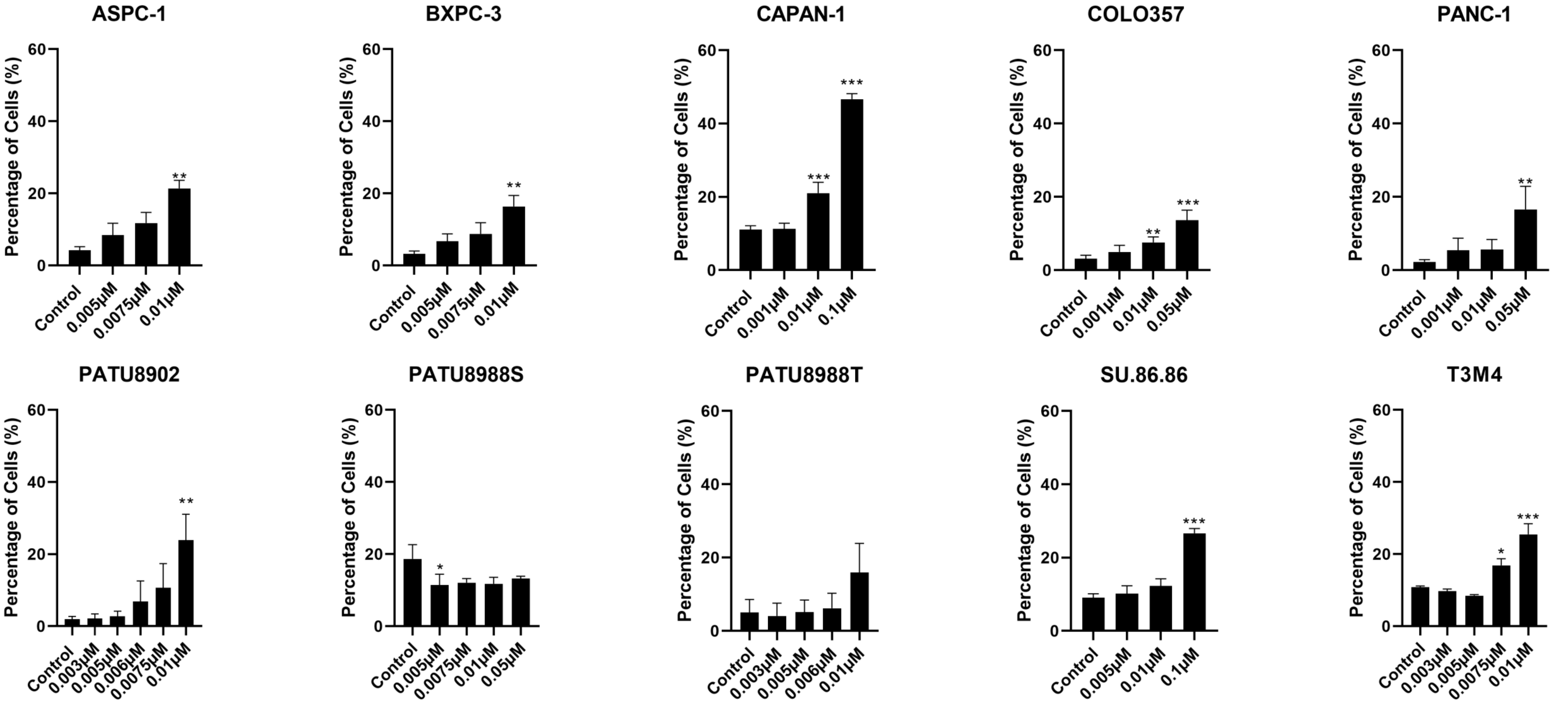

Supplementary Figure S6. Apoptosis/necrosis induction in 10 PDAC cell lines after 72h Dinaciclib exposure. Induction of apoptosis/necrosis was determined by flow cytometry after Yo-Pro 1 FITC and propidium iodide (PI) staining . As a reference, DMSO treated cells were analyzed. Percentages of apoptotic (Yo-Pro 1+, PI-) and necrotic (PI+) cells were determined and displayed as the Mean ± SD of at least three independent measurements. Significance of a treatment effect compared to the DMSO control was determined by one-way ANOVA or Kruskal-Wallis-Test and displayed as *: P< 0.033, **: P< 0.002, ***: P<0.001 (n ≥ 3).

Supplementary Figure S7 – Apoptosis/Necrosis Dot Plot – Silmitasertib (1)

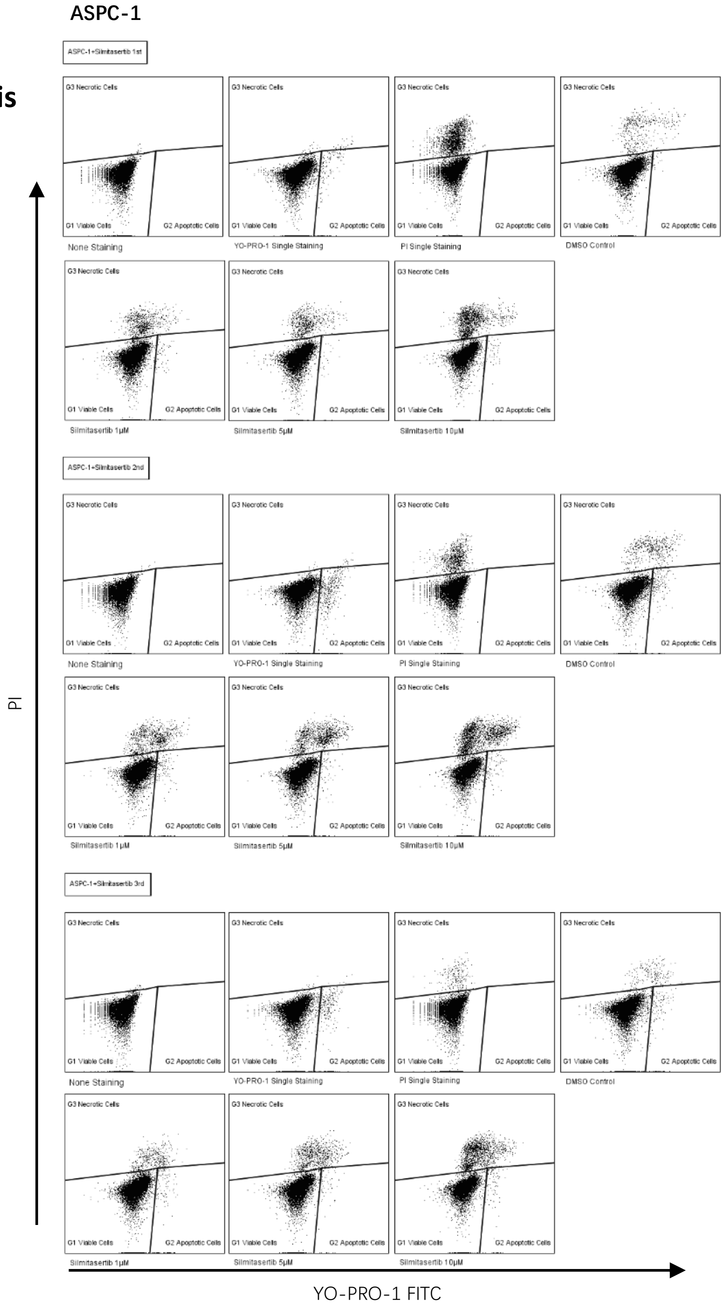

	1st		2nd		3rd		Mean (Cell Death)	SD	P-Value (vs. Control)
Concentration	G2(YP⁺/PI⁻)	G3(PI⁺)	G2(YP⁺/PI⁻)	G3(PI⁺)	G2(YP⁺/PI⁻)	G3(PI⁺)	G2+G3		G2+G3
Control	0.56	4.71	1.94	5.43	1.34	2.14	5.37	1.59	
1μM	0.91	9.95	1.62	10.0	1.42	4.47	9.46	2.54	0.42
5μM	0.55	8.22	0.94	14.5	0.88	9.45	11.51	2.85	0.16
10μM	0.66	18.5	1.31	27.7	1.11	20.1	23.13	4.24	<0.001

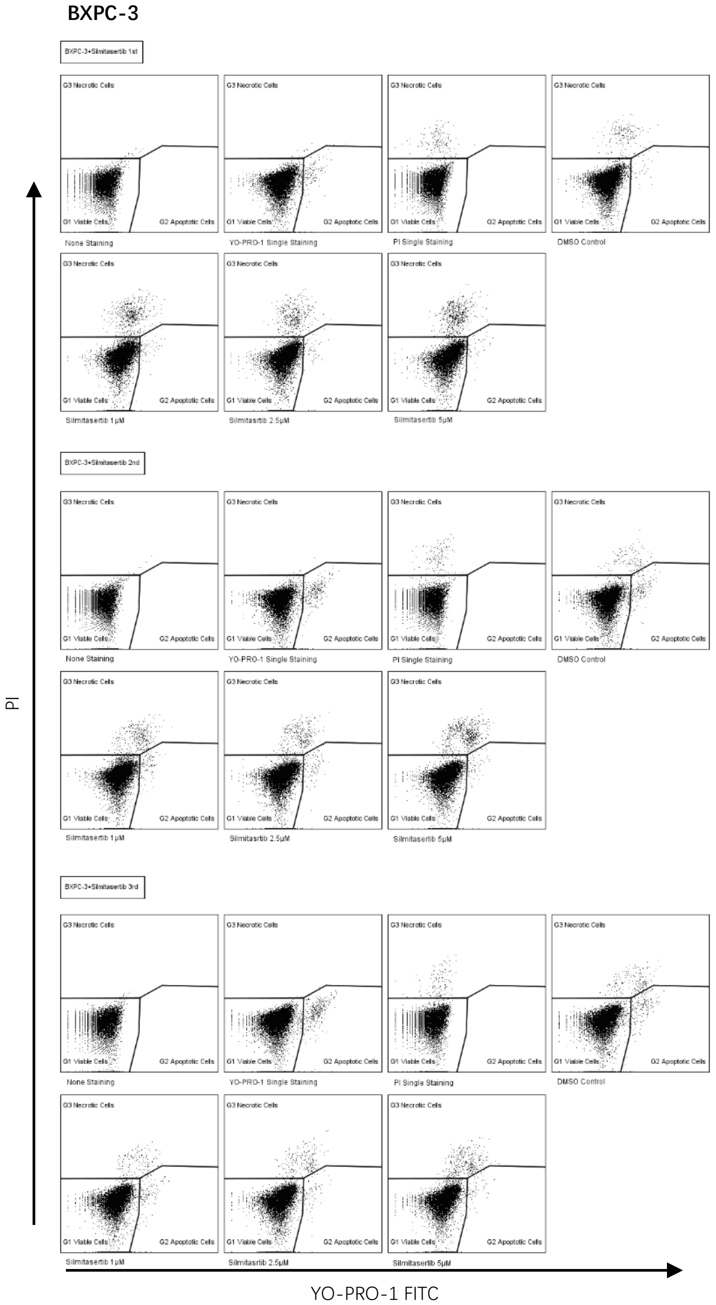

	1st		2nd		3rd		Mean (Cell Death)	SD	P-Value (vs. Control)
Concentration	G2(YP⁺/PI⁻)	G3(PI⁺)	G2(YP⁺/PI⁻)	G3(PI⁺)	G2(YP⁺/PI⁻)	G3(PI⁺)	G2+G3		G2+G3
Control	0.43	2.01	1.98	1.24	1.95	2.02	3.21	0.62	3.21
1μM	0.97	6.17	1.42	3.79	1.59	1.48	5.14	1.66	5.14
5μM	0.91	4.94	1.27	3.53	1.17	2.20	4.67	1.02	4.67
10μM	0.77	6.47	0.90	7.01	0.90	4.00	6.68	1.29	6.68

Supplementary Figure S7. Apoptosis/necrosis dot plot in 10 PDAC cell lines after 72h Silmitasertib exposure. The cell population strategy is based on the manufacturer's recommendation.
G1: Viable cells; G2: Apoptotic cells; G3: Necrotic cells.
Reference: https://www.thermofisher.com/ (accessed on 14. Oct. 2021)

Supplementary Figure S7 – Apoptosis/Necrosis Dot Plot – Silmitasertib (2)

CAPAN-1

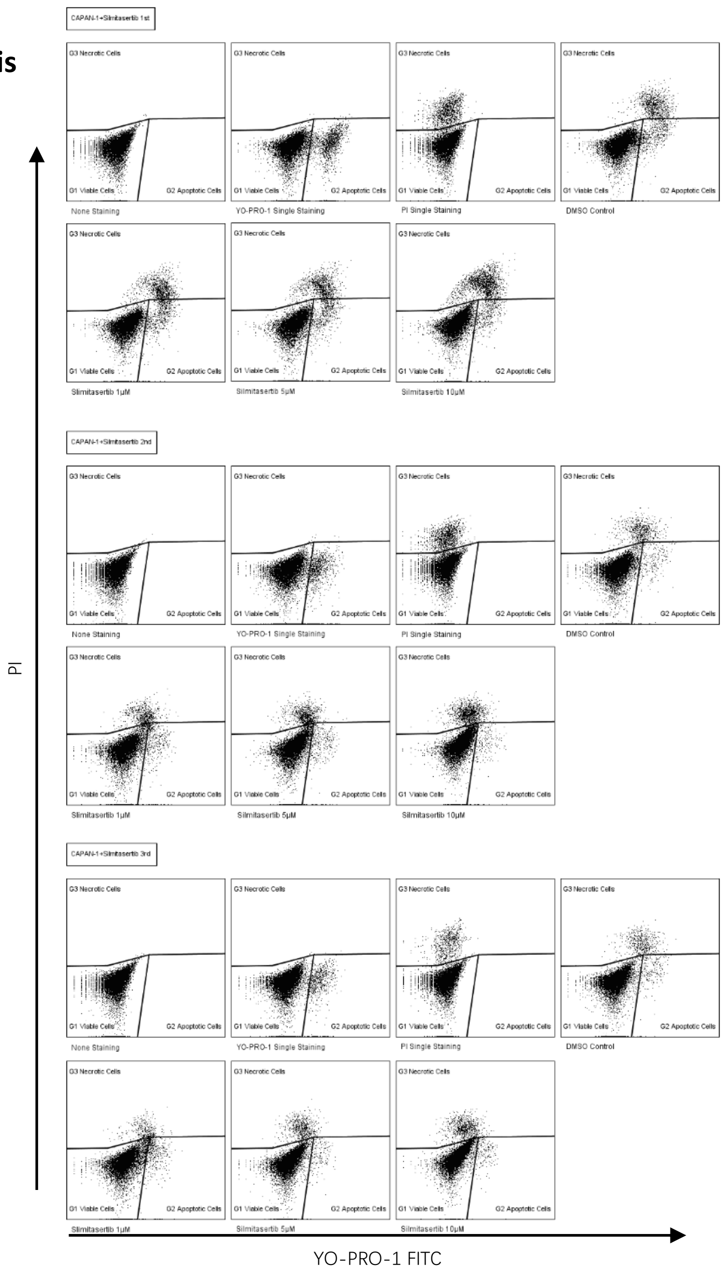

	1st		2nd		3rd		Mean (Cell Death)	SD	P-Value (vs. Control)
Concentration	G2(YP⁺/PI⁻)	G3(PI⁺)	G2(YP⁺/PI⁻)	G3(PI⁺)	G2(YP⁺/PI⁻)	G3(PI⁺)	G2+G3		G2+G3
Control	7.43	10.4	3.59	7.20	2.88	5.47	12.32	4.02	
1μM	8.75	11.9	6.70	7.18	7.30	4.14	15.32	3.90	0.85
5μM	8.84	11.9	5.55	9.10	2.25	6.88	14.84	4.74	0.9
10μM	5.47	19.7	3.68	14.4	2.76	9.30	18.44	5.36	0.44

Supplementary Figure S7 – Apoptosis/Necrosis Dot Plot – Silmitasertib (3)

COLO357

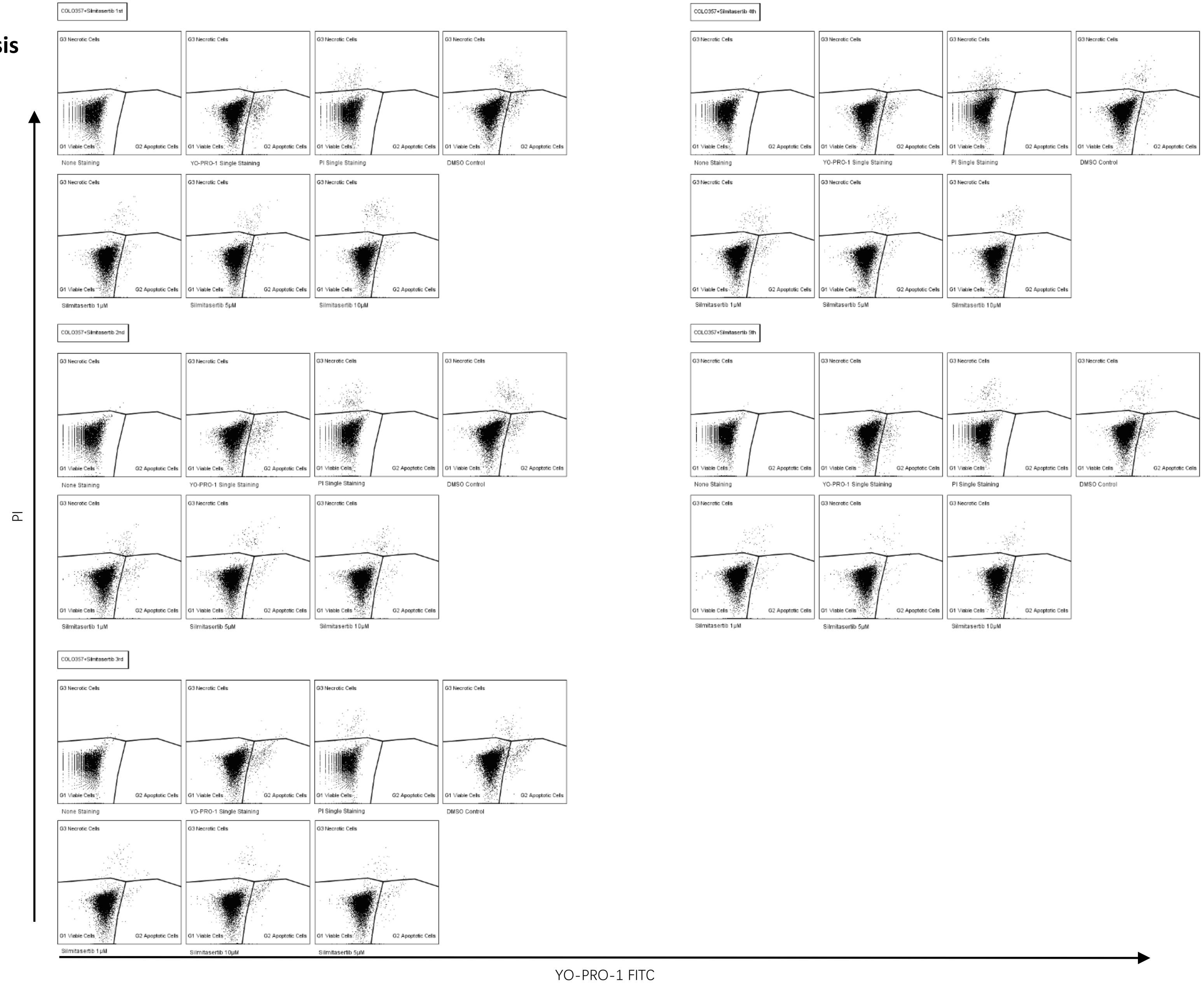

	1st		2nd		3rd		4th		5th		Mean (Cell Death)	SD	P-Value (vs. Control)
Concentration	$G2(YP^+/PI^-)$	$G3(PI^+)$	$G2(YP^+/PI^-)$	$G3(PI^+)$	$G2(YP^+/PI^-)$	$G3(PI^+)$	$G2(YP^+/PI^-)$	$G3(PI^+)$	$G2(YP^+/PI^-)$	$G3(PI^+)$	G2+G3		
Control	2.91	2.23	2.38	1.81	3.01	0.73	1.41	0.90	1.65	0.88	3.58	1.05	
1μM	1.21	0.68	1.72	1.09	0.71	0.60	1.31	0.94	0.80	0.56	1.92	0.56	0.01
5μM	1.41	0.83	1.10	0.83	1.13	0.48	0.59	0.63	0.52	0.38	1.58	0.48	0.002
10μM	1.32	1.40	0.86	0.97	1.89	0.61	0.53	0.82	1.67	0.69	2.15	0.50	0.03

Supplementary Figure S7 – Apoptosis/Necrosis Dot Plot – Silmitasertib (4)

PANC-1

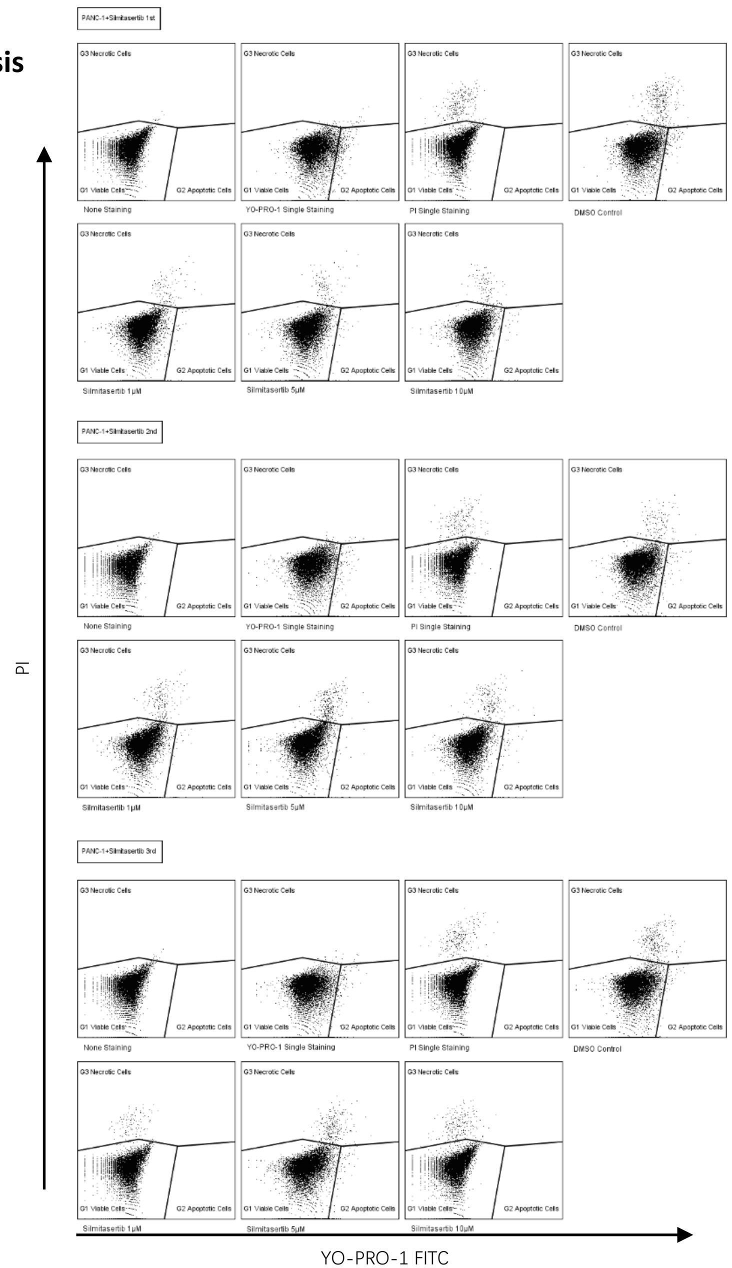

	1st		2nd		3rd		Mean (Cell Death)	SD	P-Value (vs. Control)
Concentration	G2(YP⁺/PI⁻)	G3(PI⁺)	G2(YP⁺/PI⁻)	G3(PI⁺)	G2(YP⁺/PI⁻)	G3(PI⁺)	G2+G3		G2+G3
Control	0.41	1.76	0.36	0.80	0.13	1.25	1.57	0.43	
1μM	0.097	0.70	0.12	1.19	0.00	0.75	0.95	0.25	0.32
5μM	0.17	0.66	0.16	1.93	0.24	1.55	1.57	0.54	>0.99
10μM	0.24	0.72	0.25	1.25	0.00	1.40	1.29	0.23	0.81

PATU8902

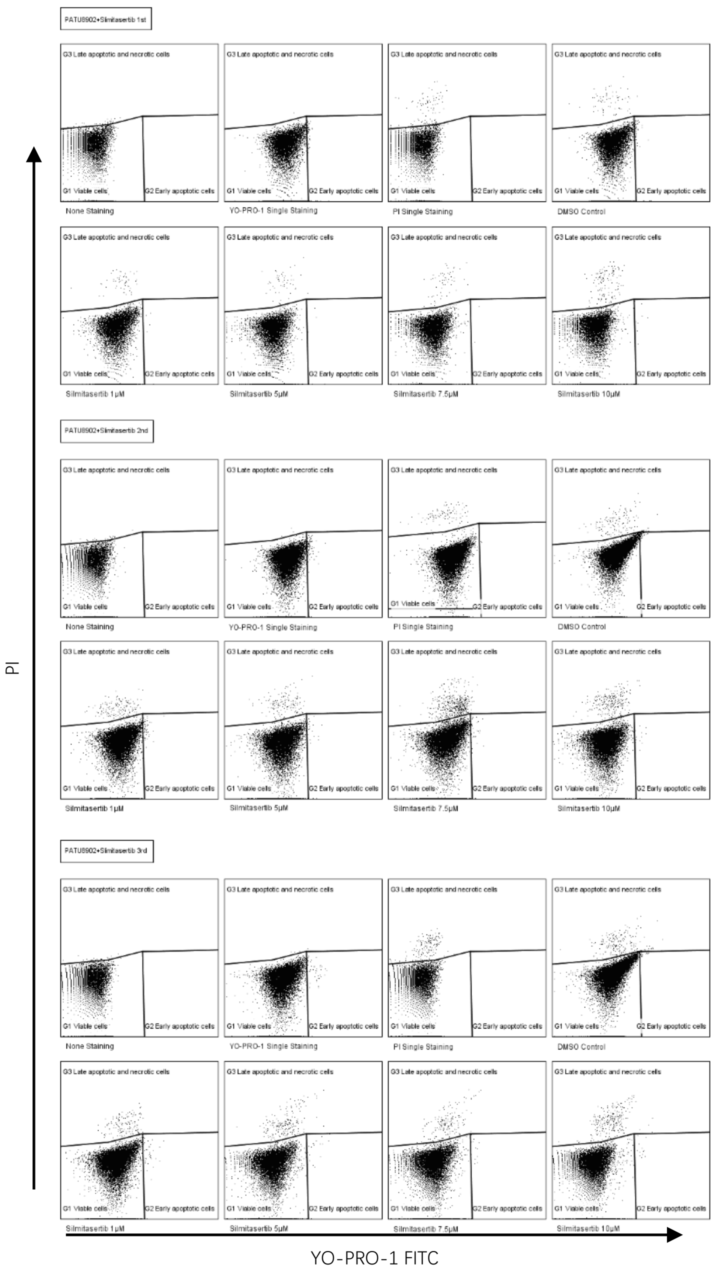

	1st		2nd		3rd		Mean (Cell Death)	SD	P-Value (vs. Control)
Concentration	G2(YP⁺/PI⁻)	G3(PI⁺)	G2(YP⁺/PI⁻)	G3(PI⁺)	G2(YP⁺/PI⁻)	G3(PI⁺)	G2+G3		G2+G3
Control	0.45	0.70	0.41	0.99	0.18	1.35	1.36	0.16	
1μM	0.051	0.60	0.31	1.04	0.25	0.99	1.08	0.31	0.98
5μM	0.046	0.57	0.14	1.02	0.12	1.41	1.10	0.38	0.98
7.5μM	0.041	0.62	0.21	3.49	0.082	1.15	1.86	1.32	0.85
10μM	0.056	1.26	0.10	1.86	0.089	1.78	1.71	0.28	0.95

Supplementary Figure S7 – Apoptosis/Necrosis Dot Plot – Silmitasertib (5)

PATU8988S

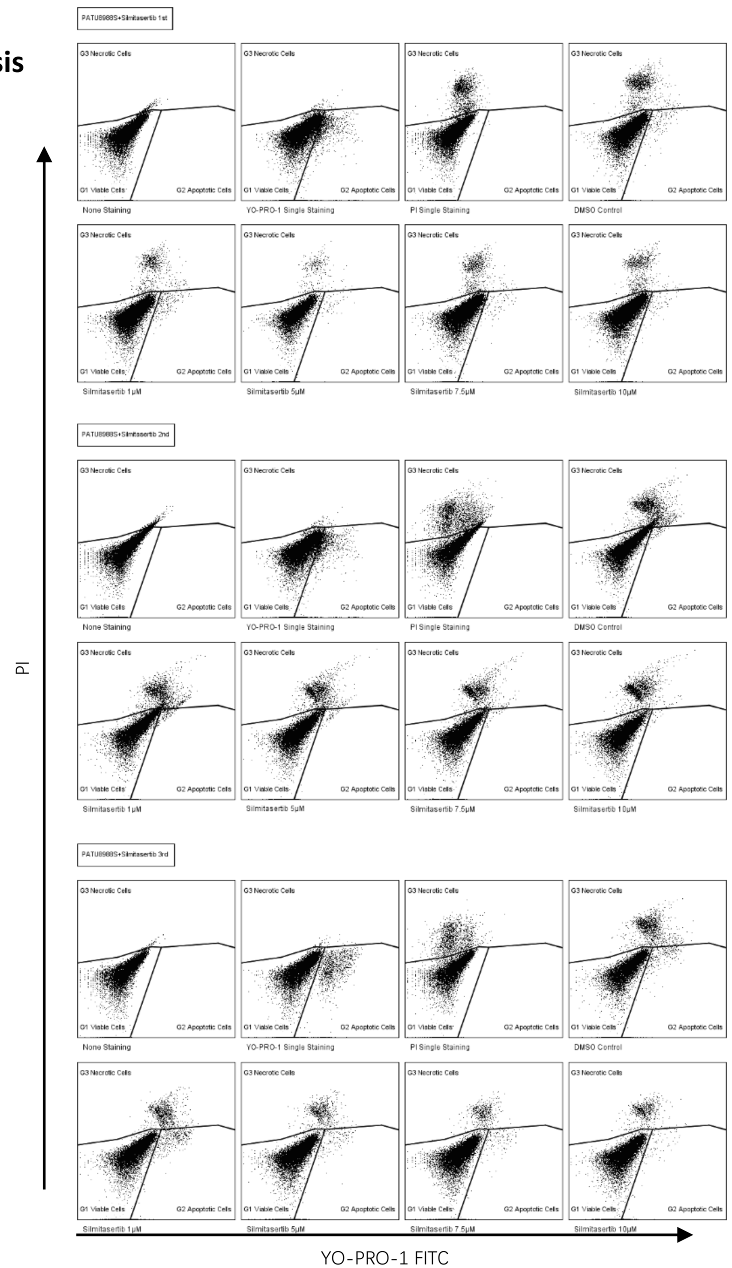

	1st		2nd		3rd		Mean (Cell Death)	SD	P-Value (vs. Control)
Concentration	G2(YP⁺/PI⁻)	G3(PI⁺)	G2(YP⁺/PI⁻)	G3(PI⁺)	G2(YP⁺/PI⁻)	G3(PI⁺)	G2+G3		G2+G3
Control	2.09	11.7	2.04	12.8	3.68	12.1	14.80	0.81	
1μM	1.93	6.24	2.87	11.3	4.69	8.30	11.78	2.60	>0.99
5μM	1.36	4.51	1.11	11.7	1.87	6.13	8.89	2.90	0.18
7.5μM	1.18	6.31	0.59	11.7	1.41	4.37	8.52	2.76	0.09
10μM	0.91	6.38	0.40	14.5	1.19	6.07	9.82	3.59	0.40

PATU8988T

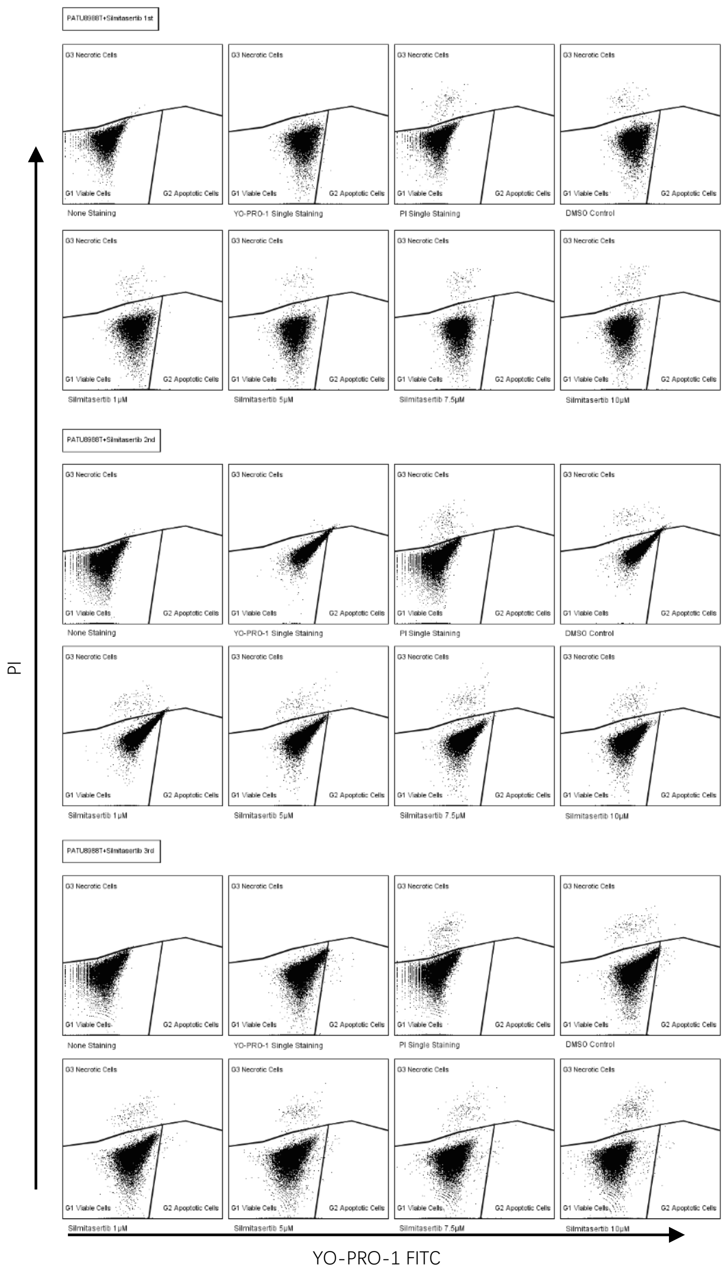

	1st		2nd		3rd		Mean (Cell Death)	SD	P-Value (vs. Control)
Concentration	G2(YP⁺/PI⁻)	G3(PI⁺)	G2(YP⁺/PI⁻)	G3(PI⁺)	G2(YP⁺/PI⁻)	G3(PI⁺)	G2+G3		G2+G3
Control	0.053	0.99	0.23	0.57	0.098	1.34	1.09	0.26	
1μM	0.032	1.00	0.17	0.74	0.062	1.21	1.07	0.15	>0.99
5μM	0.031	0.87	0.069	0.76	0.048	1.20	0.99	0.18	0.99
7.5μM	0.01	1.00	0.00687	0.94	0.062	1.25	1.09	0.16	>0.99
10μM	0.036	1.20	0.02	0.91	0.055	1.95	1.39	0.45	0.65

Supplementary Figure S7 – Apoptosis/Necrosis Dot Plot – Silmitasertib (6)

SU.86.86

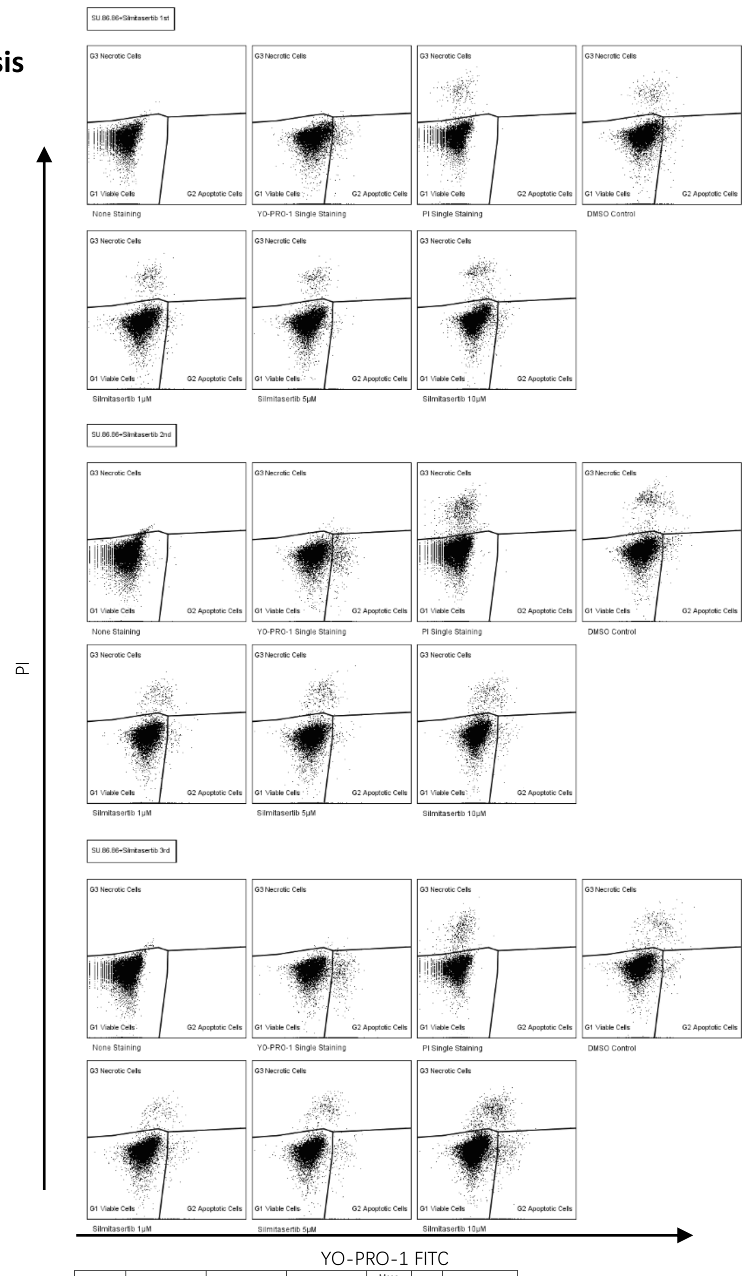

	1st		2nd		3rd		Mean (Cell Death)	SD	P-Value (vs. Control)
Concentration	G2(YP⁺/PI⁻)	G3(PI⁺)	G2(YP⁺/PI⁻)	G3(PI⁺)	G2(YP⁺/PI⁻)	G3(PI⁺)	G2+G3		G2+G3
Control	2.15	3.09	1.9	5.26	1.71	3.91	6.01	0.83	
1μM	0.89	3.06	1.54	4.24	2.69	4.62	5.68	1.37	0.96
5μM	0.86	2.97	1.29	2.77	2.03	2.31	4.08	0.21	0.10
10μM	1.38	4.20	1.18	4.15	1.92	3.48	5.44	0.11	0.82

T3M4

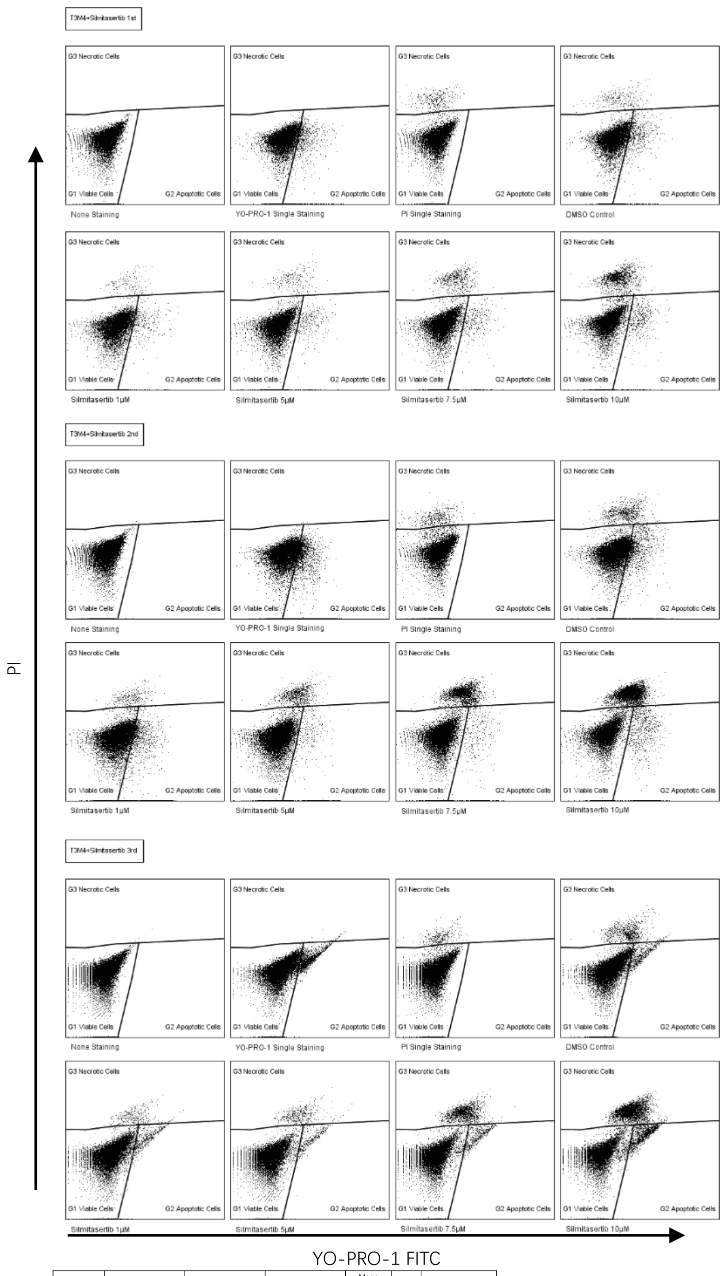

	1st		2nd		3rd		Mean (Cell Death)	SD	P-Value (vs. Control)
Concentration	G2(YP⁺/PI⁻)	G3(PI⁺)	G2(YP⁺/PI⁻)	G3(PI⁺)	G2(YP⁺/PI⁻)	G3(PI⁺)	G2+G3		G2+G3
Control	4.04	2.76	6.13	5.88	2.75	5.85	9.14	2.16	
1μM	2.44	1.54	7.27	2.14	2.12	2.03	5.85	2.52	0.89
5μM	2.44	2.34	2.49	5.74	1.51	2.85	5.79	1.73	0.88
7.5μM	2.64	5.65	2.59	16.3	2.41	10.3	13.30	4.35	0.79
10μM	3.5	14.3	5.26	33.9	6.12	24.9	29.33	8.80	0.005

Supplementary Figure S8 – Apoptosis/Necrosis Dot Plot – Dinaciclib (1)

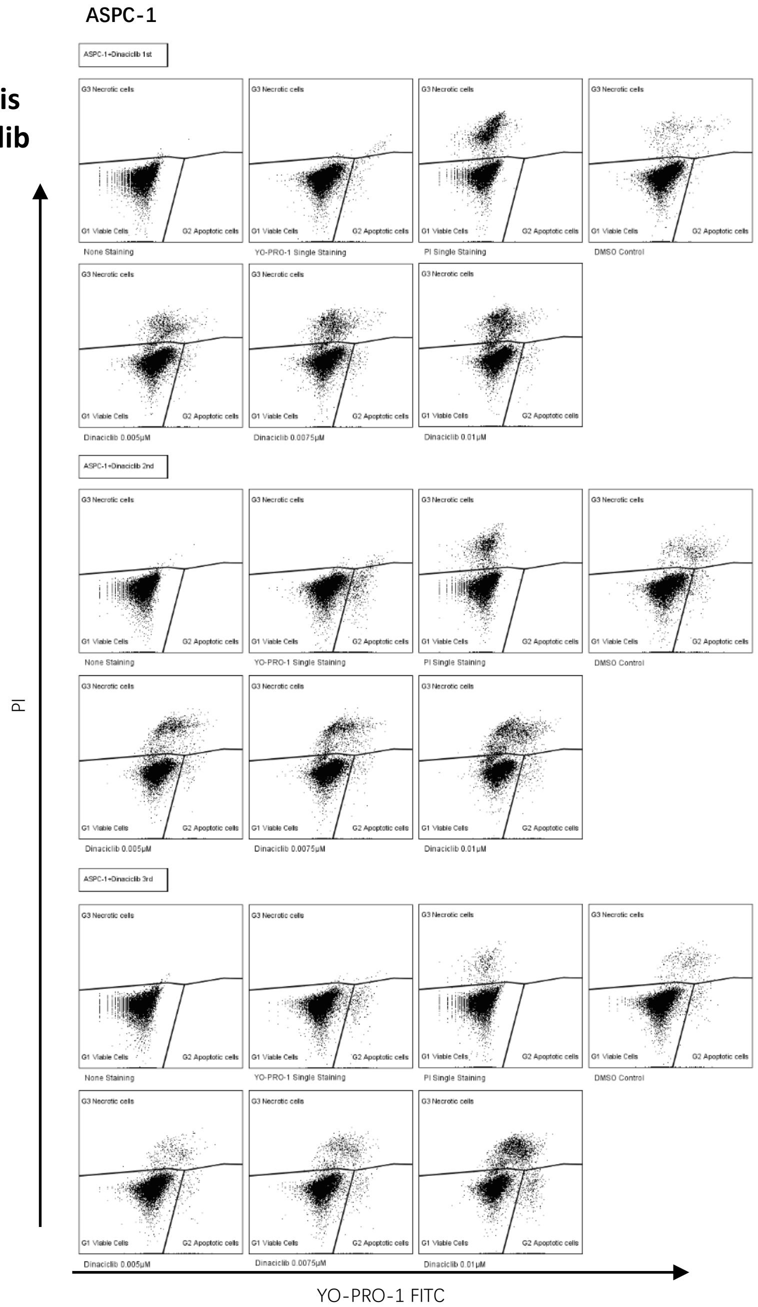

Concentration	1st G2(YP⁺/PI⁻)	1st G3(PI⁺)	2nd G2(YP⁺/PI⁻)	2nd G3(PI⁺)	3rd G2(YP⁺/PI⁻)	3rd G3(PI⁺)	Mean (Cell Death) G2+G3	SD	P-Value (vs. Control) G2+G3
Control	1.08	2.75	1.83	3.77	0.97	2.31	4.24	0.99	
0.005μM	2.34	6.83	1.35	10.7	0.90	3.24	8.45	3.27	0.29
0.0075μM	2.55	11.1	1.52	12.5	1.54	6.03	11.75	2.96	0.04
0.01μM	2.14	16.8	2.10	18.8	3.86	20.5	21.40	2.24	<0.001

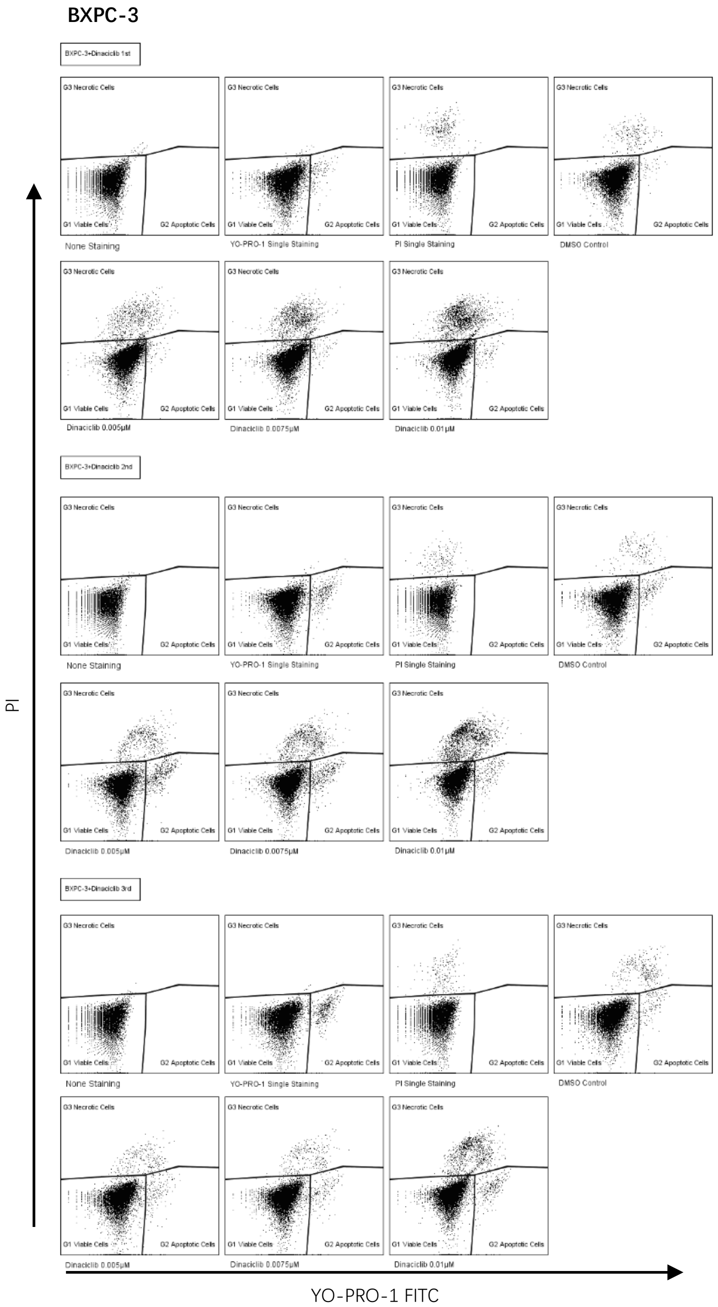

Concentration	1st G2(YP⁺/PI⁻)	1st G3(PI⁺)	2nd G2(YP⁺/PI⁻)	2nd G3(PI⁺)	3rd G2(YP⁺/PI⁻)	3rd G3(PI⁺)	Mean (Cell Death) G2+G3	SD	P-Value (vs. Control) G2+G3
Control	0.46	1.82	1.55	1.75	1.31	2.82	3.24	0.76	
0.005μM	1.35	6.64	4.04	4.33	1.68	2.19	6.74	2.04	0.39
0.0075μM	1.08	10.3	3.57	6.95	1.64	2.82	8.79	3.08	0.12
0.01μM	1.15	15.7	2.16	17.7	2.91	9.54	16.39	3.04	0.002

Supplementary Figure S8. Apoptosis/necrosis dot plot in 10 PDAC cell lines after 72h Dinaciclib exposure. The cell population strategy is based on the manufacturer's recommendation.
G1: Viable cells; G2: Apoptotic cells; G3: Necrotic cells.
Reference: https://www.thermofisher.com/ (accessed on 14. Oct. 2021)

Supplementary Figure S8 – Apoptosis/Necrosis Dot Plot – Dinaciclib (2)

CAPAN-1

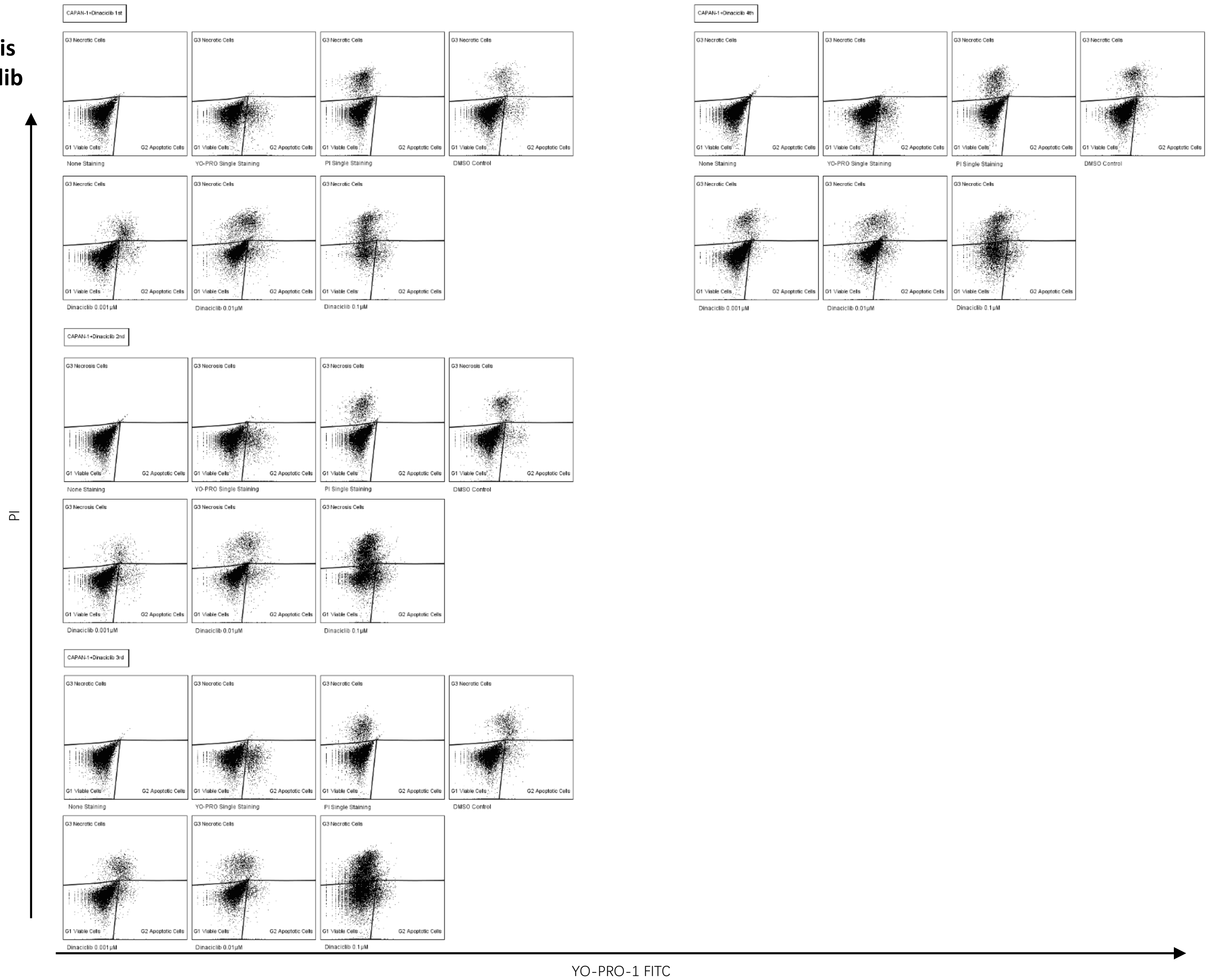

	1st		2nd		3rd		4th		Mean (Cell Death)	SD	P-Value (vs. Control)
Concentration	G2(YP⁺/PI⁻)	G3(PI⁺)	G2(YP⁺/PI⁻)	G3(PI⁺)	G2(YP⁺/PI⁻)	G3(PI⁺)	G2(YP⁺/PI⁻)	G3(PI⁺)	G2+G3		
Control	4.28	5.17	4.59	7.47	4.74	7.33	3.16	7.26	11.00	1.12	
0.001μM	5.20	6.33	5.23	3.75	5.23	8.22	3.14	7.87	11.24	1.59	>0.99
0.01μM	8.01	17.3	6.79	10.7	6.31	12.9	10.9	11.1	21.00	2.96	<0.001
0.1μM	7.71	37.2	7.09	41.8	8.09	37.6	12.3	34.9	46.67	1.52	<0.001

Supplementary Figure S8 – Apoptosis/Necrosis Dot Plot – Dinaciclib (3)

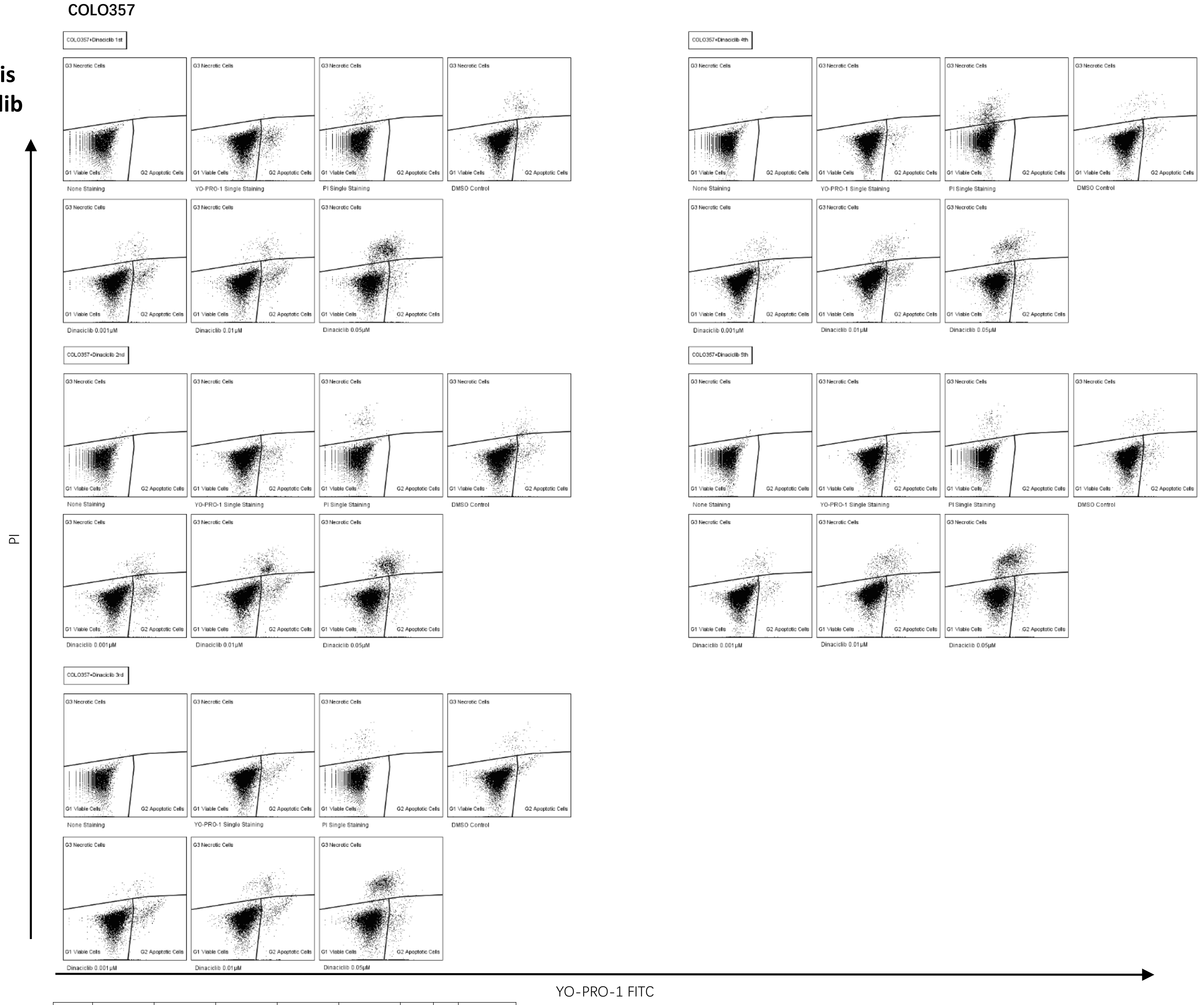

	1st		2nd		3rd		4th		5th		Mean (Cell Death)	SD	P-Value (vs. Control)
Concentration	G2(YP^+/PI^-)	G3(PI^+)	G2(YP^+/PI^-)	G3(PI^+)	G2(YP^+/PI^-)	G3(PI^+)	G2(YP^+/PI^-)	G3(PI^+)	G2(YP^+/PI^-)	G3(PI^+)	G2+G3		
Control	2.50	2.14	2.41	1.41	1.42	0.96	1.04	1.06	1.60	0.99	3.11	0.97	
0.001μM	3.85	1.60	4.65	3.13	4.47	1.01	1.86	1.71	1.20	1.45	4.99	1.77	0.37
0.01μM	5.06	2.00	4.82	5.51	4.00	2.84	3.21	2.87	2.91	4.71	7.59	1.46	0.01
0.05μM	1.96	12.7	1.70	10.4	2.89	12.3	2.02	7.01	2.22	14.8	13.60	2.77	<0.001

Supplementary Figure S8 – Apoptosis/Necrosis Dot Plot – Dinaciclib (4)

PANC-1

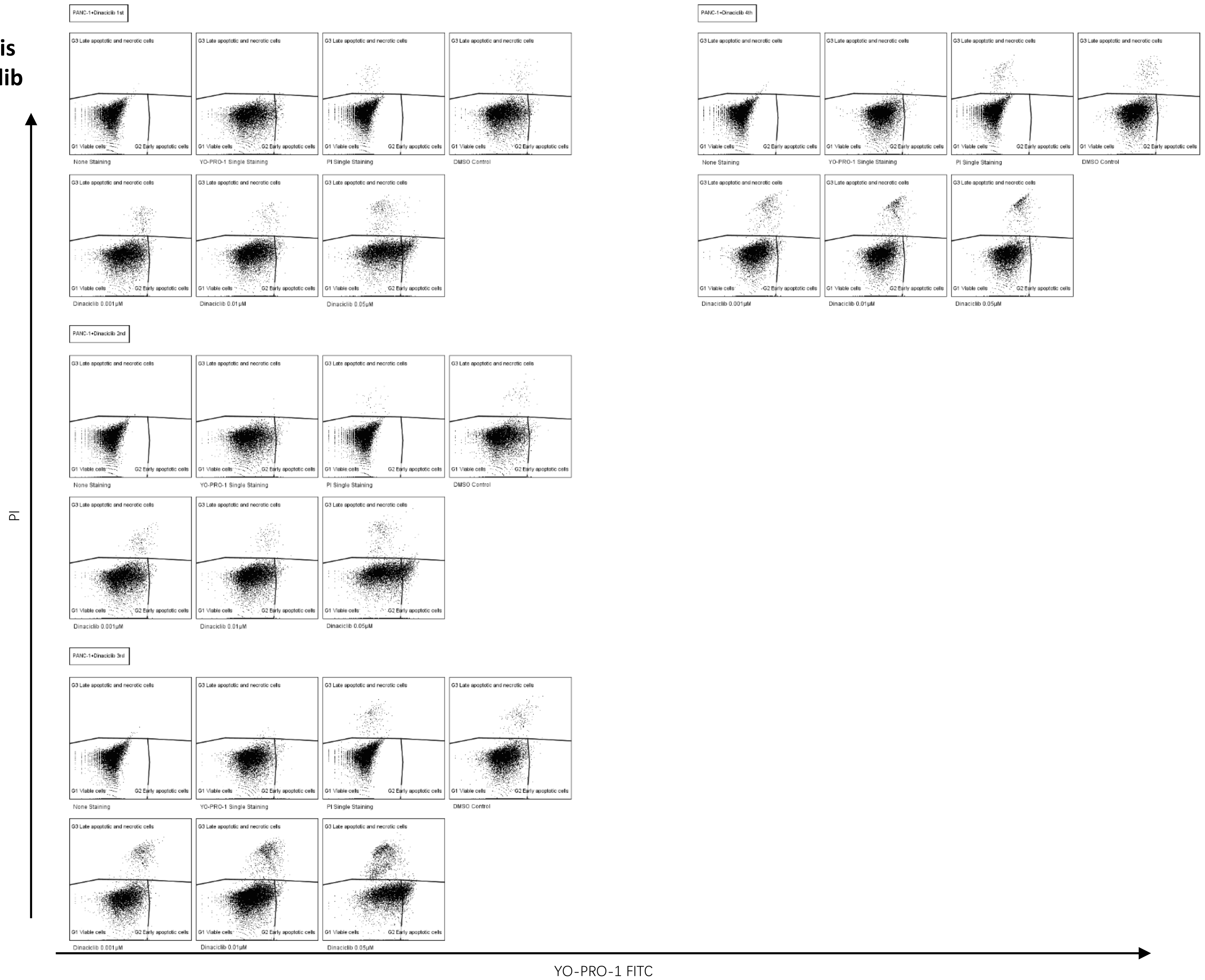

	1st		2nd		3rd		4th		Mean (Cell Death)	SD	P-Value (vs. Control)
Concentration	G2(YP⁺/PI⁻)	G3(PI⁺)	G2(YP⁺/PI⁻)	G3(PI⁺)	G2(YP⁺/PI⁻)	G3(PI⁺)	G2(YP⁺/PI⁻)	G3(PI⁺)	G2+G3		
Control	0.98	0.72	0.94	0.71	0.28	2.26	0.40	2.71	2.25	0.61	
0.001μM	0.48	1.92	0.50	1.84	0.39	6.29	0.46	9.79	5.42	3.30	0.63
0.01μM	1.53	1.43	1.61	1.43	0.96	6.16	1.00	8.44	5.64	2.76	0.58
0.1μM	10.6	3.60	10.5	3.09	12.4	14.9	0.48	10.7	16.57	6.30	0.002

Supplementary Figure S8 – Apoptosis/Necrosis Dot Plot – Dinaciclib (5)

PATU8902

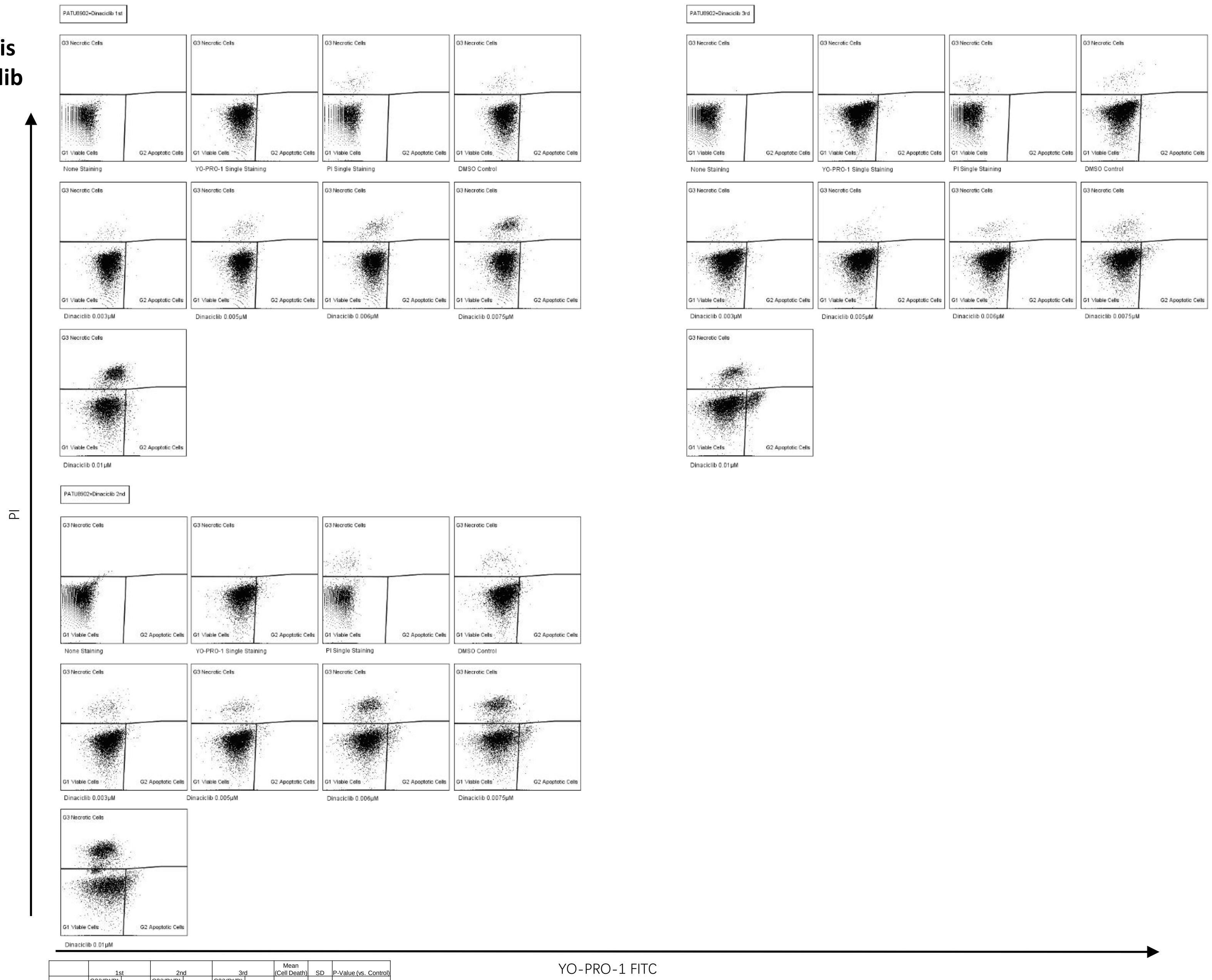

	1st		2nd		3rd		Mean (Cell Death)	SD	P-Value (vs. Control)
Concentration	G2(YP⁺/PI⁻)	G3(PI⁺)	G2(YP⁺/PI⁻)	G3(PI⁺)	G2(YP⁺/PI⁻)	G3(PI⁺)	G2+G3		G2+G3
Control	0.51	1.01	1.15	1.88	0.10	1.17	1.94	0.78	
0.003μM	0.26	1.81	0.55	3.17	0.19	0.41	2.13	1.27	>0.99
0.005μM	0.59	1.77	0.94	3.71	0.20	1.05	2.75	1.42	>0.99
0.006μM	0.65	3.29	2.75	12.1	0.31	1.34	6.81	5.76	0.75
0.0075μM	1.10	7.69	4.15	15.5	0.82	2.84	10.70	6.67	0.28
0.01μM	3.62	19.5	5.27	27.8	5.96	9.50	23.88	7.21	0.002

Supplementary Figure S8 – Apoptosis/Necrosis Dot Plot – Dinaciclib (6)

PATU8988S

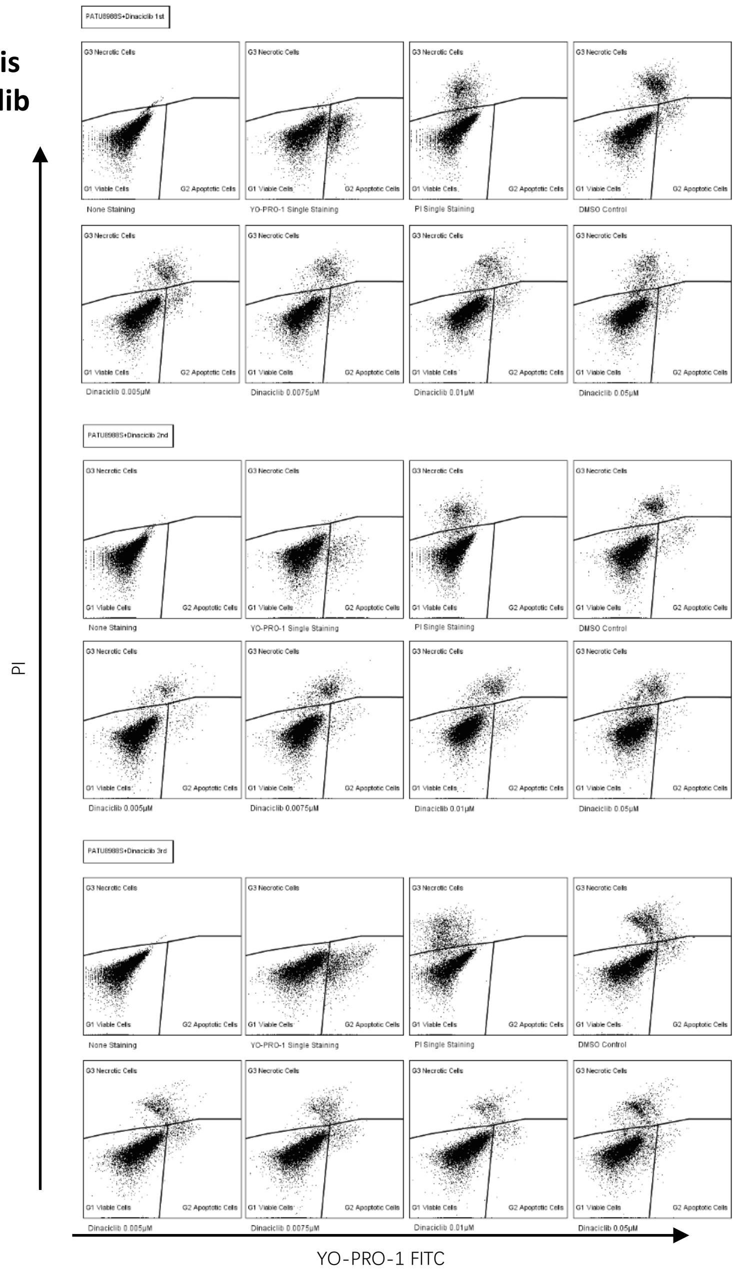

	1st		2nd		3rd		Mean (Cell Death)	SD	P-Value (vs. Control)
Concentration	G2(YP⁺/PI⁻)	G3(PI⁺)	G2(YP⁺/PI⁻)	G3(PI⁺)	G2(YP⁺/PI⁻)	G3(PI⁺)	G2+G3		G2+G3
Control	4.02	18.2	3.03	10.1	3.87	16.8	18.67	3.97	
0.005μM	4.23	9.84	2.00	5.21	3.60	9.40	11.43	3.01	0.04
0.0075μM	3.63	9.95	2.02	9.82	3.16	7.65	12.08	1.14	0.07
0.01μM	3.36	10.1	2.32	10.2	2.88	6.41	11.76	1.79	0.05
0.05μM	2.33	10.9	1.41	11.0	1.60	12.4	13.21	0.65	0.14

Supplementary Figure S8 – Apoptosis/Necrosis Dot Plot – Dinaciclib (7)

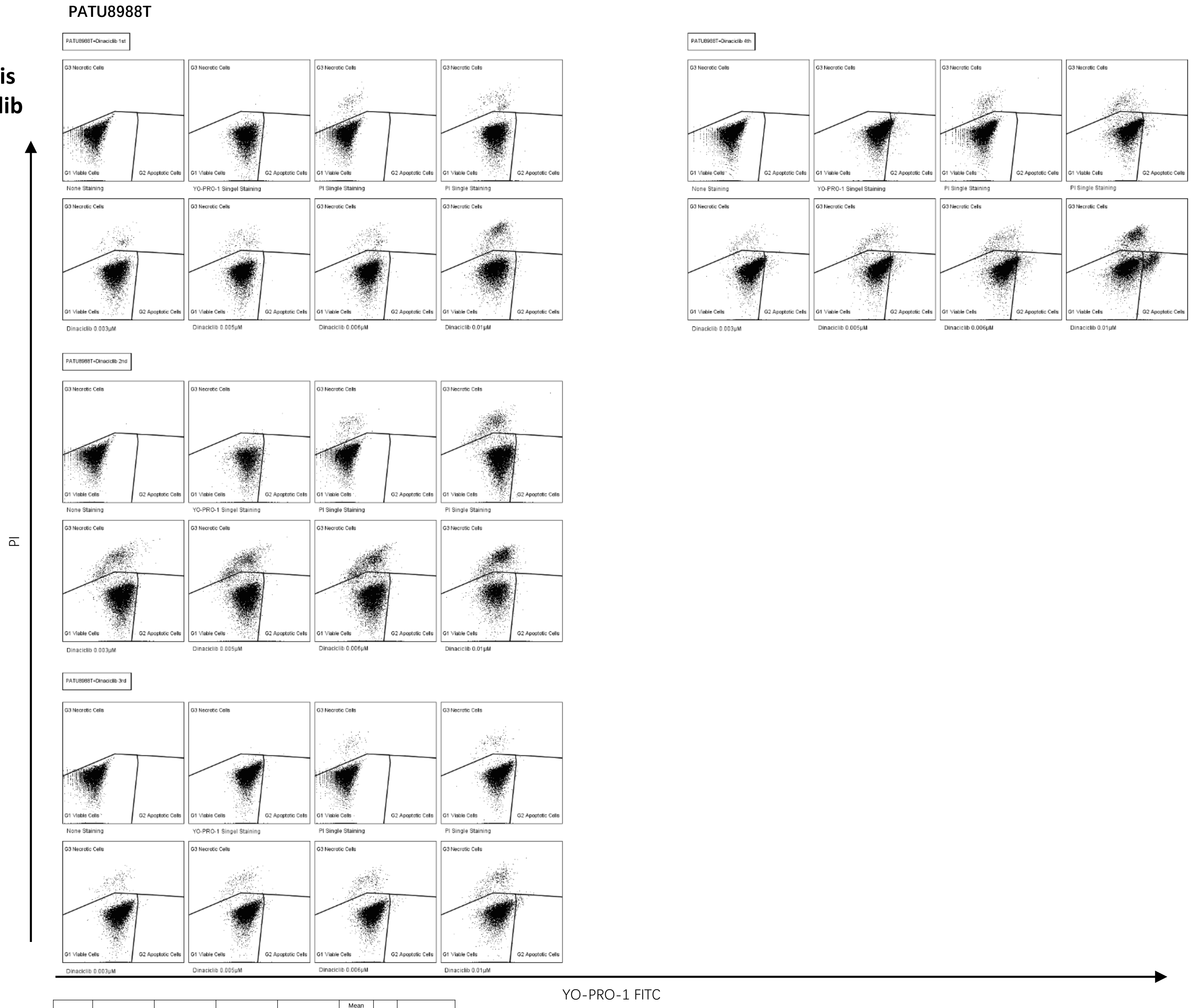

	1st		2nd		3rd		4th		Mean (Cell Death)	SD	P-Value (vs. Control)
Concentration	G2(YP+/PI-)	G3(PI+)	G2(YP+/PI-)	G3(PI+)	G2(YP+/PI-)	G3(PI+)	G2(YP+/PI-)	G3(PI+)	G2+G3		
Control	0.38	2.6	6.36	4.65	0.42	1.65	0.26	3.81	5.03	3.52	
0.003μM	0.14	1.61	4.03	6.08	0.28	1.65	0.32	2.18	4.07	3.50	>0.99
0.005μM	0.25	2.19	3.41	7.34	0.51	2.45	0.49	3.85	5.12	3.32	>0.99
0.006μM	0.35	2.3	3.72	9.30	0.56	2.80	0.91	4.76	6.18	4.11	>0.99
[illegible]	[illegible]	[illegible]	[illegible]	[illegible]	[illegible]	[illegible]	[illegible]	[illegible]	[illegible]	[illegible]	[illegible]

Supplementary Figure S8 – Apoptosis/Necrosis Dot Plot – Dinaciclib (8)

SU.86.86

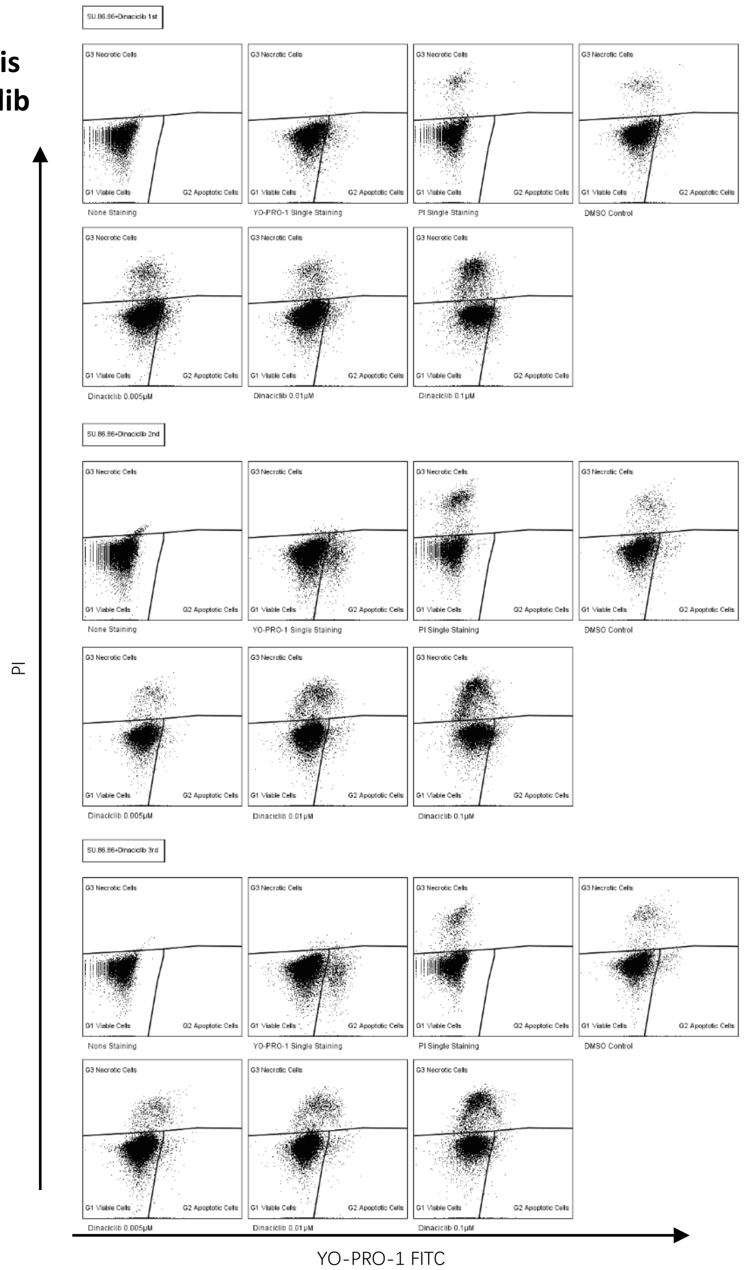

	1st		2nd		3rd		Mean (Cell Death)	SD	P-Value (vs. Control)
Concentration	G2(YP+/PI-)	G3(PI+)	G2(YP+/PI-)	G3(PI+)	G2(YP+/PI-)	G3(PI+)	G2+G3		G2+G3
Control	3.82	3.94	4.02	6.45	2.70	6.10	9.01	1.12	
0.005μM	6.06	6.96	3.67	5.97	2.86	4.89	10.14	2.18	0.85
0.01μM	3.98	6.77	3.74	11.3	2.30	8.71	12.27	1.96	0.21
0.1μM	4.74	20.7	4.46	24.0	2.51	23.4	26.60	1.33	<0.001

T3M4

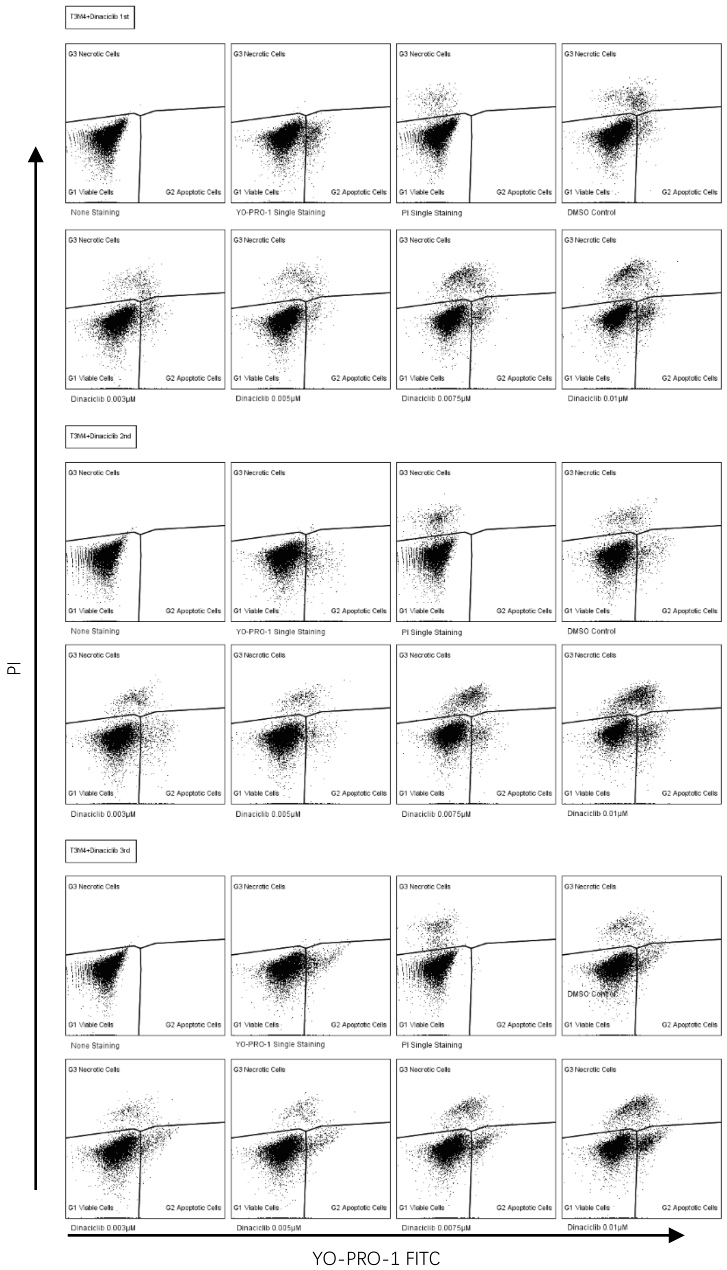

	1st		2nd		3rd		Mean (Cell Death)	SD	P-Value (vs. Control)
Concentration	G2(YP+/PI-)	G3(PI+)	G2(YP+/PI-)	G3(PI+)	G2(YP+/PI-)	G3(PI+)	G2+G3		G2+G3
Control	3.51	7.45	4.90	5.44	5.44	5.72	10.82	0.35	
0.003μM	4.53	4.93	5.80	4.77	5.55	3.65	9.74	0.59	0.90
0.005μM	3.16	5.63	3.93	4.64	4.53	3.55	8.48	0.30	0.43
0.0075μM	6.09	11.7	5.98	12.6	6.29	8.05	16.90	1.84	0.01
0.01μM	9.13	13.8	10.6	19.0	11.6	12.3	25.48	2.94	<0.001
