# Supplementary material for: Inhibitory Response to CK II Inhibitor Silmitasertib and CDKs Inhibitor Dinaciclib Is Related to Genetic Differences in Pancreatic Ductal Adenocarcinoma Cell Lines
Source: Int J Mol Sci. 2022 Apr 16;23(8):4409. doi: 10.3390/ijms23084409 (PMC9031017; doi:10.3390/ijms23084409)
Supplement: Supplementary file 1 [file ijms-23-04409-s001.zip › Supplementary Tables.pdf]

Supplementary Table S1 - Cell Viability Silmitasertib (to 100%Control)

AsPc-1

Proliferation

Concentration	1st	2nd	3rd	Mean	SD	P-value (vs. Control)
Control	100.00%	100.00%	100.00%	100.00%	0.00%	
1µM	123.02%	109.68%	130.43%	121.04%	10.52%	0.007
5µM	46.03%	41.94%	48.91%	45.63%	3.50%	<0.001
10µM	8.73%	18.28%	14.13%	13.71%	4.79%	<0.001

Metabolic activity

Concentration	1st	2nd	3rd	Mean	SD	P-value (vs. Control)
Control	100.00%	100.00%	100.00%	100.00%	0.00%	
1µM	107.30%	96.59%	87.61%	97.17%	9.86%	0.98
5µM	97.27%	70.69%	64.49%	77.48%	17.41%	0.08
10µM	33.40%	29.81%	46.14%	36.45%	8.58%	<0.001

Biomass

Concentration	1st	2nd	3rd	Mean	SD	P-value (vs. Control)
Control	100.00%	100.00%	100.00%	100.00%	0.00%	
1µM	60.52%	81.24%	66.02%	69.26%	10.73%	0.002
5µM	33.51%	34.12%	17.02%	28.22%	9.70%	<0.001
10µM	9.05%	5.08%	11.51%	8.55%	3.24%	<0.001

BxPc-3

Proliferation

Concentration	1st	2nd	3rd	4th	5th	Mean	SD	P-value (vs. Control)
Control	100.00%	100.00%	100.00%	100.00%	100.00%	100.00%	0.00%	
1µM	92.51%	77.69%	81.30%	67.61%	76.80%	79.18%	9.00%	<0.001
2.5µM			42.37%	46.95%	47.20%	45.51%	2.72%	<0.001
5µM	18.89%	14.74%	16.41%	14.55%	12.00%	15.32%	2.54%	<0.001
10µM	2.28%	6.77%	3.82%	5.63%	8.00%	5.30%	2.28%	<0.001

Metabolic activity

Concentration	1st	2nd	3rd	4th	5th	Mean	SD	P-value (vs. Control)
Control	100.00%	100.00%	100.00%	100.00%	100.00%	100.00%	0.00%	
1µM	105.64%	97.94%	107.29%	108.76%	113.86%	106.70%	5.78%	0.58
2.5µM			97.84%	109.17%	92.99%	100.00%	8.30%	>0.99
5µM	74.78%	52.19%	52.91%	82.90%	77.32%	68.02%	14.43%	<0.001
10µM	34.82%	21.27%	20.03%	29.97%	38.62%	28.94%	8.18%	<0.001

Biomass

Concentration	1st	2nd	3rd	4th	5th	Mean	SD	P-value (vs. Control)
Control	100.00%	100.00%	100.00%	100.00%	100.00%	100.00%	0.00%	
1µM	57.69%	56.46%	61.48%	69.95%	77.37%	64.59%	8.88%	<0.001
2.5µM			36.14%	44.12%	49.23%	43.16%	6.60%	<0.001
5µM	21.16%	9.15%	13.54%	22.89%	24.84%	18.32%	6.68%	<0.001
10µM	5.89%	0.88%	6.04%	9.35%	12.23%	6.88%	4.25%	<0.001

Capan-1

Proliferation

Concentration	1st	2nd	3rd	Mean	SD	P-value (vs. Control)
Control	100.00%	100.00%	100.00%	100.00%	0.00%	
1µM	84.72%	83.33%	82.35%	83.47%	1.19%	0.09
5µM	75.00%	83.67%	72.94%	77.20%	5.69%	0.02
10µM	36.11%	11.00%	39.12%	28.74%	15.44%	<0.001

Metabolic activity

Concentration	1st	2nd	3rd	Mean	SD	P-value (vs. Control)
Control	100.00%	100.00%	100.00%	100.00%	0.00%	
1µM	91.96%	107.98%	90.58%	96.84%	9.67%	0.95
5µM	88.29%	84.11%	108.45%	93.62%	13.01%	>0.99
10µM	37.10%	52.92%	58.55%	49.52%	11.12%	<0.001

Biomass

Concentration	1st	2nd	3rd	Mean	SD	P-value (vs. Control)
Control	100.00%	100.00%	100.00%	100.00%	0.00%	
1µM	86.16%	69.46%	103.43%	86.35%	16.99%	0.38
5µM	50.96%	47.88%	70.15%	56.33%	12.07%	0.004
10µM	22.84%	19.29%	36.38%	26.17%	9.02%	<0.001

Colo357

Proliferation

Concentration	1st	2nd	3rd	4th	5th	Mean	SD	P-value (vs. Control)
Control	100.00%	100.00%	100.00%	100.00%	100.00%	100.00%	0.00%	
1µM	117.56%	119.66%	107.72%	94.35%	94.04%	106.67%	12.24%	0.43
5µM	83.59%	98.31%	92.28%	91.13%	82.46%	89.55%	6.57%	0.13
10µM	77.10%	62.36%	76.06%	73.39%	61.40%	70.06%	7.60%	<0.001

Metabolic activity

Concentration	1st	2nd	3rd	4th	5th	Mean	SD	P-value (vs. Control)
Control	100.00%	100.00%	100.00%	100.00%	100.00%	100.00%	0.00%	
1µM	115.06%	100.47%	98.38%	108.54%	96.82%	103.85%	7.72%	0.93
5µM		81.90%	101.10%	112.36%		98.45%	15.40%	>0.99
10µM	96.96%	64.77%	98.50%	89.06%	60.09%	81.88%	18.18%	0.08

Biomass

Concentration	1st	2nd	3rd	Mean	SD	P-value (vs. Control)
Control	100.00%	100.00%	100.00%	100.00%	0.00%	
1µM	99.45%	75.34%	89.23%	88.01%	12.10%	0.39
5µM	98.20%	74.78%	69.11%	80.70%	15.42%	0.11
10µM	59.55%	49.92%	58.24%	55.90%	5.22%	0.002

Supplementary Table S1 - Cell Viability Silmitasertib (to 100%Control)

Panc-1

	Proliferation					
Concentration	1st	2nd	3rd	Mean	SD	P-value (vs. Control)
Control	100.00%	100.00%	100.00%	100.00%	0.00%	
1µM	101.25%	126.09%	102.10%	109.81%	14.10%	0.83
5µM	98.75%	91.30%	79.00%	89.68%	9.97%	0.81
10µM	97.50%	38.04%	63.50%	66.35%	29.83%	0.1
	Metabolic activity					
Concentration	1st	2nd	3rd	Mean	SD	P-value (vs. Control)
Control	100.00%	100.00%	100.00%	100.00%	0.00%	
1µM	101.00%	96.52%	93.03%	96.85%	4.00%	0.93
5µM	82.96%	88.07%	80.29%	83.77%	3.95%	<0.001
10µM	69.87%	66.91%	68.56%	68.45%	1.48%	<0.001
	Biomass					
Concentration	1st	2nd	3rd	Mean	SD	P-value (vs. Control)
Control	100.00%	100.00%	100.00%	100.00%	0.00%	
1µM	101.80%	84.02%	104.34%	96.72%	11.07%	0.93
5µM	64.30%	72.88%	86.97%	74.72%	11.45%	0.02
10µM	39.21%	38.47%	49.95%	42.54%	6.43%	<0.001

PaTu8902

	Proliferation							
Concentration	1st	2nd	3rd	4th	5th	Mean	SD	P-value (vs. Control)
Control	100.00%	100.00%	100.00%	100.00%	100.00%	100.00%	0.00%	
1µM	89.89%	98.00%	90.38%	89.47%	96.96%	92.94%	4.17%	0.06
5µM	78.65%	87.00%	75.00%	72.37%	73.39%	77.28%	5.93%	<0.001
10µM	65.17%	54.00%	63.46%	59.87%	54.82%	59.46%	5.00%	<0.001
	Metabolic activity							
Concentration	1st	2nd	3rd	4th	5th	Mean	SD	P-value (vs. Control)
Control	100.00%	100.00%	100.00%	100.00%	100.00%	100.00%	0.00%	
1µM	94.73%	98.86%	88.65%	94.21%	100.28%	95.35%	4.56%	0.88
5µM	75.65%	87.50%	70.59%	92.68%	78.90%	81.06%	8.95%	0.07
10µM	85.61%	42.71%	26.71%	62.89%	47.03%	52.99%	22.33%	<0.001
	Biomass							
Concentration	1st	2nd	3rd	4th		Mean	SD	P-value (vs. Control)
Control	100.00%	100.00%	100.00%	100.00%		100.00%	0.00%	
1µM	94.42%	98.41%	98.48%	87.80%		94.78%	5.02%	0.93
5µM	80.61%	94.43%	71.55%	59.09%		76.42%	14.90%	0.11
10µM	71.85%	84.57%	26.84%	47.32%		57.65%	25.71%	0.005

PaTu8988S

	Proliferation						
Concentration	1st	2nd	3rd	4th	Mean	SD	P-value (vs. Control)
Control	100.00%	100.00%	100.00%	100.00%	100.00%	0.00%	
1µM	130.51%	121.36%	108.70%	104.65%	116.31%	11.85%	0.01
5µM	83.05%	76.70%	75.00%	84.88%	79.91%	4.79%	0.004
10µM	71.19%	66.02%	67.39%	76.74%	70.34%	4.80%	<0.001
	Metabolic activity						
Concentration	1st	2nd	3rd	4th	Mean	SD	P-value (vs. Control)
Control	100.00%	100.00%	100.00%	100.00%	100.00%	0.00%	
1µM	95.43%	119.78%	119.56%	98.74%	108.38%	13.11%	0.74
5µM	77.93%	91.42%	128.89%	106.14%	101.10%	21.82%	>0.99
10µM	74.04%	88.90%	101.14%	97.78%	90.47%	12.11%	0.67
	Biomass						
Concentration	1st	2nd	3rd	4th	Mean	SD	P-value (vs. Control)
Control	100.00%	100.00%	100.00%	100.00%	100.00%	0.00%	
1µM	89.29%	89.36%	94.85%	97.36%	92.72%	4.05%	0.21
5µM	78.42%	68.05%	85.24%	83.27%	78.75%	7.68%	<0.001
10µM	56.41%	61.34%	44.47%	52.74%	53.74%	7.11%	<0.001

PaTu8988T

	Proliferation							
Concentration	1st	2nd	3rd	4th		Mean	SD	P-value (vs. Control)
Control	100.00%	100.00%	100.00%	100.00%		100.00%	0.00%	
1µM	104.90%	92.66%	95.04%	94.01%		96.65%	5.58%	0.82
5µM	78.88%	82.04%	76.86%	79.21%		79.25%	2.13%	0.003
10µM	56.50%	61.75%	34.73%	44.63%		49.40%	12.12%	<0.001
	Metabolic activity							
Concentration	1st	2nd	3rd	4th		Mean	SD	P-value (vs. Control)
Control	100.00%	100.00%	100.00%	100.00%		100.00%	0.00%	
1µM	70.49%	102.13%	80.82%	117.84%		92.82%	21.25%	0.76
5µM	103.14%	87.52%	87.46%	113.44%		97.89%	12.72%	>0.99
10µM	60.66%	69.11%	64.81%	67.84%		65.61%	3.76%	0.006
	Biomass							
Concentration	1st	2nd	3rd	4th	5th	Mean	SD	P-value (vs. Control)
Control	100.00%	100.00%	100.00%	100.00%	100.00%	100.00%	0.00%	
1µM	71.41%	77.94%	96.54%	81.91%	90.69%	83.70%	10.01%	0.07
5µM	55.38%	64.06%	76.74%	79.34%	78.88%	70.88%	10.68%	0.001
10µM	32.96%	58.63%	68.36%	36.54%	54.50%	50.20%	15.03%	<0.001

Supplementary Table S1 - Cell Viability Silmitasertib (to 100%Control)

SU.86.86

	Proliferation					
Concentration	1st	2nd	3rd	Mean	SD	P-value (vs. Control)
Control	100.00%	100.00%	100.00%	100.00%	0.00%	
1µM	97.93%	104.05%	80.38%	94.12%	12.29%	0.73
5µM	72.41%	75.00%	61.39%	69.60%	7.23%	0.005
10µM	46.90%	44.59%	31.01%	40.83%	8.59%	<0.001
	Metabolic activity					
Concentration	1st	2nd	3rd	Mean	SD	P-value (vs. Control)
Control	100.00%	100.00%	100.00%	100.00%	0.00%	
1µM	90.06%	101.06%	98.64%	96.59%	5.78%	>0.99
5µM	81.88%	72.17%	58.51%	70.85%	11.74%	0.04
10µM	71.87%	47.80%	32.30%	50.66%	19.94%	0.002
	Biomass					
Concentration	1st	2nd	3rd	Mean	SD	P-value (vs. Control)
Control	100.00%	100.00%	100.00%	100.00%	0.00%	
1µM	101.36%	94.56%	93.21%	96.38%	4.37%	0.96
5µM	78.27%	72.59%	53.28%	68.05%	13.10%	0.03
10µM	53.20%	19.43%	21.03%	31.22%	19.05%	<0.001

T3M4

	Proliferation						
Concentration	1st	2nd	3rd	4th	Mean	SD	P-value (vs. Control)
Control	100.00%	100.00%	100.00%	100.00%	100.00%	0.00%	
1µM	149.04%	125.00%	132.14%	119.05%	131.31%	12.98%	0.001
5µM	77.88%	62.50%	91.43%	81.48%	78.32%	12.00%	0.02
10µM	44.71%	37.50%	51.07%	48.41%	45.42%	5.89%	<0.001
	Metabolic activity						
Concentration	1st	2nd	3rd	4th	Mean	SD	P-value (vs. Control)
Control	100.00%	100.00%	100.00%	100.00%	100.00%	0.00%	
1µM	135.94%	126.00%	119.44%	101.79%	120.79%	14.37%	0.02
5µM	115.05%	116.35%	108.39%	92.86%	108.16%	10.78%	<0.001
10µM	86.76%	37.15%	75.80%	81.38%	70.27%	22.53%	<0.001
	Biomass						
Concentration	1st	2nd	3rd	4th	Mean	SD	P-value (vs. Control)
Control	100.00%	100.00%	100.00%	100.00%	100.00%	0.00%	
1µM	94.04%	98.39%	96.61%	95.34%	96.10%	1.86%	0.6
5µM	53.04%	58.82%	55.02%	59.01%	56.47%	2.93%	<0.001
10µM	27.04%	10.68%	30.22%	32.46%	25.10%	9.87%	<0.001

Supplementary Table S2 - IC50 Silmitasertib (µM)

	Proliferation	Metabolic Activity	Cell Biomass
**AsPc-1**	4.855	8.769	2.019
**BxPc-3**	2.131	6.949	1.691
**Capan-1**	7.426	9.951	5.281
**Colo357**	15.01	14.78	14.32
**Panc-1**	13.34	19.01	8.615
**PaTu8902**	14.84	10.78	13.09
**PaTu8988S**	16.20	38.73	11.50
**PaTu8988T**	9.925	11.51	11.89
**T3M4**	9.087	10.53	5.697
**SU.86.86**	8.091	10.07	7.006

Supplementary Table S3 - Cell Viability Dinaciclib (to 100%Control)

AsPc-1

	Proliferation						
Concentration	1st	2nd	3rd	4th	Mean	SD	P-value (vs. Control)
Control	100.00%	100.00%	100.00%	100.00%	100.00%	0.00%	
0.005 µM		111.49%	102.91%	105.53%	106.64%	4.40%	0.45
0.0075 µM		56.17%	47.57%	56.60%	53.45%	5.09%	<0.001
0.01µM	6.70%	27.23%	19.42%	32.77%	21.53%	11.30%	<0.001
0.1µM	1.79%	0.00%	2.91%	0.00%	1.18%	1.43%	<0.001
	Metabolic activity						
Concentration	1st	2nd	3rd	4th	Mean	SD	P-value (vs. Control)
Control	100.00%	100.00%	100.00%	100.00%	100.00%	0.00%	
0.005 µM		105.46%	106.48%	108.34%	106.76%	1.46%	0.93
0.0075 µM		96.94%	81.73%	105.05%	94.57%	11.84%	0.97
0.01µM	8.43%	58.12%	72.37%	30.27%	42.30%	28.56%	<0.001
0.1µM	1.92%	3.93%	1.52%	5.82%	3.30%	1.98%	<0.001
	Biomass						
Concentration	1st	2nd	3rd	4th	Mean	SD	P-value (vs. Control)
Control	100.00%	100.00%	100.00%	100.00%	100.00%	0.00%	
0.005 µM		92.92%	89.65%	86.52%	89.70%	3.20%	0.31
0.0075 µM		68.31%	42.00%	40.35%	50.22%	15.69%	0.001
0.01µM	2.80%	16.14%	24.72%	6.42%	12.52%	9.89%	0.001
0.1µM	0.00%	0.27%	0.83%	0.66%	0.44%	0.38%	<0.001

BxPc-3

	Proliferation					
Concentration	1st	2nd	3rd	Mean	SD	P-value (vs. Control)
Control	100.00%	100.00%	100.00%	100.00%	0.00%	
0.005 µM	104.76%	102.91%	109.00%	105.56%	3.12%	0.4
0.0075 µM	48.48%	47.57%	58.77%	51.61%	6.22%	<0.001
0.01µM	32.47%	19.42%	30.33%	27.41%	7.00%	<0.001
0.1µM	1.30%	2.91%	0.47%	1.56%	1.24%	<0.001
	Metabolic activity					
Concentration	1st	2nd	3rd	Mean	SD	P-value (vs. Control)
Control	100.00%	100.00%	100.00%	100.00%	0.00%	
0.005 µM	92.33%	104.15%	93.37%	96.62%	6.54%	>0.99
0.0075 µM	86.78%	97.44%	74.93%	86.38%	11.26%	0.59
0.01µM	61.05%	77.35%	22.59%	53.66%	28.12%	0.007
0.1µM	2.73%	2.39%	2.10%	2.41%	0.32%	<0.001
	Biomass					
Concentration	1st	2nd	3rd	Mean	SD	P-value (vs. Control)
Control	100.00%	100.00%	100.00%	100.00%	0.00%	
0.005 µM	99.53%	91.55%	96.67%	95.92%	4.04%	0.97
0.0075 µM	87.18%	65.94%	47.04%	66.72%	20.08%	0.01
0.01µM	20.03%	34.31%	7.31%	20.55%	13.51%	<0.001
0.1µM	0.15%	0.26%	0.10%	0.17%	0.08%	<0.001

Capan-1

	Proliferation					
Concentration	1st	2nd	3rd	Mean	SD	P-value (vs. Control)
Control	100.00%	100.00%	100.00%	100.00%	0.00%	
0.001µM	102.67%	92.94%	97.96%	97.86%	4.87%	0.97
0.01µM	48.00%	32.94%	51.02%	43.99%	9.69%	<0.001
0.1µM	4.00%	8.24%	5.10%	5.78%	2.20%	<0.001
1µM	1.30%	6.33%	7.29%	4.97%	3.22%	<0.001
	Metabolic activity					
Concentration	1st	2nd	3rd	Mean	SD	P-value (vs. Control)
Control	100.00%	100.00%	100.00%	100.00%	0.00%	
0.001µM	109.49%	93.77%	98.31%	100.52%	8.09%	>0.99
0.01µM	54.20%	79.77%	74.24%	69.40%	13.45%	0.004
0.1µM	10.73%	17.10%	10.41%	12.75%	3.77%	<0.001
1µM	13.43%	21.22%	1.36%	12.00%	10.01%	<0.001
	Biomass					
Concentration	1st	2nd	3rd	Mean	SD	P-value (vs. Control)
Control	100.00%	100.00%	100.00%	100.00%	0.00%	
0.001µM	83.70%	78.14%	87.86%	83.23%	4.88%	0.01
0.01µM	23.94%	32.86%	45.04%	33.95%	10.59%	<0.001
0.1µM	0.97%	1.63%	2.32%	1.64%	0.68%	<0.001
1µM	2.08%	5.89%	0.40%	2.79%	2.81%	<0.001

Supplementary Table S3 - Cell Viability Dinaciclib (to 100%Control)

Colo357

Proliferation

Concentration	1st	2nd	3rd	Mean	SD	P-value (vs. Control)
Control	100.00%	100.00%	100.00%	100.00%	0.00%	
0.001μM	59.46%	45.56%	61.75%	55.59%	8.76%	<0.001
0.01μM	10.42%	10.81%	10.88%	10.70%	0.25%	<0.001
0.05μM	6.56%	5.08%	9.82%	7.15%	2.43%	<0.001
0.1μM	6.95%	2.02%	2.46%	3.81%	2.73%	<0.001

Metabolic activity

Concentration	1st	2nd	3rd	4th	Mean	SD	P-value (vs. Control)
Control	100.00%	100.00%	100.00%	100.00%	100.00%	0.00%	
0.001μM	94.12%	76.57%	77.64%	60.56%	77.22%	13.71%	<0.001
0.01μM	39.40%	51.11%	40.57%	33.56%	41.16%	7.31%	<0.001
0.05μM	8.79%	11.72%	7.42%	10.59%	9.63%	1.90%	<0.001
0.1μM	15.01%	11.58%	7.92%	9.08%	10.90%	3.14%	<0.001

Biomass

Concentration	1st	2nd	3rd	4th	Mean	SD	P-value (vs. Control)
Control	100.00%	100.00%	100.00%	100.00%	100.00%	0.00%	
0.001μM	73.52%	64.38%	70.06%	54.69%	65.66%	8.23%	<0.001
0.01μM	16.70%	34.38%	14.10%	17.54%	20.68%	9.25%	<0.001
0.05μM	7.01%	9.10%	4.75%	9.75%	7.65%	2.26%	<0.001
0.1μM	13.17%	6.71%	2.33%	11.12%	8.33%	4.82%	<0.001

Panc-1

Proliferation

Concentration	1st	2nd	3rd	4th	5th	6th	7th	8th	9th	Mean	SD	P-value (vs. Control)
Control	100.00%	100.00%	100.00%	100.00%	100.00%	100.00%	100.00%	100.00%	100.00%	100.00%	0.00%	
0.001μM	124.09%	118.52%	111.56%							118.06%	6.28%	<0.001
0.01μM							53.19%	49.56%	41.55%	48.10%	5.96%	<0.001
0.025μM							20.21%	27.21%	19.01%	22.14%	4.43%	<0.001
0.05μM				13.24%	15.27%	12.33%	14.36%	19.91%	14.08%	14.87%	2.67%	<0.001
0.1μM	12.41%	5.93%	13.61%	2.94%	12.21%	5.48%				8.76%	4.50%	<0.001
1μM	1.46%	2.96%	8.16%							4.19%	3.52%	<0.001

Metabolic activity

Concentration	1st	2nd	3rd	4th	5th	6th	7th	8th	9th	Mean	SD	P-value (vs. Control)
Control	100.00%	100.00%	100.00%	100.00%	100.00%	100.00%	100.00%	100.00%	100.00%	100.00%	0.00%	
0.001μM	95.16%	96.48%	99.26%							96.97%	2.09%	0.92
0.01μM	88.42%	94.71%	100.45%	103.71%	92.06%	108.72%	84.54%	86.16%	93.61%	94.71%	8.17%	0.19
0.025μM							31.23%	29.89%	40.43%	33.85%	5.74%	<0.001
0.05μM				27.01%	29.64%	26.40%	18.75%	22.35%	23.01%	24.53%	3.90%	<0.001
0.1μM	23.14%	21.72%	32.10%	30.80%	22.25%	15.99%				24.33%	6.07%	<0.001
1μM	15.31%	19.76%	13.18%							16.08%	3.36%	<0.001

Biomass

Concentration	1st	2nd	3rd	4th	5th	6th	7th	8th	9th	Mean	SD	P-value (vs. Control)
Control	100.00%	100.00%	100.00%	100.00%	100.00%	100.00%	100.00%	100.00%	100.00%	100.00%	0.00%	
0.001μM	83.15%	108.16%	107.15%							99.49%	14.16%	>0.99
0.01μM	53.16%	83.66%	85.13%	78.38%	68.46%	97.42%	64.76%	48.58%	34.13%	68.19%	20.21%	<0.001
0.025μM							20.14%	15.36%	14.62%	16.71%	3.00%	<0.001
0.05μM				14.02%	8.96%	8.57%	7.18%	8.90%	9.29%	9.49%	2.34%	<0.001
0.1μM	4.92%	1.66%	3.12%	11.04%	6.41%	8.63%				5.96%	3.49%	<0.001
1μM	7.24%	2.00%	2.31%							3.85%	2.94%	<0.001

PaTu8902

Proliferation

Concentration	1st	2nd	3rd	4th	5th	Mean	SD	P-value (vs. Control)
Control	100.00%	100.00%	100.00%	100.00%	100.00%	100.00%	0.00%	
0.001 μM	78.65%	105.00%	109.62%	93.42%	93.75%	96.09%	12.03%	0.79
0.0025 μM		86.00%	81.73%	77.63%	79.46%	81.21%	3.61%	0.003
0.005 μM	24.99%	43.00%	45.58%	38.16%	43.75%	39.10%	8.35%	<0.001
0.0075 μM	5.62%	11.00%	9.62%	3.95%	9.82%	8.00%	3.04%	<0.001

Metabolic activity

Concentration	1st	2nd	3rd	Mean	SD	P-value (vs. Control)
Control	100.00%	100.00%	100.00%	100.00%	0.00%	
0.001 μM	91.13%	82.95%	75.05%	83.04%	8.04%	0.14
0.0025 μM	97.16%	72.17%	67.64%	78.99%	15.90%	0.06
0.005 μM	63.19%	54.84%	60.56%	59.53%	4.27%	0.001
0.0075 μM	30.19%	11.68%	17.96%	19.94%	9.41%	<0.001

Biomass

Concentration	1st	2nd	3rd	4th	Mean	SD	P-value (vs. Control)
Control	100.00%	100.00%	100.00%	100.00%	100.00%	0.00%	
0.001 μM	70.23%	85.63%	89.48%	77.68%	80.76%	8.57%	0.12
0.0025 μM		92.82%	75.99%	63.14%	77.32%	14.88%	0.08
0.005 μM	23.59%	70.78%	46.78%	55.58%	49.18%	19.73%	<0.001
0.0075 μM	8.04%	12.80%	7.24%	23.02%	12.78%	7.26%	<0.001

Supplementary Table S3 - Cell Viability Dinaciclib (to 100%Control)

PaTu8988S

Proliferation

Concentration	1st	2nd	3rd	4th	5th	6th	Mean	SD	P-value (vs. Control)
Control	100.00%	100.00%	100.00%	100.00%	100.00%	100.00%	100.00%	0.00%	
0.001 µM	72.88%	93.55%	95.65%				87.36%	12.58%	0.16
0.005 µM	67.80%	88.17%	84.78%	95.35%	80.77%	79.73%	82.77%	9.26%	0.007
0.0075 µM	50.85%	69.89%	67.39%	82.09%	69.23%	67.57%	67.84%	9.98%	<0.001
0.01 µM	42.37%	30.11%	34.78%	55.35%	44.87%	33.78%	40.21%	9.26%	<0.001
0.025 µM				12.33%	7.69%	6.76%	8.93%	2.98%	<0.001

Metabolic activity

Concentration	1st	2nd	3rd	4th	5th	6th	Mean	SD	P-value (vs. Control)
Control	100.00%	100.00%	100.00%	100.00%	100.00%	100.00%	100.00%	0.00%	
0.001 µM	73.54%	89.51%	77.02%				80.02%	8.40%	0.007
0.005 µM	71.72%	73.91%	68.69%	71.82%	67.85%	98.34%	75.39%	11.46%	<0.001
0.0075 µM	53.60%	33.10%	46.47%	60.54%	64.39%	68.16%	54.38%	12.99%	<0.001
0.01 µM	49.05%	31.51%	34.40%	45.70%	29.75%	48.29%	39.78%	8.85%	<0.001
0.025 µM				19.02%	11.81%	17.99%	16.27%	3.90%	<0.001

Biomass

Concentration	1st	2nd	3rd	4th	5th	6th	Mean	SD	P-value (vs. Control)
Control	100.00%	100.00%	100.00%	100.00%	100.00%	100.00%	100.00%	0.00%	
0.001 µM	96.26%	74.45%	70.28%				80.33%	13.95%	0.08
0.005 µM	84.16%	85.34%	59.42%	69.84%	78.14%	85.46%	77.06%	10.53%	0.008
0.0075 µM	56.18%	72.18%	29.82%	67.20%	50.03%	41.69%	52.85%	15.83%	<0.001
0.01 µM	45.02%	46.21%	28.07%	37.72%	15.61%	20.28%	32.15%	12.85%	<0.001
0.025 µM				14.12%	11.23%	13.14%	12.83%	1.47%	<0.001

PaTu8988T

Proliferation

Concentration	1st	2nd	3rd	Mean	SD	P-value (vs. Control)
Control	100.00%	100.00%	100.00%	100.00%	0.00%	
0.001µM	77.70%	72.51%	77.48%	75.90%	2.94%	<0.001
0.005µM	53.24%	51.79%	34.44%	46.49%	10.46%	<0.001
0.0075µM	2.16%	7.97%	2.65%	4.26%	3.22%	<0.001
0.01µM	3.60%	6.37%	3.31%	4.43%	1.69%	<0.001

Metabolic activity

Concentration	1st	2nd	3rd	4th	5th	Mean	SD	P-value (vs. Control)
Control	100.00%	100.00%	100.00%	100.00%	100.00%	100.00%	0.00%	
0.001µM	77.87%	95.52%	61.41%	65.32%	115.93%	83.21%	22.62%	0.15
0.005µM		49.72%	57.74%	25.34%	38.42%	42.81%	14.08%	<0.001
0.0075µM		11.49%	14.05%	6.81%	14.73%	11.77%	3.59%	<0.001
0.01µM	9.22%	9.70%	9.24%		9.50%	9.42%	0.23%	<0.001

Biomass

Concentration	1st	2nd	3rd	4th	Mean	SD	P-value (vs. Control)
Control	100.00%	100.00%	100.00%	100.00%	100.00%	0.00%	
0.001µM	87.30%	90.10%	71.03%	82.93%	82.84%	8.41%	0.002
0.005µM	48.27%	42.96%	30.30%	28.94%	37.62%	9.50%	<0.001
0.0075µM	10.73%	11.81%	11.44%	8.90%	10.72%	1.29%	<0.001
0.01µM	8.30%	11.30%	8.84%	7.14%	8.90%	1.75%	<0.001

SU.86.86

Proliferation

Concentration	1st	2nd	3rd	Mean	SD	P-value (vs. Control)
Control	100.00%	100.00%	100.00%	100.00%	0.00%	
0.005 µM	81.82%	95.49%	82.31%	86.54%	7.75%	0.04
0.0075 µM	69.55%	56.39%	66.15%	64.03%	6.83%	<0.001
0.01µM	45.91%	37.59%	50.77%	44.76%	6.67%	<0.001
0.1µM	7.27%	5.26%	5.38%	5.97%	1.13%	<0.001

Metabolic activity

Concentration	1st	2nd	3rd	Mean	SD	P-value (vs. Control)
Control	100.00%	100.00%	100.00%	100.00%	0.00%	
0.005 µM	72.94%	97.83%	100.22%	90.33%	15.11%	0.68
0.0075 µM	70.28%	81.54%	70.79%	74.20%	6.36%	0.06
0.01µM	46.05%	77.87%	50.09%	58.00%	17.32%	0.003
0.1µM	35.78%	26.34%	41.26%	34.46%	7.55%	<0.001

Biomass

Concentration	1st	2nd	3rd	Mean	SD	P-value (vs. Control)
Control	100.00%	100.00%	100.00%	100.00%	0.00%	
0.005 µM	100.30%	88.19%	96.83%	95.11%	6.24%	0.89
0.0075 µM	89.89%	78.96%	65.79%	78.21%	12.07%	0.04
0.01µM	70.20%	57.49%	43.39%	57.03%	13.41%	<0.001
0.1µM	14.63%	9.67%	9.89%	11.40%	2.80%	<0.001

Supplementary Table S3 - Cell Viability Dinaciclib (to 100%Control)

T3M4

	Proliferation						
Concentration	1st	2nd	3rd	4th	Mean	SD	P-value (vs. Control)
Control	100.00%	100.00%	100.00%	100.00%	100.00%	0.00%	
0.001 µM	90.87%	90.57%	91.43%	91.01%	90.97%	0.36%	<0.001
0.0025 µM	80.77%	79.25%	85.36%	80.42%	81.45%	2.69%	<0.001
0.005 µM	77.88%	75.94%	78.21%	73.02%	76.26%	2.38%	<0.001
0.0075 µM	26.44%	26.89%	27.86%	20.90%	25.52%	3.14%	<0.001
	Metabolic activity						
Concentration	1st	2nd	3rd	4th	Mean	SD	P-value (vs. Control)
Control	100.00%	100.00%	100.00%	100.00%	100.00%	0.00%	
0.001 µM	86.69%	85.81%	84.95%	81.93%	84.85%	2.07%	0.008
0.0025 µM	64.11%	73.51%	83.61%	72.47%	73.43%	7.99%	<0.001
0.005 µM	58.09%	58.32%	68.18%	63.37%	61.99%	4.79%	<0.001
0.0075 µM	35.29%	19.68%	40.56%	35.57%	32.78%	9.06%	<0.001
	Biomass						
Concentration	1st	2nd	3rd	4th	Mean	SD	P-value (vs. Control)
Control	100.00%	100.00%	100.00%	100.00%	100.00%	0.00%	
0.001 µM	62.31%	77.39%	95.83%	92.32%	81.96%	15.35%	0.36
0.0025 µM	52.59%	73.37%	95.66%	91.66%	78.32%	19.71%	0.22
0.005 µM	43.37%	69.29%	92.59%	74.47%	69.93%	20.33%	0.06
0.0075 µM	17.39%	36.24%	57.77%	35.71%	36.78%	16.51%	<0.001

Supplementary Table S4 - IC50 Dinaciclib(μM)

	Proliferation	Metabolic Activity	Cell Biomass
**AsPc-1**	0.005229	0.007234	0.005017
**BxPc-3**	0.007975	0.01027	0.008264
**Capan-1**	0.009291	0.01389	0.006561
**Colo357**	0.001253	0.005359	0.002164
**Panc-1**	0.01111	0.02789	0.0139
**PaTu8902**	0.004146	0.004889	0.004156
**PaTu8988S**	0.009124	0.008096	0.007789
**PaTu8988T**	0.004939	0.00317	0.003000
**SU.86.86**	0.009257	0.0335	0.0108
**T3M4**	0.006146	0.005485	0.005237

Supplementary Table S5 - Apoptosis/Necrosis Silmitasertib (%)

AsPc-1

	1st		2nd		3rd		Mean	SD	P-Value (vs. Control)
Concentration	G2(YP+/PI-)	G3(PI+)	G2(YP+/PI-)	G3(PI+)	G2(YP+/PI-)	G3(PI+)	G2+G3		
Control	0.56	4.71	1.94	5.43	1.34	2.14	5.37	1.59	
1μM	0.91	9.95	1.62	10.0	1.42	4.47	9.46	2.54	0.42
5μM	0.55	8.22	0.94	14.5	0.88	9.45	11.51	2.85	0.16
10μM	0.66	18.5	1.31	27.7	1.11	20.1	23.13	4.24	<0.001

BxPc-3

	1st		2nd		3rd		Mean	SD	P-Value (vs. Control)
Concentration	G2(YP+/PI-)	G3(PI+)	G2(YP+/PI-)	G3(PI+)	G2(YP+/PI-)	G3(PI+)	G2+G3		
Control	0.43	2.01	1.98	1.24	1.95	2.02	3.21	0.62	
1μM	0.97	6.17	1.42	3.79	1.59	1.48	5.14	1.66	0.32
2.5μM	0.91	4.94	1.27	3.53	1.17	2.20	4.67	1.02	0.52
5μM	0.77	6.47	0.90	7.01	0.90	4.00	6.68	1.29	0.05

Capan-1

	1st		2nd		3rd		Mean	SD	P-Value (vs. Control)
Concentration	G2(YP+/PI-)	G3(PI+)	G2(YP+/PI-)	G3(PI+)	G2(YP+/PI-)	G3(PI+)	G2+G3		
Control	7.43	10.4	3.59	7.20	2.88	5.47	12.32	4.02	
1μM	8.75	11.9	6.70	7.18	7.30	4.14	15.32	3.90	0.85
5μM	8.84	11.9	5.55	9.10	2.25	6.88	14.84	4.74	0.9
10μM	5.47	19.7	3.68	14.4	2.76	9.30	18.44	5.36	0.44

Colo357

	1st		2nd		3rd		4th		5th		Mean	SD	P-Value (vs. Control)
Concentration	G2(YP+/PI-)	G3(PI+)	G2(YP+/PI-)	G3(PI+)	G2(YP+/PI-)	G3(PI+)	G2(YP+/PI-)	G3(PI+)	G2(YP+/PI-)	G3(PI+)	G2+G3		
Control	2.91	2.23	2.38	1.81	3.01	0.73	1.41	0.90	1.65	0.88	3.58	1.05	
1μM	1.21	0.68	1.72	1.09	0.71	0.60	1.31	0.94	0.80	0.56	1.92	0.56	0.01
5μM	1.41	0.83	1.10	0.83	1.13	0.48	0.59	0.63	0.52	0.38	1.58	0.48	0.002
10μM	1.32	1.40	0.86	0.97	1.89	0.61	0.53	0.82	1.67	0.69	2.15	0.50	0.03

Panc-1

	1st		2nd		3rd		Mean	SD	P-Value (vs. Control)
Concentration	G2(YP+/PI-)	G3(PI+)	G2(YP+/PI-)	G3(PI+)	G2(YP+/PI-)	G3(PI+)	G2+G3		
Control	0.41	1.76	0.36	0.80	0.13	1.25	1.57	0.43	
1μM	0.097	0.70	0.12	1.19	0.00	0.75	0.95	0.25	0.32
5μM	0.17	0.66	0.16	1.93	0.24	1.55	1.57	0.54	>0.99
10μM	0.24	0.72	0.25	1.25	0.00	1.40	1.29	0.23	0.81

PaTu8902

	1st		2nd		3rd		Mean	SD	P-Value (vs. Control)
Concentration	G2(YP+/PI-)	G3(PI+)	G2(YP+/PI-)	G3(PI+)	G2(YP+/PI-)	G3(PI+)	G2+G3		
Control	0.45	0.70	0.41	0.99	0.18	1.35	1.36	0.16	
1μM	0.051	0.60	0.31	1.04	0.25	0.99	1.08	0.31	0.98
5μM	0.046	0.57	0.14	1.02	0.12	1.41	1.10	0.38	0.98
7.5μM	0.041	0.62	0.21	3.49	0.082	1.15	1.86	1.32	0.85
10μM	0.056	1.26	0.10	1.86	0.089	1.78	1.71	0.28	0.95

PaTu8988S

	1st		2nd		3rd		Mean	SD	P-Value (vs. Control)
Concentration	G2(YP+/PI-)	G3(PI+)	G2(YP+/PI-)	G3(PI+)	G2(YP+/PI-)	G3(PI+)	G2+G3		
Control	2.09	11.7	2.04	12.8	3.68	12.1	14.80	0.81	
1μM	1.93	6.24	2.87	11.3	4.69	8.30	11.78	2.60	>0.99
5μM	1.36	4.51	1.11	11.7	1.87	6.13	8.89	2.90	0.18
7.5μM	1.18	6.31	0.59	11.7	1.41	4.37	8.52	2.76	0.09
10μM	0.91	6.38	0.40	14.5	1.19	6.07	9.82	3.59	0.40

PaTu8988T

	1st		2nd		3rd		Mean	SD	P-Value (vs. Control)
Concentration	G2(YP+/PI-)	G3(PI+)	G2(YP+/PI-)	G3(PI+)	G2(YP+/PI-)	G3(PI+)	G2+G3		
Control	0.053	0.99	0.23	0.57	0.098	1.34	1.09	0.26	
1μM	0.032	1.00	0.17	0.74	0.062	1.21	1.07	0.15	>0.99
5μM	0.031	0.87	0.069	0.76	0.048	1.20	0.99	0.18	0.99
7.5μM	0.01	1.00	0.00687	0.94	0.062	1.25	1.09	0.16	>0.99
10μM	0.036	1.20	0.02	0.91	0.055	1.95	1.39	0.45	0.65

SU.86.86

	1st		2nd		3rd		Mean	SD	P-Value (vs. Control)
Concentration	G2(YP+/PI-)	G3(PI+)	G2(YP+/PI-)	G3(PI+)	G2(YP+/PI-)	G3(PI+)	G2+G3		
Control	2.15	3.09	1.9	5.26	1.71	3.91	6.01	0.83	
1μM	0.89	3.06	1.54	4.24	2.69	4.62	5.68	1.37	0.96
5μM	0.86	2.97	1.29	2.77	2.03	2.31	4.08	0.21	0.10
10μM	1.38	4.20	1.18	4.15	1.92	3.48	5.44	0.11	0.82

T3M4

	1st		2nd		3rd		Mean	SD	P-Value (vs. Control)
Concentration	G2(YP+/PI-)	G3(PI+)	G2(YP+/PI-)	G3(PI+)	G2(YP+/PI-)	G3(PI+)	G2+G3		
Control	4.04	2.76	6.13	5.88	2.75	5.85	9.14	2.16	
1μM	2.44	1.54	7.27	2.14	2.12	2.03	5.85	2.52	0.89
5μM	2.44	2.34	2.49	5.74	1.51	2.85	5.79	1.73	0.88
7.5μM	2.64	5.65	2.59	16.3	2.41	10.3	13.30	4.35	0.79
10μM	3.5	14.3	5.26	33.9	6.12	24.9	29.33	8.80	0.005

G2: Apoptosis
G3: Necrosis
G2+G3: Cell Deaths

Supplementary Table S6 - Apoptosis/Necrosis Dinaciclib (%)

AsPc-1

	1st		2nd		3rd		Mean	SD	P-Value (vs. Control)
Concentration	G2(YP+/PI-)	G3(PI+)	G2(YP+/PI-)	G3(PI+)	G2(YP+/PI-)	G3(PI+)	G2+G3		
Control	1.08	2.75	1.83	3.77	0.97	2.31	4.24	0.99	
0.005μM	2.34	6.83	1.35	10.7	0.90	3.24	8.45	3.27	0.29
0.0075μM	2.55	11.1	1.52	12.5	1.54	6.03	11.75	2.96	0.04
0.01μM	2.14	16.8	2.10	18.8	3.86	20.5	21.40	2.24	<0.001

BxPc-3

	1st		2nd		3rd		Mean	SD	P-Value (vs. Control)
Concentration	G2(YP+/PI-)	G3(PI+)	G2(YP+/PI-)	G3(PI+)	G2(YP+/PI-)	G3(PI+)	G2+G3		
Control	0.46	1.82	1.55	1.75	1.31	2.82	3.24	0.76	
0.005μM	1.35	6.64	4.04	4.33	1.68	2.19	6.74	2.04	0.39
0.0075μM	1.08	10.3	3.57	6.95	1.64	2.82	8.79	3.08	0.12
0.01μM	1.15	15.7	2.16	17.7	2.91	9.54	16.39	3.04	0.002

Capan-1

	1st		2nd		3rd		4th		Mean	SD	P-Value (vs. Control)
Concentration	G2(YP+/PI-)	G3(PI+)	G2(YP+/PI-)	G3(PI+)	G2(YP+/PI-)	G3(PI+)	G2(YP+/PI-)	G3(PI+)	G2+G3		
Control	4.28	5.17	4.59	7.47	4.74	7.33	3.16	7.26	11.00	1.12	
0.001μM	5.20	6.33	5.23	3.75	5.23	8.22	3.14	7.87	11.24	1.59	>0.99
0.01μM	8.01	17.3	6.79	10.7	6.31	12.9	10.9	11.1	21.00	2.96	<0.001
0.1μM	7.71	37.2	7.09	41.8	8.09	37.6	12.3	34.9	46.67	1.52	<0.001

Colo357

	1st		2nd		3rd		4th		5th		Mean	SD	P-Value (vs. Control)
Concentration	G2(YP+/PI-)	G3(PI+)	G2(YP+/PI-)	G3(PI+)	G2(YP+/PI-)	G3(PI+)	G2(YP+/PI-)	G3(PI+)	G2(YP+/PI-)	G3(PI+)	G2+G3		
Control	2.50	2.14	2.41	1.41	1.42	0.96	1.04	1.06	1.60	0.99	3.11	0.97	
0.001μM	3.85	1.60	4.65	3.13	4.47	1.01	1.86	1.71	1.20	1.45	4.99	1.77	0.37
0.01μM	5.06	2.00	4.82	5.51	4.00	2.84	3.21	2.87	2.91	4.71	7.59	1.46	0.01
0.05μM	1.96	12.7	1.70	10.4	2.89	12.3	2.02	7.01	2.22	14.8	13.60	2.77	<0.001

Panc-1

	1st		2nd		3rd		4th		Mean	SD	P-Value (vs. Control)
Concentration	G2(YP+/PI-)	G3(PI+)	G2(YP+/PI-)	G3(PI+)	G2(YP+/PI-)	G3(PI+)	G2(YP+/PI-)	G3(PI+)	G2+G3		
Control	0.98	0.72	0.94	0.71	0.28	2.26	0.40	2.71	2.25	0.61	
0.001μM	0.48	1.92	0.50	1.84	0.39	6.29	0.46	9.79	5.42	3.30	0.63
0.01μM	1.53	1.43	1.61	1.43	0.96	6.16	1.00	8.44	5.64	2.76	0.58
0.05μM	10.6	3.60	10.5	3.09	12.4	14.9	0.48	10.7	16.57	6.30	0.002

PaTu8902

	1st		2nd		3rd		Mean	SD	P-Value (vs. Control)
Concentration	G2(YP+/PI-)	G3(PI+)	G2(YP+/PI-)	G3(PI+)	G2(YP+/PI-)	G3(PI+)	G2+G3		
Control	0.51	1.01	1.15	1.88	0.10	1.17	1.94	0.78	
0.003μM	0.26	1.81	0.55	3.17	0.19	0.41	2.13	1.27	>0.99
0.005μM	0.59	1.77	0.94	3.71	0.20	1.05	2.75	1.42	>0.99
0.006μM	0.65	3.29	2.75	12.1	0.31	1.34	6.81	5.76	0.75
0.0075μM	1.10	7.69	4.15	15.5	0.82	2.84	10.70	6.67	0.28
0.01μM	3.62	19.5	5.27	27.8	5.96	9.50	23.88	7.21	0.002

PaTu8988S

	1st		2nd		3rd		Mean	SD	P-Value (vs. Control)
Concentration	G2(YP+/PI-)	G3(PI+)	G2(YP+/PI-)	G3(PI+)	G2(YP+/PI-)	G3(PI+)	G2+G3		
Control	4.02	18.2	3.03	10.1	3.87	16.8	18.67	3.97	
0.005μM	4.23	9.84	2.00	5.21	3.60	9.40	11.43	3.01	0.04
0.0075μM	3.63	9.95	2.02	9.82	3.16	7.65	12.08	1.14	0.07
0.01μM	3.36	10.1	2.32	10.2	2.88	6.41	11.76	1.79	0.05
0.05μM	2.33	10.9	1.41	11.0	1.60	12.4	13.21	0.65	0.14

PaTu8988T

	1st		2nd		3rd		4th		Mean	SD	P-Value (vs. Control)
Concentration	G2(YP+/PI-)	G3(PI+)	G2(YP+/PI-)	G3(PI+)	G2(YP+/PI-)	G3(PI+)	G2(YP+/PI-)	G3(PI+)	G2+G3		
Control	0.38	2.6	6.36	4.65	0.42	1.65	0.26	3.81	5.03	3.52	
0.003μM	0.14	1.61	4.03	6.08	0.28	1.65	0.32	2.18	4.07	3.50	>0.99
0.005μM	0.25	2.19	3.41	7.34	0.51	2.45	0.49	3.85	5.12	3.32	>0.99
0.006μM	0.35	2.3	3.72	9.30	0.56	2.80	0.91	4.76	6.18	4.11	>0.99
0.01μM	0.83	9.23	4.54	22.2	1.71	5.03	7.49	12.60	15.91	7.95	0.38

SU.86.86

	1st		2nd		3rd		Mean	SD	P-Value (vs. Control)
Concentration	G2(YP+/PI-)	G3(PI+)	G2(YP+/PI-)	G3(PI+)	G2(YP+/PI-)	G3(PI+)	G2+G3		
Control	3.82	3.94	4.02	6.45	2.70	6.10	9.01	1.12	
0.005μM	6.06	6.96	3.67	5.97	2.86	4.89	10.14	2.18	0.85
0.01μM	3.98	6.77	3.74	11.3	2.30	8.71	12.27	1.96	0.21
0.1μM	4.74	20.7	4.46	24.0	2.51	23.4	26.60	1.33	<0.001

T3M4

	1st		2nd		3rd		Mean	SD	P-Value (vs. Control)
Concentration	G2(YP+/PI-)	G3(PI+)	G2(YP+/PI-)	G3(PI+)	G2(YP+/PI-)	G3(PI+)	G2+G3		
Control	3.51	7.45	4.90	5.44	5.44	5.72	10.82	0.35	
0.003μM	4.53	4.93	5.80	4.77	5.55	3.65	9.74	0.59	0.90
0.005μM	3.16	5.63	3.93	4.64	4.53	3.55	8.48	0.30	0.43
0.0075μM	6.09	11.7	5.98	12.6	6.29	8.05	16.90	1.84	0.01
0.01μM	9.13	13.8	10.6	19.0	11.6	12.3	25.48	2.94	<0.001

G2: Apoptosis
G3: Necrosis
G2+G3: Cell Deaths

Supplementary Table S7 - Target Gene Expression (Log2(TPM+1))

	Silmitasertib Target Genes			Dinaciclib Target Genes			
	CSNK2A1	*CSNK2A2*	*CSNK2B*	*CDK1*	*CDK2*	*CDK5*	*CDK9*
**AsPc-1**	6.50	5.46	6.53	7.01	5.03	5.04	4.95
**BxPc-3**	6.75	5.44	6.78	7.25	5.54	5.32	5.65
**Capan-1**	6.59	5.42	7.52	5.73	5.42	5.68	5.32
**Colo357**	6.76	5.51	6.73	6.50	4.37	3.96	4.57
**Panc-1**	7.69	5.41	7.70	7.21	5.61	5.10	4.87
**SU.86.86**	5.83	5.68	7.28	6.71	5.31	4.08	5.37
**PaTu8988S**	6.99	5.63	7.26	7.14	5.17	4.53	5.47
**PaTu8988T**	7.01	6.47	7.45	7.55	6.19	4.01	5.28
**PaTu8902**	7.15	6.42	7.41	8.51	6.85	3.51	5.38
**T3M4**	6.91	6.12	7.07	6.33	6.06	3.98	6.63
**Control**	3.63	4.43	6.00	0.41	2.83	1.98	4.50

Supplementary Table S8 - Silmitasertib Target Gene Variants in PDAC Cell Lines

Cell line	#Chromesome	Start	End	Reference	Observed	Zygosities	Variant Confidance	Variant Allele Frequency	Reading Depth	Gene	Base Change	Animo Acin Change	Variant Type
BxPc-3	chr20	464502	464502	G	T	het	74.77	33.3	18	*CSNK2A1*	NM_001895.3:c.*103C>A	-	3_prime_UTR_variant
BxPc-3	chr20	468419	468419	G	A	het	10.43	66.7	3	*CSNK2A1*	NM_001895.3:c.825-200C>T	-	intron_variant
BxPc-3	chr20	480619	480621	AA	T	hom	804.73	100	64	*CSNK2A1*	NM_001895.3:c.214-42_214-41delTTinsA	-	intron_variant
BxPc-3	chr20	483933	483933	C	T	het	187.77	61.5	13	*CSNK2A1*	NM_001895.3:c.213+1829G>A	-	intron_variant
Capan-1	chr20	464502	464502	G	T	het	63.77	23.5	17	*CSNK2A1*	NM_001895.3:c.*103C>A	-	3_prime_UTR_variant
Capan-1	chr20	467240	467240	T	C	het	161.77	66.7	12	*CSNK2A1*	NM_001895.3:c.974-134A>G	-	intron_variant
Panc-1	chr20	464502	464502	G	T	het	24.78	37.5	8	*CSNK2A1*	NM_001895.3:c.*103C>A	-	3_prime_UTR_variant
Panc-1	chr20	480619	480621	AA	T	hom	564.73	100	41	*CSNK2A1*	NM_001895.3:c.214-42_214-41delTTinsA	-	intron_variant
Panc-1	chr20	483933	483933	C	T	hom	473.77	100	15	*CSNK2A1*	NM_001895.3:c.213+1829G>A	-	intron_variant
PaTu8902	chr20	464502	464502	G	T	het	36.77	33.3	15	*CSNK2A1*	NM_001895.3:c.*103C>A	-	3_prime_UTR_variant
PaTu8988T	chr20	464502	464502	G	T	het	36.77	23.1	13	*CSNK2A1*	NM_001895.3:c.*103C>A	-	3_prime_UTR_variant
PaTu8988T	chr20	485995	486020	GTT	G	hom	42.45	66.7	3	*CSNK2A1*	NM_001895.3:c.102-124_102-123delAA	-	intron_variant
PaTu8988S	chr20	464502	464502	G	T	het	43.77	27.3	11	*CSNK2A1*	NM_001895.3:c.*103C>A	-	3_prime_UTR_variant
T3M4	chr20	480619	480621	AA	T	hom	453.73	100	29	*CSNK2A1*	NM_001895.3:c.214-42_214-41delTTinsA	-	intron_variant
BxPc-3	chr16	58204541	58204541	C	A	hom	181.84	100	6	*CSNK2A2*	NM_001896.2:c.430-1944G>T	-	intron_variant
PaTu8988T	chr16	58200863	58200863	T	C	het	12.05	66.7	3	*CSNK2A2*	NM_001896.2:c.726+248A>G	-	intron_variant
PaTu8988T	chr16	58208602	58208602	G	A	hom	29.12	100	3	*CSNK2A2*	NM_001896.2:c.370-188C>T	-	intron_variant
PaTu8988T	chr16	58208605	58208605	G	A	het	10.48	66.7	3	*CSNK2A2*	NM_001896.2:c.370-191C>T	-	intron_variant
PaTu8988S	chr16	58208605	58208616	G	GA	het	28.17	66.7	3	*CSNK2A2*	NM_001896.2:c.370-192dupT	-	intron_variant
T3M4	chr16	58200616	58200618	CA	TC	het	253.75	39	41	*CSNK2A2*	NM_001896.2:c.727-29_727-28delTGinsGA	-	intron_variant
T3M4	chr16	58200946	58200948	CG	C	het	97.76	80	5	*CSNK2A2*	NM_001896.2:c.726+164delC	-	intron_variant
T3M4	chr16	58230796	58230796	A	C	het	164.77	28.1	32	*CSNK2A2*	NM_001896.2:c.105-48T>G	-	intron_variant
AsPc-1	chr6	31635960	31635968	ATTCCAAA	C	hom	39.48	100	3	*CSNK2B*	NM_001320.6:c.175+213_175+220delATTCCAAAinsC	-	upstream_gene_variant
T3M4	chr6	31635960	31635968	ATTCCAAA	C	hom	78.03	100	4	*CSNK2B*	NM_001320.6:c.175+213_175+220delATTCCAAAinsC	-	upstream_gene_variant

Supplementary Table S9 - Dinaciclib Target Gene Variants in PDAC Cell Lines

Cell line	#Chromesome	Start	End	Reference	Observed	Zygosities	Variant Confidance	Variant Allele Frequency	Reading Depth	Gene	Base Change	Animo Acin Change	Variant Type
BxPc-3	chr10	62551889	62551891	AA	GT	hom	2437.28	100	89	*CDK1*	NM_001320918.1:c.654-17_654-16delAAinsGT	-	intron_variant
Capan-1	chr10	62551889	62551891	AA	GT	het	868.19	34.3	143	*CDK1*	NM_001320918.1:c.654-17_654-16delAAinsGT	-	intron_variant
Colo357	chr10	62544705	62544705	A	G	het	288.77	36.7	30	*CDK1*	NM_001320918.1:c.194+86A>G	-	intron_variant
Colo357	chr10	62551889	62551891	AA	GT	het	3363.03	100	90	*CDK1*	NM_001320918.1:c.654-17_654-16delAAinsGT	-	intron_variant
Panc-1	chr10	62551889	62551891	AA	GT	hom	2701.24	100	101	*CDK1*	NM_001320918.1:c.654-17_654-16delAAinsGT	-	intron_variant
PaTu8902	chr10	62551889	62551891	AA	GT	hom	2325.28	100	82	*CDK1*	NM_001320918.1:c.654-17_654-16delAAinsGT	-	intron_variant
PaTu8988T	chr10	62551889	62551891	AA	GT	hom	2602.46	100	96	*CDK1*	NM_001320918.1:c.654-17_654-16delAAinsGT	-	intron_variant
PaTu8988S	chr10	62551889	62551891	AA	GT	hom	2186.77	100	80	*CDK1*	NM_001320918.1:c.654-17_654-16delAAinsGT	-	intron_variant
SU.86.86	chr10	62551889	62551891	AA	GT	hom	1368.8	100	52	*CDK1*	NM_001320918.1:c.654-17_654-16delAAinsGT	-	intron_variant
BxPc-3	chr12	56364719	56364719	T	A	het	10.5	30	10	*CDK2*	NM_001798.4:c.589-109T>A	-	upstream_gene_variant
Colo357	chr12	56360765	56360765	C	T	hom	1693.77	100	71	*CDK2*	NM_001798.4:c.-28C>T	-	5_prime_UTR_variant
Colo357	chr12	56361521	56361527	TTC	T	hom	571.73	100	14	*CDK2*	NM_001798.4:c.117-115_117-114delTC	-	upstream_gene_variant
Colo357	chr12	56362111	56362111	A	G	hom	92.03	100	5	*CDK2*	NM_001798.4:c.315+158A>G	-	upstream_gene_variant
Panc-1	chr7	150751809	150751809	C	A	het	12.05	66.7	3	*CDK5*	NM_004935.3:c.651-257G>T	-	upstream_gene_variant
Colo357	chr9	130548593	130548595	C	CCA	het	33.47	72.7	11	*CDK9*	NM_001261.3:c.92+75_92+76insAC	-	downstream_gene_variant

Supplementary Table S10 - *KRAS* Variants in PDAC Cell Lines

Cell line	#Chromesome	Start	End	Reference	Observed	Zygosities	Variant Confidance	Variant Allele Frequency	Reading Depth	Gene	Base Change	Animo Acin Change	Variant Type
AsPc-1	chr12	25398284	25398284	C	T	hom	2219.77	100	90	*KRAS*	NM_033360.2:c.35G>A	NM_033360.2:p.Gly12Asp	missense_variant
Capan-1	chr12	25398284	25398284	C	A	hom	1774.77	97.1	70	*KRAS*	NM_033360.2:c.35G>T	NM_033360.2:p.Gly12Val	missense_variant
Colo357	chr12	25398284	25398284	C	T	het	403.77	23.8	126	*KRAS*	NM_033360.2:c.35G>A	NM_033360.2:p.Gly12Asp	missense_variant
Panc-1	chr12	25398284	25398284	C	T	het	2675.77	62.1	203	*KRAS*	NM_033360.2:c.35G>A	NM_033360.2:p.Gly12Asp	missense_variant
PaTu8902	chr12	25398284	25398284	C	A	hom	1052.77	100	42	*KRAS*	NM_033360.2:c.35G>T	NM_033360.2:p.Gly12Val	missense_variant
PaTu8988T	chr12	25398284	25398284	C	A	hom	1212.77	98	49	*KRAS*	NM_033360.2:c.35G>T	NM_033360.2:p.Gly12Val	missense_variant
PaTu8988S	chr12	25398284	25398284	C	A	hom	1541.77	96.9	65	*KRAS*	NM_033360.2:c.35G>T	NM_033360.2:p.Gly12Val	missense_variant
SU.86.86	chr12	25398284	25398284	C	T	het	6018.77	83.7	319	*KRAS*	NM_033360.2:c.35G>A	NM_033360.2:p.Gly12Asp	missense_variant
T3M4	chr12	25380275	25380275	A	C	het	709.77	32.6	129	*KRAS*	NM_033360.2:c.183A>C	NM_033360.2:p.Gln61His	missense_variant

Supplementary Table S11 - *TP53* Variants in PDAC Cell Lines

Cell line	#Chromesome	Start	End	Reference	Observed	Zygosities	Variant Confidance	Variant Allele Frequency	Reading Depth	Gene	Base Change	Animo Acin Change	Variant Type
AsPc-1	chr17	7578526	7578530	CA	C	hom	3365.73	96.4	110	*TP53*	NM_000546.4:c.403delT	NM_000546.4:p.Cys135fs	frameshift_variant
Panc-1	chr17	7577120	7577120	C	T	hom	1977.77	98.8	81	*TP53*	NM_000546.4:c.818G>A	NM_000546.4:p.Arg273His	missense_variant
BxPc-3	chr17	7578190	7578190	T	C	hom	2653.77	99	103	*TP53*	NM_000546.4:c.659A>G	NM_000546.4:p.Tyr220Cys	missense_variant
Capan-1	chr17	7578454	7578454	G	A	hom	2094.77	100	83	*TP53*	NM_000546.4:c.476C>T	NM_000546.4:p.Ala159Val	missense_variant
Colo357	chr17	7579419	7579424	AG	A	hom	4167.73	100	130	*TP53*	NM_000546.4:c.267delC	NM_000546.4:p.Ser90fs	frameshift_variant
PaTu8902	chr17	7577094	7577094	G	A	hom	2250.77	100	79	*TP53*	NM_000546.4:c.844C>T	NM_000546.4:p.Arg282Trp	missense_variant
PaTu8988T	chr17	7577094	7577094	G	A	hom	1503.77	100	57	*TP53*	NM_000546.4:c.844C>T	NM_000546.4:p.Arg282Trp	missense_variant
PaTu8988S	chr17	7577094	7577094	G	A	hom	2498.77	100	97	*TP53*	NM_000546.4:c.844C>T	NM_000546.4:p.Arg282Trp	missense_variant
SU.86.86	chr17	7573948	7573948	C	A	hom	665.77	100	24	*TP53*	NM_000546.4:c.1079G>T	NM_000546.4:p.Gly360Val	missense_variant
SU.86.86	chr17	7577548	7577548	C	T	hom	1415.77	100	54	*TP53*	NM_000546.4:c.733G>A	NM_000546.4:p.Gly245Ser	missense_variant
T3M4	chr17	7578190	7578190	T	C	hom	2061.77	100	78	*TP53*	NM_000546.4:c.659A>G	NM_000546.4:p.Tyr220Cys	missense_variant

Supplementary Table S12 - Gene expression *KRAS* (Log_2(TPM+1))

	***KRAS* Expression**
**AsPc-1**	4.79
**BxPc-3**	4.53
**Capan-1**	4.40
**Colo357**	4.16
**Panc-1**	6.11
**PaTu8902**	4.51
**PaTu8988S**	4.65
**PaTu8988T**	4.46
**SU.86.86**	7.09
**T3M4**	5.79
**Control**	2.14

Supplementary Table S13 - Gene expression *TP53* (Log_2(TPM+1))

	TP53 Expression
**AsPc-1**	1.24
**BxPc-3**	5.42
**Capan-1**	4.39
**Colo357**	2.13
**Panc-1**	5.29
**PaTu8902**	5.37
**PaTu8988S**	5.35
**PaTu8988T**	5.34
**SU.86.86**	4.61
**T3M4**	5.26
**Control**	2.83
